# The UK Biobank resource with deep phenotyping and genomic data

**DOI:** 10.1038/s41586-018-0579-z

**Published:** 2018-10-10

**Authors:** Clare Bycroft, Colin Freeman, Desislava Petkova, Gavin Band, Lloyd T. Elliott, Kevin Sharp, Allan Motyer, Damjan Vukcevic, Olivier Delaneau, Jared O’Connell, Adrian Cortes, Samantha Welsh, Alan Young, Mark Effingham, Gil McVean, Stephen Leslie, Naomi Allen, Peter Donnelly, Jonathan Marchini

**Affiliations:** 1 Wellcome Centre for Human Genetics, University of Oxford, Oxford, UK; 2 Department of Statistics, University of Oxford, Oxford, UK; 3 Melbourne Integrative Genomics and the Schools of Mathematics and Statistics, and BioSciences, The University of Melbourne, Parkville, Victoria, Australia; 4 Murdoch Children’s Research Institute, Parkville, Victoria, Australia; 5 Department of Genetic Medicine and Development, University of Geneva, Geneva, Switzerland; 6 Swiss Institute of Bioinformatics, University of Geneva, Geneva, Switzerland; 7 Institute of Genetics and Genomics in Geneva, University of Geneva, Geneva, Switzerland; 8 Illumina Ltd, Chesterford Research Park, Little Chesterford, Essex, UK; 9 Nuffield Department of Clinical Neurosciences, Division of Clinical Neurology, John Radcliffe Hospital, University of Oxford, Oxford, UK; 10 UK Biobank, Adswood, Stockport, Cheshire, UK; 11 Big Data Institute, Li Ka Shing Centre for Health Information and Discovery, University of Oxford, Oxford, UK; 12 Present Address: Procter & Gamble, Brussels, Belgium

**Keywords:** Deep Phenotyping, Genotype Imputation, Genetic Investigation Of Anthropometric Traits (GIANT), Pseudo-autosomal Region (PAR), Acceptance Set, Genome-wide association studies, Genome, Population genetics, Genotype, Haplotypes

## Abstract

The UK Biobank project is a prospective cohort study with deep genetic and phenotypic data collected on approximately 500,000 individuals from across the United Kingdom, aged between 40 and 69 at recruitment. The open resource is unique in its size and scope. A rich variety of phenotypic and health-related information is available on each participant, including biological measurements, lifestyle indicators, biomarkers in blood and urine, and imaging of the body and brain. Follow-up information is provided by linking health and medical records. Genome-wide genotype data have been collected on all participants, providing many opportunities for the discovery of new genetic associations and the genetic bases of complex traits. Here we describe the centralized analysis of the genetic data, including genotype quality, properties of population structure and relatedness of the genetic data, and efficient phasing and genotype imputation that increases the number of testable variants to around 96 million. Classical allelic variation at 11 human leukocyte antigen genes was imputed, resulting in the recovery of signals with known associations between human leukocyte antigen alleles and many diseases.

## Abstract (web summary)

Deep phenotype and genome-wide genetic data from 500,000 individuals from the UK Biobank, describing population structure and relatedness in the cohort, and imputation to increase the number of testable variants to 96 million.

## Main

Understanding the role that genetics has in phenotypic and disease variation, and its potential interactions with other factors, is crucial for a better understanding of human biology. It is hoped that this will lead to more successful drug development^1^, and potentially to more efficient and personalized treatments. As such, a key component of the UK Biobank resource has been the collection of genome-wide genetic data on every participant using a purpose-designed genotyping array^2^. An interim release of genotype data on approximately 150,000 UK Biobank participants in May 2015^3^ has already facilitated numerous studies^4–6^.

In this paper, we summarize the existing and planned content of the phenotype resource and describe the genetic dataset on the full 500,000 participants. To facilitate its wider use, we applied a range of quality control procedures and conducted a set of analyses that reveal properties of the genetic data—such as population structure and relatedness—that can be important for downstream analyses. In addition, we estimated haplotypes and imputed genotypes into the dataset that increases the number of testable variants by more than 100-fold to approximately 96 million variants. We also imputed classical allelic variation at 11 human leukocyte antigen (HLA) genes, and replicated signals of known associations between HLA alleles and many common diseases. We describe tools that allow efficient genome-wide association studies (GWAS) of multiple traits and fast phenome-wide association studies, which work together with a new compressed file format that has been used to distribute the dataset. As a further check of the genotyped and imputed datasets, we performed a test-case genome-wide association scan on a well-studied human trait, standing height.

## The UK Biobank

**Fig. 1:**
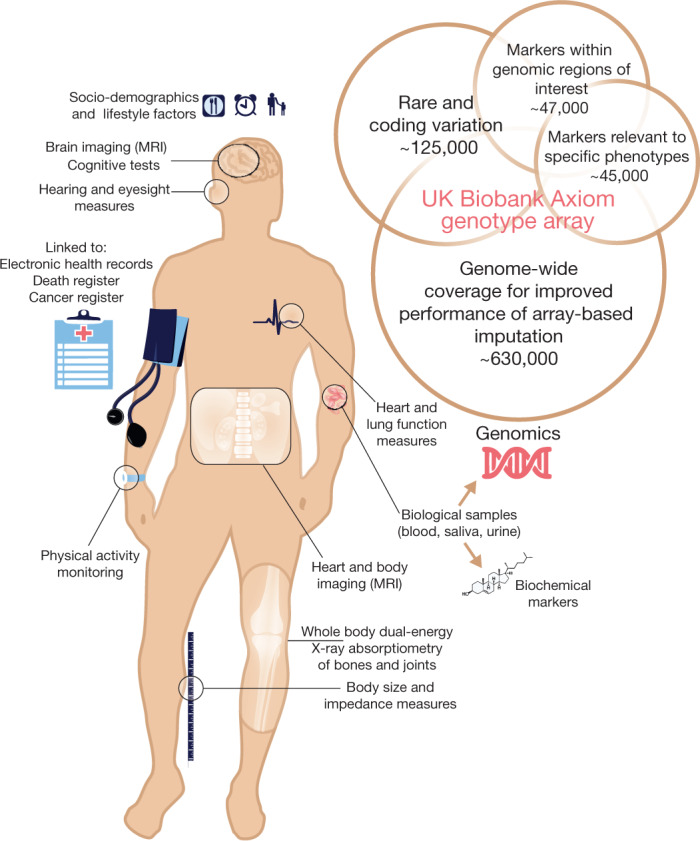
Summary of the UK Biobank resource and genotyping array content. Summary of the major components of the UK Biobank resource. See Extended Data Table 1 for more details. The figure also shows a schematic representation of the different categories of content on the UK Biobank Axiom genotype array. Numbers indicate the approximate count of markers within each category, ignoring any overlap. A more detailed description of the array content is available in the UK Biobank Axiom Array Content Summary^2^.

A wide variety of phenotypic information as well as biological samples have been collected for each of the approximately 500,000 UK Biobank participants (Fig. 1). At recruitment, participants provided electronic signed consent, answered questions on socio-demographic, lifestyle and health-related factors, and completed a range of physical measures (see Extended Data Table 1). They also provided blood, urine and saliva samples, which were stored in such a way as to allow many different types of assay to be performed (for example, genetic, proteomic and metabonomic analyses)^7^. Once recruitment was fully underway, further enhancements were introduced to the assessment visit, including a range of eye measures, an electrocardiograph test, arterial stiffness and a hearing test.

The baseline information has been, and will continue to be, extended in several ways. For example, repeat assessments are planned to be conducted in subsets of the cohort every few years, to enable calibration of measurements, adjustment for regression dilution, and estimation of longitudinal change. Objective measures of physical activity have also been collected (using a tri-axial accelerometer) in 100,000 participants in 2013–2014^8^ with repeated measures being collected over a period of a year (on a seasonal basis) from 2,500 of these participants. A multi-modal imaging assessment is currently underway, which comprises magnetic resonance imaging (MRI) of the brain^9^, heart^10^ and body, carotid ultrasound^11^ and a whole body dual-energy X-ray absorptiometry of the bones and joints^12^. Data collection started in 2014 and is anticipated to take 7–8 years to achieve imaging for 100,000 participants in dedicated imaging assessment centres across the United Kingdom, with repeat imaging measures being planned for a subset of participants.

All participants provided consent for follow-up through linkage to their health-related records. As of May 2018, there were over 14,000 deaths, 79,000 participants with cancer diagnoses, and 400,000 participants with at least one hospital admission. Considerable efforts are now underway to incorporate data from a range of other national datasets including primary care, screening programmes, and disease-specific registries, as well as asking participants directly about health-related outcomes through online questionnaires (see Extended Data Table 1). Efforts are also underway to develop scalable approaches that can characterize in detail different health outcomes by cross-referencing multiple sources of coded clinical information^13^.

Measurements for a wide range of biochemical markers of key interest to the research community have also been carried out, including those that have known associations with disease (for example, lipids for vascular disease and sex hormones for cancer), diagnostic value (for example, HbA_1c_ for diabetes and rheumatoid factor for arthritis), or the ability to characterize phenotypes not otherwise well assessed (for example, biomarkers for renal and liver function).

UK Biobank is an open-access resource that encourages researchers from around the world, including those from the academic, charity, public and commercial sectors, to access the data for any health-related research that is in the public interest.

## Whole-genome genotyping

The UK Biobank genetic data contains genotypes for 488,377 participants. These were assayed using two very similar genotyping arrays. A subset of 49,950 participants involved in the UK Biobank Lung Exome Variant Evaluation (UK BiLEVE) study were genotyped at 807,411 markers using the Applied Biosystems UK BiLEVE Axiom Array by Affymetrix (now part of Thermo Fisher Scientific), which is described elsewhere^6^. Following this, 438,427 participants were genotyped using the closely related Applied Biosystems UK Biobank Axiom Array (825,927 markers) that shares 95% of marker content with the UK BiLEVE Axiom Array. The marker content of the UK Biobank Axiom array was chosen to capture genome-wide genetic variation (single nucleotide polymorphism (SNPs) and short insertions and deletions (indels)), and is summarized in Fig. 1. Many markers were included because of known associations with, or possible roles in, disease. The array also includes coding variants across a range of minor allele frequencies (MAFs), including rare markers (<1% MAF); and markers that provide good genome-wide coverage for imputation in European populations in the common (>5%) and low frequency (1–5%) MAF ranges. Further details of the array design are in the UK Biobank Axiom Array Content Summary^2^.

DNA was extracted from stored blood samples that had been collected from participants on their visit to a UK Biobank assessment centre. Genotyping was carried out by Affymetrix Research Services Laboratory in 106 sequential batches of approximately 4,700 samples (see Methods, Supplementary Table 12). Affymetrix applied a custom genotype calling pipeline and quality filtering optimized for biobank-scale genotyping experiments and the novel genotyping arrays, which contain markers that had not been previously typed using Affymetrix technology (see Methods). This resulted in a set of genotype calls for 489,212 samples at 812,428 unique markers (biallelic SNPs and indels) from both arrays, with which we conducted further quality control and analysis (Extended Data Table 2).

Our quality control pipeline was designed specifically to accommodate the large-scale dataset of ethnically diverse participants, genotyped in many batches, using two slightly different arrays, and which will be used by many researchers to tackle a wide variety of research questions. Participants reported their ethnic background by selecting from a fixed set of categories^14^. Although most (94%) individuals report their ethnic background as within the broad-level group ‘white’, there are still approximately 22,000 individuals with a self-reported ethnic background originating outside Europe (Extended Data Table 3). We used approaches based on principal component analysis (PCA) to account for population structure in both marker and sample-based quality control (see Methods).

**Extended Data Fig. 1:**
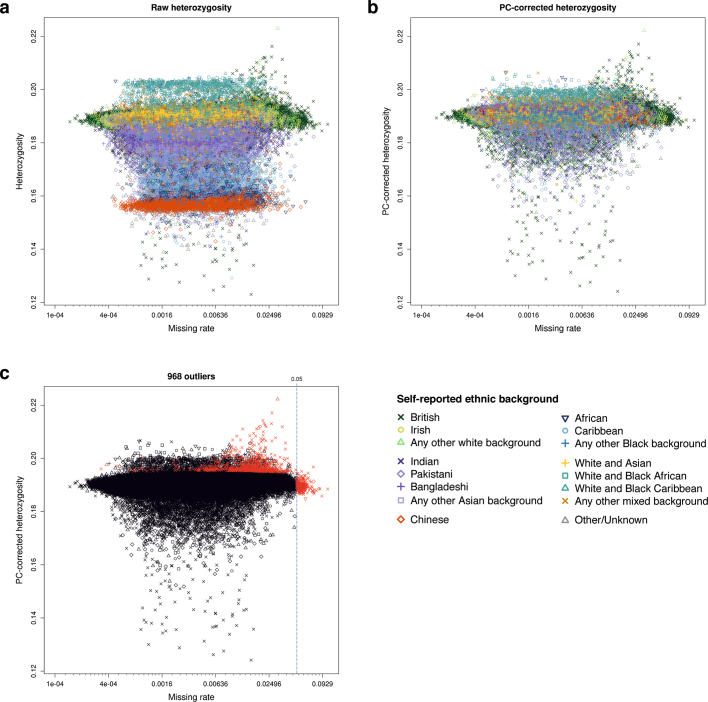
Summary of sample-based quality control. **a**–**c**, The three plots show heterozygosity and missing rates, which we used to flag poor quality samples (*n* = 488,377 samples). Panels **a** and **b** show heterozygosity for each sample before and after, respectively, correcting for ancestral background using principal components. The symbols (shapes and colours) indicate the self-reported ethnic background of each participant. Panel **c** shows the set of 968 samples we flagged as outliers (in red), and all other samples (in black), with shapes the same as for the other two plots. The vertical line shows the threshold we used to call samples as outliers on missing rate. In all plots missing rate data are transformed to the logit scale, but with the axis annotated with the original values.

To identify poor quality markers, we used statistical tests designed primarily to check for consistency across experimental factors, such as array or batch (see Methods; Extended Data Table 4). As a result of these tests, we set to missing 0.97% of all the genotype calls made by Affymetrix. We identified poor quality samples using the metrics of missing rate and heterozygosity adjusted for population structure (Extended Data Fig. 1), as extreme values in one or both of these metrics can be indicators of poor sample quality due to, for example, DNA contamination^15^. We identified 968 such samples (0.2%), and provide this list to researchers.

**Fig. 2:**
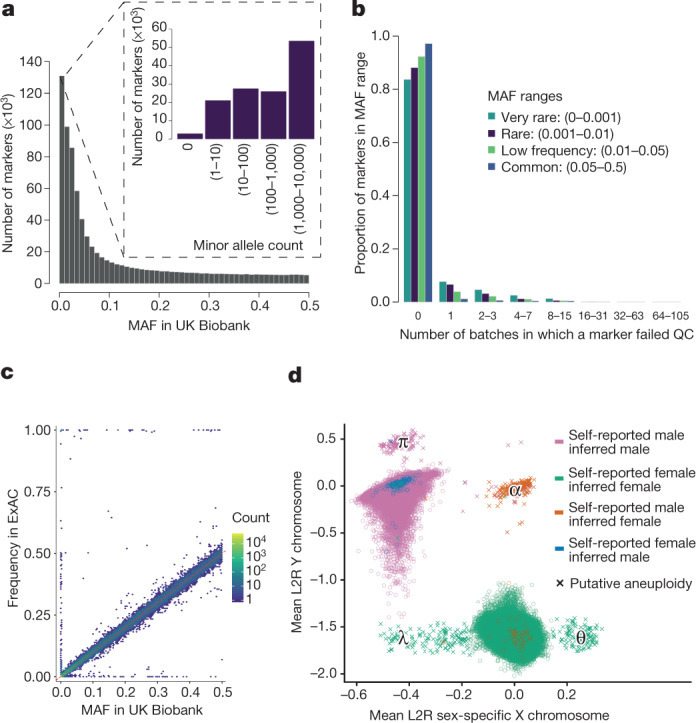
Summary of genotype data quality and content. All plots show properties of the UK Biobank genotype data after applying quality control. **a**, MAF distribution based on all samples (805,426 markers). The inset shows rare markers only (MAF < 0.01). **b**, The distribution of the number of batch-level quality control (QC) tests that a marker fails (see Methods). For each of four MAF ranges, we show the fraction of markers that fail the specified number of batches. **c**, Comparison of MAF in UK Biobank with the frequency of the same allele in ExAC, among the European-ancestry participants within each study (Supplementary Information). This analysis used 91,298 overlapping markers. Each hexagonal bin is coloured according to the number of markers falling in that bin (log_10_ scale). The dashed red line shows *x* = *y*. The markers with very different allele frequencies seen on the top, bottom and left-hand sides of the plot comprise approximately 300 markers. This is 0.3% of all markers in the comparison (see Supplementary Information for discussion). **d**, Mean log_2_ ratios (L_2_R) on X and Y chromosomes for each sample, indicating probable sex chromosome aneuploidy (see Methods). There are 652 samples with a probable sex chromosome aneuploidy (indicated by crosses). Locations of clusters of individuals with different putative karyotypes are indicated by Greek symbols: λ = X0 (or mosaic XX/X0), θ = XXX, α = XXY, and π = XYY. Counts of individuals in these regions are given in Supplementary Table 2. The colours indicate different combinations of self-reported sex, and sex inferred by Affymetrix (from the genetic data). For almost all samples (99.9%), the self-reported and the inferred sex are the same, but for a small number of samples (378) they do not match (see Supplementary Information for discussion).

Mismatches between self-reported sex of each individual, and sex inferred from the relative intensity of markers on the Y and X chromosomes^16^, can be used as a way to detect possible sample mishandling or other types of clerical error. In a dataset of this size, some such mismatches would be expected due to transgender or intersex individuals, or instances of rare genetic variation, such as sex-chromosome aneuploidies^17^. Using information in the measured intensities of chromosomes X and Y (see Methods), we identified a set of 652 (0.134%) individuals with sex chromosome karyotypes that were putatively different from XY or XX (Fig. 2d, Supplementary Table 2).

The application of our quality control pipeline resulted in the released dataset of 488,377 samples and 805,426 markers from both arrays with the properties shown in Fig. 2a–c. A set of 588 pairs of experimental duplicates show very high genotype concordance, with mean 99.87% and minimum 99.39% of genotypes identical (Supplementary Fig. 13). We compared allele frequencies among UK Biobank participants with European ancestry to those estimated from an independent source, the Exome Aggregation Consortium (ExAC) database^18^ at a set of 91,298 overlapping markers. We do not expect allele frequencies in the two studies to match exactly owing to subtle differences in the ancestral backgrounds of the individuals in each study, as well as differences in the sensitivity and specificity of the two technologies (exome sequencing and genotyping arrays). A small number of markers (around 300) have very different allele frequencies (see Supplementary Information section 2.4). This could be due to non-working probesets on the UK Biobank arrays or possibly annotation error on the UK Biobank arrays or in ExAC, or mapping errors in the sequence data in regions of more complex variation. Despite this, overall the allele frequencies are encouragingly similar (*r*^2^ = 0.93) (Fig. 2c; Supplementary Fig. 4).

**Extended Data Fig. 2:**
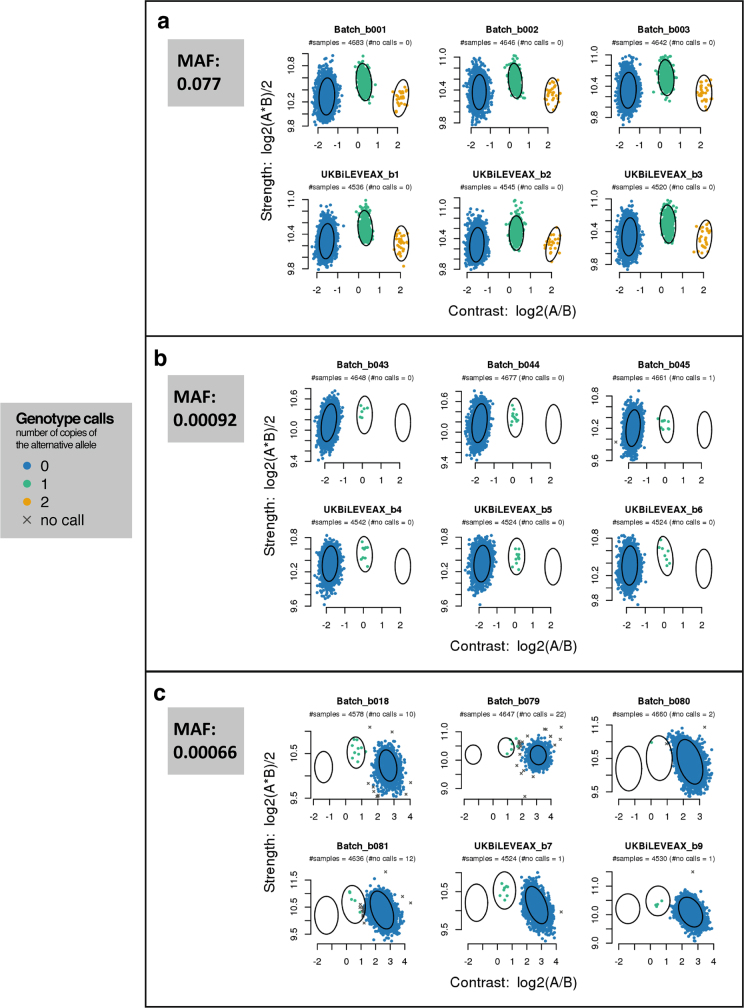
Examples of intensity data and genotype calls for markers of different allele frequencies. Each sub-figure shows intensity data for a single marker within six different batches. Batches labelled with the prefix ‘UKBiLEVEAX’ contain only samples typed using the UK BiLEVE Axiom array, and those with the prefix ‘batch’ contain only samples typed using the UK Biobank Axiom array. Each point represents one sample and is coloured according to the inferred genotype at the marker. The *x* and *y* axes are transformations of the intensities for probe sets targeting each of the alleles ‘A’ and ‘B’ (see Supplementary Information for definition of probe set). The ellipses indicate the location and shape of the posterior probability distribution (two-dimensional multivariate normal) of the transformed intensities for the three genotypes in the stated batch. That is, each ellipse is drawn such that it contains 85% of the probability density. See Affymetrix *Axiom Genotyping Solution Data Analysis Guide*^16^ for more details of Affymetrix genotype calling. The MAF of each of the markers is computed using all samples in the released UK Biobank genotype data. **a**, A marker with a MAF of 0.077 with well-separated genotype clusters. **b**, Intensities for a marker with a MAF of 0.00092 with well-separated genotype clusters. As would be expected under Hardy–Weinberg equilibrium, there are no instances of samples with the minor homozygote genotype. **c**, Intensities for a marker with a MAF of 0.00066, and in which the heterozygote cluster is not well separated from the large major homozygote cluster in some batches, making it more difficult to call the heterozygous genotypes confidently.

More than 110,000 rare markers (MAF < 0.01 in UK Biobank) were included on the two arrays used for the UK Biobank cohort^2^. Variants occurring at very low frequencies present a particular challenge for genotype calling using array technology. It can be challenging to distinguish a sample that genuinely has the minor allele, from one in which the intensities are in the tails of the distribution of those in the major homozygote cluster (Extended Data Fig. 2). A larger fraction of rare markers fail quality control tests compared to low frequency and common markers, but 84% still pass in all batches (Fig. 2b). We recommend researchers visually inspect cluster plots, similar to Supplementary Fig. 2, for markers of interest using a utility such as Evoker (https://github.com/wtsi-medical-genomics/evoker), especially for rare markers.

## Ancestral diversity and cryptic relatedness

**Fig. 3:**
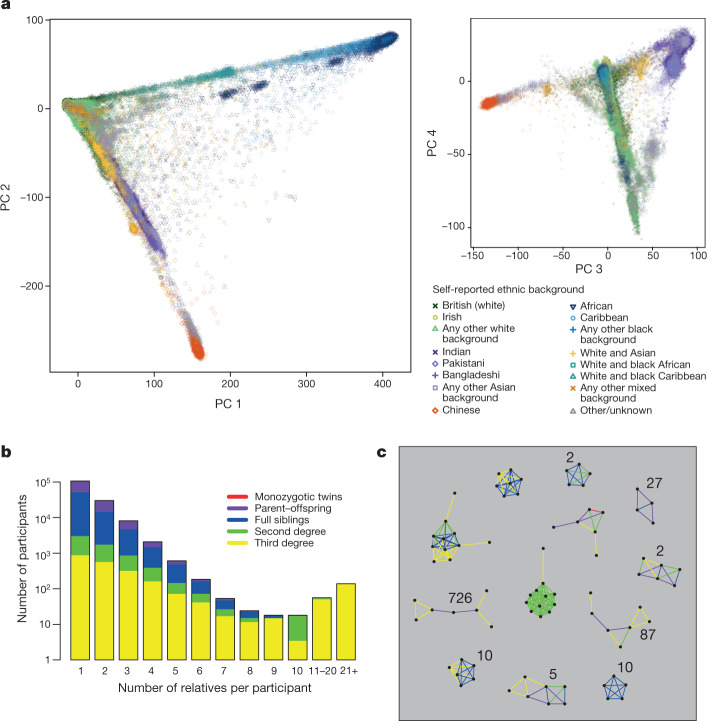
Ancestral diversity and familial relatedness. **a**, Each point represents a UK Biobank participant (*n* = 488,377 samples) and is placed according to their principal component (PC) scores in each of the top four principal components. Colours and shapes indicate the self-reported ethnic background of each individual. See Extended Data Table 3 for proportions in each category. **b**, Distribution of the number of relatives that participants have in the UK Biobank cohort. The height of each bar shows the count of participants (log_10_ scale) with the stated number of relatives. The colours indicate the proportions of each relatedness class within a bar. **c**, Examples of family groups within the UK Biobank cohort. Points represent participants, and coloured lines between points indicate their inferred relationship (for example, blue lines join full siblings). The integers show the total number of family networks in the cohort (if more than one) with that same configuration, ignoring third-degree pairs.

**Extended Data Fig. 3:**
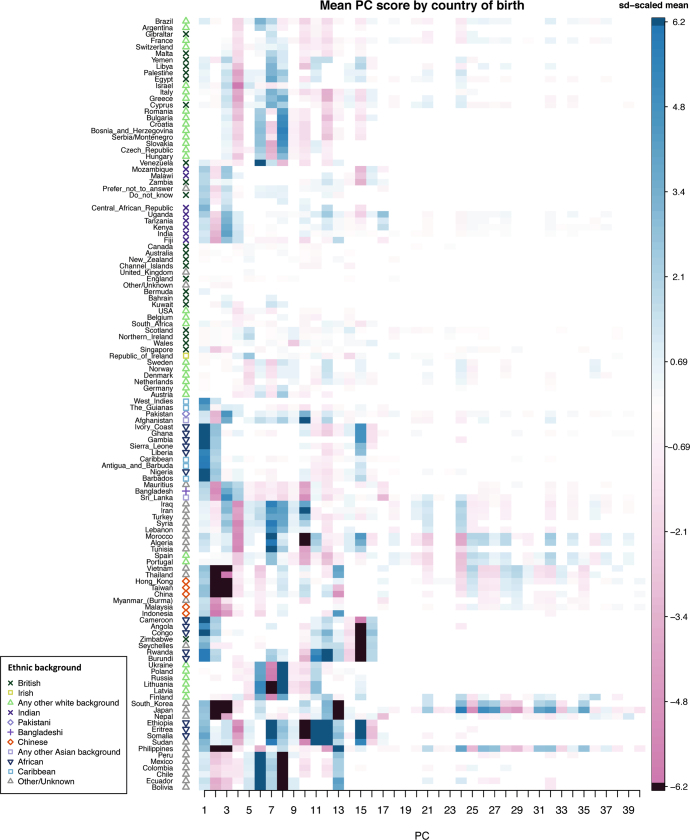
Mean principal component scores for each self-reported country of birth. Each column shows one principal component and each element is the mean principal component score for individuals born in the labelled country, scaled by the standard deviation of the scores for that principal component. Elements in each column are only coloured if the country has a non-zero coefficient (*P* < 10^−5^; two-sided *t*-test) in a linear model with country of birth as predictor and principal component scores as outcome (*n* = 487,848 samples). Countries (rows) have been ordered using hierarchical clustering (‘hclust’ function in R). The symbols next to each country label indicate the most common ethnic background category among the participants born in that country. For example, the most common self-reported ethnic background of participants born in Sri Lanka is ‘Any other Asian background’. Countries with fewer than 20 individuals born there were excluded from this analysis.

The genotype data provide a unique opportunity to study the diverse ancestral origins (Extended Data Table 3) of UK Biobank participants. Accounting for the ancestral background is essential both for epidemiological studies and genetic analyses, such as GWAS^19^. We used PCA to measure population structure within the UK Biobank cohort (see Methods). Figure 3a shows results for the first four principal components plotted in consecutive pairs (see also Extended Data Fig. 3 and Supplementary Figs. 6, 7). As expected, individuals with similar principal component scores have similar self-reported ethnic backgrounds. For example, the first two principal components separate out individuals with sub-Saharan African ancestry, European ancestry and east Asian ancestry. Individuals who self-report as mixed ethnicity tend to fall on a continuum between their constituent groups. Further principal components capture population structure at sub-continental geographic scales (Extended Data Fig. 3). Our PCA revealed population structure within the most common ethnic background category (88.26%), ‘British’ within the broader-level group ‘white’ (Supplementary Fig. 8). We used a combination of self-reported ethnic background and PCA results to provide researchers with a list of 409,728 individuals (84%) who have very similar ancestral backgrounds relative to the full cohort (see Methods).

Close relationships (for example, siblings) among UK Biobank participants were not recorded during the collection of other phenotypic information. This information can be important for epidemiological analyses^20^, as well as in GWAS^21^. We used the genetic data to identify related individuals by estimating kinship coefficients for all pairs of samples, and report coefficients for pairs of relatives who we infer to be third-degree relatives or closer (see Methods). A total of 147,731 UK Biobank participants (30.3%) are inferred to be related (third degree or closer) to at least one other person in the cohort, and form a total of 107,162 related pairs (Extended Data Table 5). This is a surprisingly large number, and it is not driven solely by an excess of third-degree relatives. For example, the number of sibling pairs (22,666) is roughly twice as many as would theoretically be expected in a random sample (of this size) of the eligible UK population, after taking into account typical family sizes (Supplementary Table 4). The larger than expected number of related pairs could be explained by sampling bias due to, for example, an individual being more likely to agree to participate because a family member was also involved. Furthermore, if, as seems plausible, related individuals cluster geographically rather than being randomly located across the UK, the recruitment strategies of the UK Biobank assessment centres^22^ will naturally tend to oversample related individuals.

Pairs of related individuals within the UK Biobank cohort form networks of related individuals. In most cases, these are of size two, but there are also many groups of size three or larger in the cohort (Fig. 3b), even when restricting to second-degree relatives or closer relative pairs. By considering the relationship types and the age and sex of the individuals within each family group, we identified 1,066 sets of trios (two parents and an offspring), which comprise 1,029 unique sets of parents and 37 quartets (two parents and two children).

There are 172 family groups with 5 or more individuals that are second-degree relatives or closer (Fig. 3c). One such group has 11 individuals who are all second-degree relatives of each other (half-siblings, grandparent/grandchild, or avuncular). Because all of the 55 pairs are second-degree relatives, at least 10 of them must be half-siblings with the same shared parent (see Supplementary Material). We confirmed that the shared parent must be their father because they do not all carry the same mitochondrial alleles, and the males all have the same Y chromosome alleles (data not shown).

## Haplotype estimation and genotype imputation

We estimated haplotypes for the full cohort (pre-phasing), followed by haploid imputation^23^. For the pre-phasing step, we only used markers present on both the UK BiLEVE and UK Biobank Axiom arrays. We removed markers that failed quality control in more than one batch, had a greater than 5% overall missing rate, and had a MAF of less than 0.0001. We removed samples that were identified as outliers for heterozygosity and missing rate. These filters resulted in a dataset with 670,739 autosomal markers in 487,442 samples. Phasing on the autosomes was carried out using SHAPEIT3^24^ (see Methods and https://jmarchini.org/software/). The 1000 Genomes phase 3 dataset^25^ was used as a reference panel, predominantly to help with the phasing of samples with non-European ancestry. In a separate experiment that leveraged phase inferred from mother–father–child trios, we estimated a median phasing switch error rate of 0.229% (see Methods).

We used the Haplotype Reference Consortium (HRC)^26^ data as the main imputation reference panel because it consisted of the largest available set (64,976) of broadly European haplotypes at 39,235,157 SNPs. Supplementary Fig. 15 shows the results of a separate imputation experiment that shows that the HRC panel produces better imputation performance than the UK10K panel, especially at lower allele frequencies, and that the UK Biobank Axiom array performs favourably compared to other commercially available arrays.

We also imputed the UK Biobank using the merged UK10K and 1000 Genomes phase 3 reference panels^27^, which has 87,696,888 bi-allelic markers. We combined this imputed data with that from the HRC panel, using the HRC imputation when a SNP was present in both panels. Imputation was carried out with the IMPUTE4 program (https://jmarchini.org/software/), which is a re-coded version of the haploid imputation functionality implemented in IMPUTE2^23^ (see Methods). The result of the imputation process is a dataset with 93,095,623 autosomal SNPs, short indels and large structural variants in 487,442 individuals. We imputed an additional 3,963,705 markers on the X chromosome (Methods). The SNP database (dbSNP) reference SNP (rs) IDs were assigned to as many markers as possible using reference SNP ID lists available from the UCSC genome annotation database for the GRCh37 assembly of the human genome (http://hgdownload.cse.ucsc.edu/goldenpath/hg19/database/).

**Extended Data Fig. 4:**
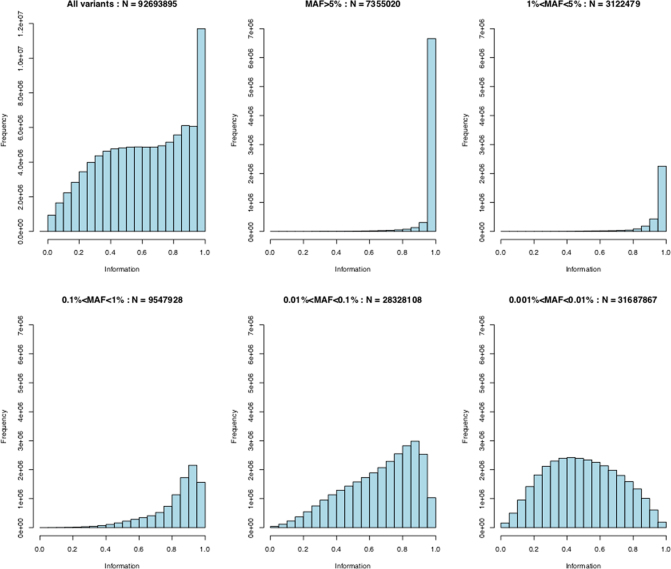
Distribution of information scores at autosomal markers in the imputed dataset. The top left graph shows the full distribution of the information scores. The remaining panels show distributions in tranches of MAF; MAF > 5%, 1% ≤ MAF < 5%, 0.1% ≤ MAF < 1%, 0.01% ≤ MAF < 0.1% and 0.001% ≤ MAF < 0.01%.

Extended Data Fig. 4 shows the distribution of information scores on all markers in the imputed dataset. An information score of *α* in a sample of *M* individuals indicates that the amount of data at the imputed marker is approximately equivalent to a set of perfectly observed genotype data in a sample size of *αM*. The figure illustrates that most markers above 0.1% frequency have high information scores. Previous GWAS have tended to use a filter on information around 0.3 that roughly corresponds to an effective sample size of approximately 150,000. Thus, it may be possible to reduce the information score threshold and still obtain good power to detect associations.

We developed a new BGEN file format (v1.2; http://www.well.ox.ac.uk/~gav/bgen_format/bgen_format.html) and software library (BGEN; https://bitbucket.org/gavinband/bgen) to provide improved data compression, the ability to store phased haplotype data and random access to the data via use of a separate index file. Using this new format, the full imputed files require 2.1 Tb of file space. A new program (BGENIE; https://jmarchini.org/software) was built using the BGEN library to carry out fast multi-trait GWAS and phenome-wide association studies^28^ (see Supplementary Information).

## Imputation of classical HLA alleles

The major histocompatibility complex (MHC) on chromosome six is the most polymorphic region of the human genome and contains the largest number of genetic associations to common diseases^29^. We imputed HLA types at two-field (also known as four-digit) resolution for 11 classical HLA genes (*HLA*-*A*, *HLA*-*B*, *HLA*-*C*, *HLA*-*DRB1*, *HLA*-*DRB3*, *HLA*-*DRB4*, *HLA*-*DRB5*, *HLA-DQA1*, *HLA*-*DQB1*, *HLA*-*DPA1* and *HLA*-*DPB1*) using the HLA*IMP:02 algorithm with a multi-population reference panel (Supplementary Tables 5 and 6)^30^ and validated the accuracy using a cross-validation experiment. In a typical use, case accuracy was estimated at better than 96% across all loci (see Methods and Supplementary Tables 7, 8).

To demonstrate the utility of the HLA imputation, we performed association tests for diseases known to have HLA associations. We analysed 409,724 individuals in the white British ancestry subset (see Methods) and focused on 11 self-reported immune-mediated diseases with known HLA associations. For each disease in our analysis, we identified the HLA allele with the strongest evidence of association. In all cases these were consistent with previous reports (see Methods and Supplementary Table 9). We further replicated independent HLA associations in a single disease study of multiple sclerosis (MS) susceptibility by the International Multiple Sclerosis Genetics Consortium (IMSGC)^31^. Here we observed evidence of association and effect size estimates for HLA alleles that are concordant in direction and relative magnitude with those found in the IMSGC study, although in 11 out of 14 cases this was closer to 1, consistent with regression dilution bias arising from a low rate of phenotypic error (Table 1).

**Table 1:** Association between HLA alleles and MS in UK Biobank and IMSGC cohort

HLA allele	Test	UK Biobank		IMSGC	
		OR (95% CI)	*P* value	OR (95% CI)	*P* value
HLA-DRB1*15:01	Additive effect	3.16 (2.81–3.54)	2.58 × 10^−85^	3.92 (3.74–4.12)	<1 × 10^−600^
	Homozygote correction	0.67 (0.52–0.87)	2.32 × 10^−3^	0.54 (0.47–0.61)	8.50 × 10^−22^
HLA-A*02:01	Additive effect	0.69 (0.62–0.78)	2.30 × 10^−10^	0.67 (0.64–0.70)	7.80 × 10^−70^
	Homozygote correction	1.20 (0.89–1.62)	2.41 × 10^−1^	1.26 (1.13–1.41)	3.30 × 10^−05^
HLA-DRB1*03:01	Additive effect	1.21 (1.06–1.37)	3.39 × 10^−3^	1.16 (1.10–1.22)	3.50 × 10^−08^
	Homozygote correction	2.12 (1.53–2.94)	6.84 × 10^−6^	2.58 (2.19–3.03)	1.30 × 10^−30^
HLA-DRB1*13:03	Additive effect	2.10 (1.54–2.85)	2.36 × 10^−6^	2.62 (2.32–2.96)	6.20 × 10^−55^
HLA-DRB1*08:01	Additive effect	1.56 (1.21–2.01)	6.13 × 10^−4^	1.55 (1.42–1.69)	1.00 × 10^−23^
HLA-B*44:02	Additive effect	0.86 (0.74–0.98)	2.94 × 10^−2^	0.78 (0.74–0.83)	4.70 × 10^−17^
HLA-B*38:01	Additive effect	0.29 (0.13–0.65)	2.55 × 10^−3^	0.48 (0.42–0.56)	8.00 × 10^−23^
HLA-B*55:01	Additive effect	0.99 (0.75–1.31)	9.47 × 10^−1^	0.63 (0.55–0.73)	6.90 × 10^−11^
HLA-DQA1*01:01	Additive effect in the presence of HLA-DRB1*15:01	0.71 (0.56–0.90)	5.33 × 10^−3^	0.65 (0.59–0.72)	1.30 × 10^−17^
HLA-DQB1*03:02	Dominant effect	1.07 (0.92–1.25)	3.71 × 10^−1^	1.30 (1.23–1.37)	1.80 × 10^−22^
HLA-DQB1*03:01	Allelic interaction with HLA-DQB1*03:02	0.8 (0.53–1.20)	2.81 × 10^−1^	0.60 (0.52–0.69)	7.10 × 10^−12^

Evidence for association between HLA alleles and MS in UK Biobank compared to the IMSGC cohort. The UK Biobank association tests involved 1,501 self-reported cases and 409,724 controls; the IMSGC cohort involved 17,465 cases and 30,385 controls^31^. Thus, the UK Biobank analysis had significantly lower power than the IMSGC analysis, which is reflected in the reported *P* values and larger confidence interval (CI) estimates for the odds ratios (OR). Effect sizes for the UK Biobank were estimated jointly using the logistic regression model of the MHC reported by the IMSGC (with the exception of the two SNPs rs9277565 and rs2229029). As in the IMSGC analysis, the homozygote correction test indicates a departure from additivity. That is, if the odds ratio is <1 then the homozygous effect is smaller than under the additivity assumption and bigger if it is >1. Reported *P* values were calculated using the Wald test.

## GWAS for standing height

To assess the potential of the directly genotyped and imputed data, we conducted a GWAS for standing height using 343,321 unrelated, European-ancestry UK Biobank participants (see Methods). We compared our results to a non-overlapping meta-analysis of 253,288 individuals of European ancestry carried out by the Genetic Investigation of Anthropometric Traits (GIANT) Consortium^32^.

**Fig. 4:**
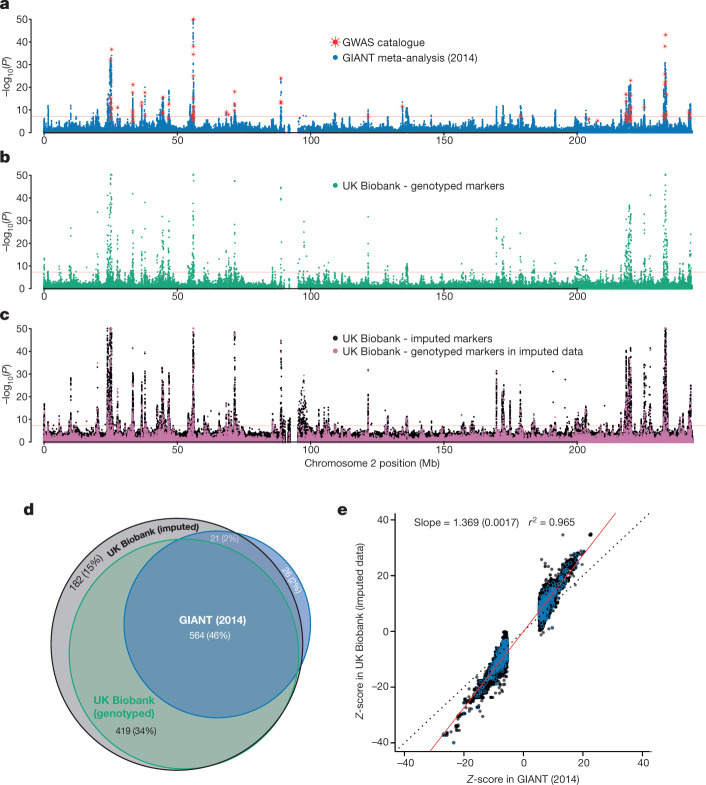
Association statistics for human height. Results (*P* values) of association tests between human height and genotypes using three different sets of data for chromosome 2. In **a**–**c**, *P* values are shown on the −log_10_ scale, capped at 50 for visual clarity and uncorrected for multiple comparisons. Markers with −log_10_(*P*) > 50 are plotted at 50 on the *y* axis and shown as triangles rather than dots. Horizontal red lines denote *P* = 5 × 10^−8^. **a**, Results for published meta-analysis by GIANT^32^ (*n* = 253,288), with NCBI GWAS catalogue markers superimposed in red (plotted at the reported *P* values). **b**, Association statistics (from linear mixed model, see Methods) for UK Biobank markers in the genotype data (*n* = 343,321). **c**, Association statistics (from linear mixed model, see Methods) for UK Biobank markers in the imputed data (*n* = 343,321). Points coloured pink indicate genotyped markers that were used in pre-phasing and imputation. This means that most of the data at each of these markers comes from the genotyping assay. Black points (the vast majority, ~8 million) indicate fully imputed markers. **d**, Venn diagram of the results of counting the number of 1-Mb windows with at least one locus with *P* < 5 × 10^−8^ in the GIANT, UK Biobank genotyped and UK Biobank imputed datasets (see Methods). Percentages in brackets are the proportion of the union of such windows across all three data sources (1,215). There were only three windows contained in UK Biobank genotyped data and not the imputed data. **e**, Comparison of *Z*-scor**e**s in UK Biobank (*y* axis) and GIANT (*x* axis). *Z*-scores were calculated as effect size divided by standard error, but only for markers with *P* < 5 × 10^−8^ in GIANT, for a set of 575 associated regions, which we also used for the credible set analysis (see Methods). The marker with the smallest *P* value (in GIANT) within each region is highlighted with blue circles. The black dotted line shows *x* = *y*, and the red solid line shows the linear regression line estimated on these data. The standard error of the regression coefficient is shown in brackets. Pearson’s correlation was used to calculate the *r*^2^ value.

**Extended Data Fig. 5:**
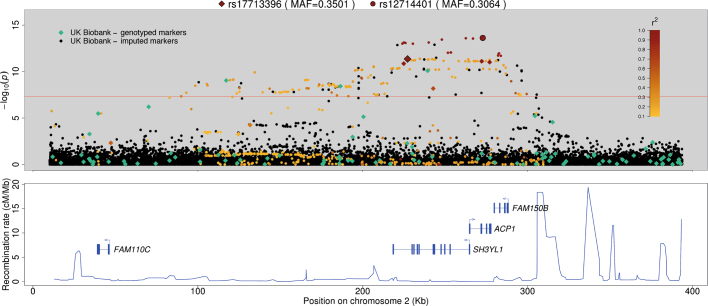
Example region of association in standing height GWAS. GWAS association statistics (*P* values) for standing height focusing on a ~3-Mb region of chromosome 2 that did not reach genome-wide significance in the GIANT (2014) meta-analysis, but did in UK Biobank (linear mixed model; see Methods). The *P* values shown are not adjusted for multiple testing. Markers genotyped in the UK Biobank are shown as diamonds, and imputed markers as circles. The two markers with the smallest *P* value for each of the genotyped data and imputed data are enlarged and highlighted with black outlines, and other UK Biobank markers are coloured according to their correlation (*r*^2^) with one of these two. That is, genotyped markers with the leading genotyped marker (rs17713396), and imputed markers with the leading imputed marker (rs12714401). Markers with *r*^2^ values of less than 0.1 are shown as black or green.

Reassuringly, the pattern of association signals is similar in both the UK Biobank and GIANT results (Fig. 4a–c), and the *Z*-scores at associated markers are highly correlated (*r*^2^ = 0.965; Fig. 4e). The gain in power in the UK Biobank cohort is clear, with many loci reaching genome-wide significance (*P* < 5 × 10^−8^) in the UK Biobank but not in the GIANT study (Fig. 4d, Supplementary Fig. 16); and *Z*-scores for associated markers are systematically higher in UK Biobank (regression slope = 1.369, Fig. 4e). Regions of association in the UK Biobank show patterns of signal expected given the linkage disequilibrium structure and recombination rates in the region (see Extended Data Fig. 5 for an example).

**Extended Data Fig. 6:**
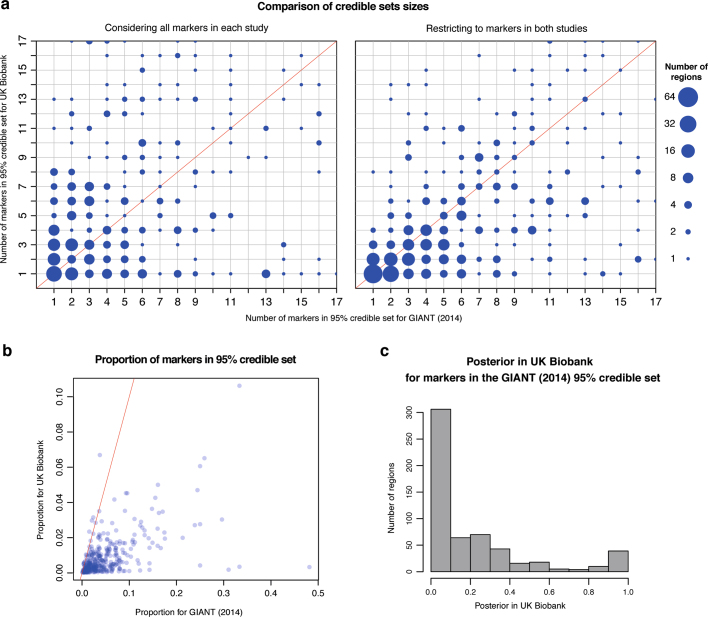
Comparison of fine-mapping in GIANT (2014) and UK Biobank imputed data. Here we summarize results of our credible set analysis in GIANT (2014) and UK Biobank for 575 genomics regions associated with standing height in both studies (see Methods). A red solid line on a plot indicates where *x* = *y*. **a**, Both plots compare the number of markers in the 95% credible sets in which the size is less than 18 markers in both studies (363 regions in the left-hand plot; 445 in the right-hand plot). **b**, **c**, Both plots are from the analysis considering all markers in each study. In **b** we show, for each region, the proportion of markers used in the analysis for a given study that are in the 95% credible set for that study. The plot contains the same 363 regions as shown in the left-hand plot in **a**. In **c** we summarize, for all 575 regions, how much weight our UK Biobank analysis placed on markers that our analysis of GIANT (2014) indicated were important.

To assess the effectiveness of UK Biobank genomic data for fine-mapping within associated loci, we computed 95% credible sets^33^ for 575 regions that contain at least one genome-wide significant marker (*P* < 5 × 10^−8^) in both GIANT and the UK Biobank imputed data (see Methods). The number of markers we analysed in the UK Biobank (768,502) is considerably more than in GIANT (106,263), and this affects the resolution of any given associated region (Extended Data Fig. 6a). When considering all markers, the size of the credible set in UK Biobank is usually larger (median size = 8) than in GIANT (median size = 6), but the proportion of SNPs in the credible set of each region (Extended Data Fig. 6b) is generally smaller in UK Biobank (median proportion = 0.010) than in GIANT (median proportion = 0.047). By restricting to the markers in both studies (105,421) we find that the size of the 95% credible set is generally smaller in UK Biobank (median size = 4) than GIANT (median size = 6). The number of 95% credible sets that contain just 1 marker is 123 in UK Biobank and 76 in GIANT.

## Conclusion

The interim release of the genetic data on approximately 150,000 participants in UK Biobank has already facilitated many papers exploring the links between human genetic variation and disease, and their connection with a wide range of environmental and lifestyle factors. The UK Biobank continues to grow with the addition of further phenotypic information and as researchers return the results of their analyses for UK Biobank to share. Online resources are being developed for sharing the results of analyses using UK Biobank data, including the release of GWAS results for thousands of phenotypes (http://www.nealelab.is/uk-biobank) and the Oxford Brain Imaging Genetics server^28^ (http://big.stats.ox.ac.uk/). We anticipate that the availability of the full genetic data for UK Biobank will result in a further step change in this productive research cycle. The UK Biobank is a powerful example of the immense value that can be achieved from large population scale studies that combine genetics with extensive and deep phenotyping and linkage to health records coupled with a strong data sharing policy. It is likely to herald a new era in which these and related resources drive and enhance understanding of human biology and disease.

## Methods

### Data collection, sample retrieval, DNA extraction and genotype calling

Ethics approval for the UK Biobank study was obtained from the North West Centre for Research Ethics Committee (11/NW/0382). Blood samples were collected from participants on their visit to a UK Biobank assessment centre and the samples are stored at the UK Biobank facility in Stockport, UK^7^. Over a period of 18 months samples were retrieved, DNA was extracted, and 96-well plates of 94 × 50-μl aliquots were shipped to Affymetrix Research Services Laboratory for genotyping. Special attention was paid in the automated sample retrieval process at UK Biobank to ensure that experimental units such as plates or timing of extraction did not correlate systematically with baseline phenotypes such as age, sex, and ethnic background, or the time and location of sample collection. Full details of the UK Biobank sample retrieval and DNA extraction process were described previously^34^.

On receipt of DNA samples, Affymetrix processed samples on the GeneTitan Multi-Channel (MC) Instrument in 96-well plates containing 94 UK Biobank samples and two control samples from the 1000 Genomes Project^25^. Genotypes were then called from the array intensity data, in units called ‘batches’ which consist of multiple plates. Across the entire cohort, there were 106 batches of 4,700 UK Biobank samples each (Supplementary Information, Supplementary Table 12). Following the earlier interim data release, Affymetrix developed a custom genotype calling pipeline that is optimized for biobank-scale genotyping experiments, which takes advantage of the multiple-batch design^35^. This pipeline was applied to all samples, including the 150,000 samples that were part of the interim data release. Consequently, some of the genotype calls for these samples may differ between the interim data release and this final data release (see below).

Routine quality checks were carried out during the process of sample retrieval, DNA extraction^36^, and genotype calling^37^. Any sample that did not pass these checks was excluded from the resulting genotype calls. The custom-designed arrays contain a number of markers that had not been previously typed using Affymetrix genotype array technology. As such, Affymetrix also applied a series of checks to determine whether the genotyping assay for a given marker was successful, either within a single batch, or across all samples. Where these newly attempted assays were not successful, Affymetrix excluded the markers from the data delivery (see Supplementary Information for details).

### Marker-based quality control

We identified poor quality markers using statistical tests designed primarily to check for consistency of genotype calling across experimental factors. Specifically we tested for batch effects, plate effects, departures from Hardy–Weinberg equilibrium, sex effects, array effects, and discordance across control replicates. See Supplementary Information for the details of each test, and Supplementary Fig. 3 for examples of affected markers. For markers that failed at least one test in a given batch, we set the genotype calls in that batch to missing. We also provide a flag in the data release that indicates whether the calls for a marker have been set to missing in a given batch. If there was evidence that a marker was not reliable across all batches, we excluded the marker from the data altogether. To attenuate population structure effects, we applied all marker-based quality control tests using a subset of 463,844 individuals with estimated European ancestry. We identified these individuals from the genotype data before conducting any quality control by projecting all the UK Biobank samples on to the two major principal components of four 1000 Genomes populations (CEU, YRI, CHB and JPT)^25^. We then selected samples with principal component scores falling in the neighbourhood of the CEU cluster (Supplementary Information).

### Sample-based quality control

We identified poor quality samples using the metrics of missing rate and heterozygosity computed using a set of 605,876 high quality autosomal markers that were typed on both arrays (see Supplementary Information for criteria). Extreme values in one or both of these metrics can be indicators of poor sample quality due to, for example, DNA contamination^15^. The heterozygosity of a sample—the fraction of non-missing markers that are called heterozygous—can also be sensitive to natural phenomena, including population structure, recent admixture and parental consanguinity. We took extra measures to avoid misclassifying good quality samples because of these effects. For example, we adjusted heterozygosity for population structure by fitting a linear regression model with the first six principal components in a PCA as predictors (Extended Data Fig. 1). Using this adjustment we identified 968 samples with unusually high heterozygosity or >5% missing rate (Supplementary Information). A list of these samples is provided as part of the data release.

We also conducted quality control specific to the sex chromosomes using a set of 15,766 high quality markers on the X and Y chromosomes. Affymetrix infers the sex of each individual based on the relative intensity of markers on the Y and X chromosomes^16^. Sex is also reported by participants, and mismatches between these sources can be used as a way to detect sample mishandling or other kinds of clerical error. However, in a dataset of this size, some such mismatches would be expected due to transgender individuals, or instances of real (but rare) genetic variation, such as sex-chromosome aneuploidies^17^. Affymetrix genotype calling on the X and Y chromosomes allows only haploid or diploid genotype calls, depending on the inferred sex^16^. Therefore, cases of full or mosaic sex chromosome aneuploidies may result in compromised genotype calls on all, or parts of, the sex chromosomes (but not affect the autosomes). For example, individuals with karyotype XXY will probably have poorer quality genotype calls on the pseudo-autosomal region (PAR) of the X chromosome, as they are effectively triploid in this region. Using information in the measured intensities of chromosomes X and Y, we identified a set of 652 (0.134%) individuals with sex chromosome karyotypes putatively different from XY or XX (Fig. 2d, Supplementary Table 2). The list of samples is provided as part of the data release. Researchers wanting to identify sex mismatches should compare the self-reported sex and inferred sex data fields.

We did not remove samples from the data as a result of any of the above analyses, but rather provide the information as part of the data release. However, we excluded a small number of samples (835 in total) that we identified as sample duplicates (as opposed to identical twins, see Supplementary Information) or were probably involved in sample mishandling in the laboratory (~10), as well as participants who asked to be withdrawn from the project before the data release.

### Comparison of interim and final release data

Subsequent to the interim release of genotypes (May 2015) for approximately 150,000 UK Biobank participants improvements were made to the genotype calling algorithm^35^ and quality control procedures. We therefore expect to observe some changes in the genotype calls and missing data profile of samples included in both the interim data release and this final data release. Discordance among non-missing markers is very low (mean 6.7 × 10^−5^; Supplementary Fig. 1); and for each sample there are 24,500 genotype calls (on average) that were missing in the interim data, but which have non-missing calls in this release. This is much smaller in the reverse direction, with 500 calls, on average, missing in this release but not missing in the interim data, so there is an average net gain of 24,000 genotype calls per sample.

### Principal component analysis

We computed principal components using an algorithm (fastPCA^38^) that performs well on datasets with hundreds of thousands of samples by approximating only the top *n* principal components that explain the most variation, in which *n* is specified in advance. We computed the top 40 principal components using a set of 407,219 unrelated, high quality samples and 147,604 high quality markers pruned to minimise linkage disequilibrium^39^. We then computed the corresponding principal component-loadings and projected all samples onto the principal components, thus forming a set of principal component scores for all samples in the cohort (Supplementary Information).

### White British ancestry subset

Researchers may want to only analyse a set of individuals with relatively homogeneous ancestry to reduce the risk of confounding due to differences in ancestral background. Although the UK Biobank cohort includes a large number of participants from a wide range of ethnic backgrounds, such analysis is feasible without compromising too much in sample size because most participants in the UK Biobank cohort report their ethnic background as ‘British’, within the broader-level group ‘white’ (88.26%). Our PCA revealed population structure even within this category (Supplementary Fig. 8), so we used a combination of self-reported ethnic background and genetic information to identify a subset of 409,728 individuals (84%) who self-report as ‘British’ and who have very similar ancestral backgrounds based on results of the PCA (Supplementary Information). Fine-scale population structure is known to exist within the UK but methods for detecting such subtle structure^40^ available at the time of analysis are not feasible to apply at the scale of the UK Biobank. The white British ancestry subset may therefore still contain subtle structure present at sub-national scales.

### Kinship coefficient estimation

We used an estimator implemented in the software, KING^41^, as it is robust to population structure (that is, does not rely on accurate estimates of population allele frequencies) and it is implemented in an algorithm efficient enough to consider all pairs (~1.2 × 10^11^) in a practicable amount of time. As noted by the authors of KING, we found that recent admixture (for example, ‘mixed’ ancestral backgrounds) tended to inflate the estimate of the kinship coefficient, as the estimator assumes Hardy–Weinberg equilibrium among markers with the same underlying allele frequencies within an individual. We alleviated this effect by only using a subset of markers that are only weakly informative of ancestral background (Supplementary Information, Supplementary Fig. 12). We also excluded a small fraction of individuals (977) from the kinship estimation, as they had properties (for example, high missing rates) that would lead to unreliable kinship estimates (Supplementary Information). We called relationship classes for each related pair using the kinship coefficient and fraction of markers for which they share no alleles (IBS0). See Supplementary Information section S3.7 for details.

To ensure we were not overestimating the number of related pairs, we inferred related pairs (within a subset of the data) using a different inference method implemented in PLINK (‘-genome’ command; https://www.cog-genomics.org/plink2) and confirmed 100% of the twins, parent-offspring and sibling pairs, and 99.9% of pairs overall (Supplementary Information).

### Haplotype estimation

Haplotype estimation (phasing) was carried out using SHAPEIT3 in chunks of 15,000 markers, with an overlap of 250 markers between chunks. Each chunk used 4 cores per job and *S* = 200 copying states. Chunks were ligated using a modified version of the hapfuse program (https://bitbucket.org/wkretzsch/hapfuse/src).

We assessed the accuracy of the phasing in a separate experiment by taking advantage of mother-father-child trios that were identified in the UK Biobank cohort. This family information can be used to infer the phase of a large number of markers in the trio parents. These family-inferred haplotypes were used as a truth set, as is common in the phasing literature. The parents of each trio were removed from the dataset and then haplotypes were estimated across chromosome 20 in a single run of SHAPEIT3. This dataset consisted of 16,175 autosomal markers. The inferred haplotypes were then compared to the truth set using the switch error metric. Using a set of 696 trios with self-reported ethnic background ‘British’ (within the broader-level group ‘white’) and no other twins or first- or second-degree relatives in the UK Biobank dataset, we estimated a median switch error rate of 0.229%. We also used a subset of 397 of these trios that also had no third-degree relatives and obtained a median switch error rate of 0.234%. These error rates are similar to those produced by other phasing methods that can handle data at this scale^42,43^. Investigations on the effect of sample size on phasing performance and downstream imputation performance suggest that differences between methods will have negligible effect on genotype imputation and GWAS^42^.

### Imputation

To facilitate fast imputation of all 500,000 samples, we re-coded IMPUTE2^23^ to focus exclusively on the haploid imputation needed when samples have been pre-phased. This new version of the program is referred to as IMPUTE4 (see https://jmarchini.org/software/), but uses exactly the same hidden Markov model within IMPUTE2, and produces identical results to IMPUTE2 when run using all reference haplotypes as hidden states (data not shown). To reduce RAM usage and increase speed we use compact data structures that store the indices of haplotypes carrying the non-reference allele at variant sites in the reference panel. Not only is this data structure compact, but at each stage of the forward-backward algorithm it also allows the calculations involving the emission part of the hidden Markov model to sum only over just the subset of haplotypes that carrying the non-reference allele in an efficient way. A further increase in speed is obtained by only calculating the marginal copying probabilities at those sites common to the target and reference datasets, and then linearly interpolating these for SNPs in-between those sites that need to be imputed. Imputation was carried out in chunks of approximately 50,000 imputed markers with a 250 kb buffer region and on 5,000 samples per compute job. The combined processing time per sample for the whole genome was approximately 10 min.

### Haplotype estimation and genotype imputation on the X chromosome

For haplotype estimation on the X chromosome genotype data we applied the same filtering steps as the autosomal genotype data, with some additional filters. For both the sex-specific region and the pseudo-autosomal regions (PAR), samples were excluded which were identified as having a likely sex chromosome aneuploidy (see above). For the PAR, we additionally excluded samples with a missing rate of >5% among markers in the PAR. For the sex-specific region of chromosome X, this resulted in a dataset of 16,601 markers and 486,790 samples. For the PAR this resulted in a dataset of 1,239 markers and 486,476 samples. Haplotype estimation and genotype imputation was carried out on the two pseudo-autosomal regions and the non-pseudo autosomal region separately, and using the same methods and reference datasets used for the autosomes.

### HLA imputation and validation

For each individual we defined the HLA genotype at each locus as the pair of alleles with maximum posterior probability as reported by HLA*IMP:02. We performed association analysis (see, for example, ref. ^31^) for HLA alleles and each disease using logistic regression. The risk model (additive, dominant, recessive or general), as described previously^31^, was used to enable comparison of effect size estimates. For validation and further details, see Supplementary Information section S5. We repeated the analysis, setting genotypes with a maximum posterior probability of <0.7 to missing. No significant differences were observed compared to the full analysis (data not shown). As a negative control, we ran association analyses in the HLA region with imputed HLA alleles for type 2 diabetes (2,849 cases) and myocardial infarction (9,725 cases) in a total of 409,724 individuals and we found no significant associations (all *P* > 2.40 × 10^−4^, the Bonferroni corrected level of association) with any HLA alleles, which is consistent with the lack of associations in the HLA region in recent analyses of each phenotype^44,45^

We estimated the accuracy of the imputation process using fivefold cross-validation in the reference panel samples. For samples of European ancestry, the estimated four-digit accuracy for the maximum posterior probability genotype is above 93.9% for all 11 loci (Supplementary Table 7). This accuracy improved to above 96.1% for all 11 loci after restricting to HLA allelic variant calls with a posterior probability greater than 0.70. This resulted in call rates above 95.1% for all loci (Supplementary Table 8).

### GWAS for standing height

We conducted the GWAS for standing height using the directly genotyped and imputed data in the form that they are made available to researchers, but with a subset of samples. Specifically, we only included samples with all of the following properties: (i) imputation was carried out on them; (ii) in the white British ancestry subset (see above); and (iii) the inferred sex matches the self-reported sex. From this group we selected a set of 344,397 unrelated individuals (Supplementary Information). For standing height, a further 1,076 individuals were excluded owing to missing values for the phenotype, leaving a total of 343,321 for association testing.

We used the software BOLT-LMM (v2.2)^46^ to look for evidence of statistical association between each marker and standing height. We report association statistics based on a linear mixed model (BOLT-LMM-inf), with the following covariates: (i) array (UK BiLEVE Axiom Array or UK Biobank Axiom Array); (ii) sex (inferred); (iii) age when attended UK Biobank assessment centre; and (iv) principal components 1–20.

The principal components scores were computed using only individuals within the white British ancestry subset, but otherwise with the same method as described above. We conducted tests using the genotype and imputed data files separately.

### Example of association region in standing height GWAS

Extended Data Fig. 5 shows an example of an associated region on chromosome 2. Correlations (*r*^2^) between markers in this region show a pattern that is as expected in the context of linkage disequilibrium, and the local recombination rates. The stripe-like pattern of the association statistics is indicative of multiple mutations occurring on similar branches of the genealogical tree underlying the data, which are probably linked to varying degrees with the causal marker(s). The correlation between the most associated marker and all other markers in the region drops off sharply around the small peak in recombination^47^ to the right of the most significantly associated marker. Notably, this marker was imputed from the genotypes, which points to the success of the imputation in this study, and in general, to the value of imputing millions more markers. Human height is a highly polygenic trait, so provided an opportunity to examine many such regions of association, and other regions that we visually examined showed similar patterns.

### Comparison of GIANT and UK Biobank GWAS results

For Fig. 4d, e and the credible set analysis we used autosomal markers only, and filtered markers in each data source such that MAF > 0.001 (defined in the GWAS population), and Info score > 0.3 in the UK Biobank imputed data. There were 16,443,622 such markers in UK Biobank imputed data, 703,946 in the UK Biobank genotyped data, and 2,546,872 in GIANT.

For a given phenotype, the 95% credible set in a region of association is the smallest set of markers that together have 95% posterior probability of containing the marker causally associated with the phenotype. We found credible sets for standing height using the method described previously^33^ and summarize the results in Extended Data Fig. 6. It is important to note that this approach is based on a model in which there is exactly one causal marker in the region and genotypes for that marker are available in the data. Our results should therefore be considered as indicative of a more detailed analysis where, for example, the regions are first analysed to distinguish independent association signals.

In our analysis, we first defined a set of 575 non-overlapping regions associated with standing height using a procedure based on that used previously^15^ (see Supplementary Information). For each study, we carried out two separate analyses to find credible sets in these regions: (A) using all the markers in each study (768,502 in UK Biobank imputed data; 106,263 in GIANT); and (B) using only those markers in both studies (105,421).

For each marker in each study, we computed a Bayes factor in favour of association with standing height using the effect sizes and standard errors, and 0.2^2^ as the prior^33^ on the variance of the effect sizes. To ensure the effect sizes were on the same scale in both studies we scaled UK Biobank effect sizes and standard errors by the standard deviation of the residuals of the measured phenotype (standing height) after regressing out the covariates used in the GWAS. We then confirmed that the effect size estimates for overlapping markers were comparable between the two studies.

If there is exactly one causal marker in the region and genotypes for that marker are available in the data, then the posterior probability that a marker *i* drives the association signal in the region *r* is given by:

$${\pi }_{ir}=\frac{{{\rm{BF}}}_{ir}}{{{\rm{\Sigma }}}_{k}{{\rm{BF}}}_{kr}}$$

where BF_*kr*_ is the Bayes factor for marker *i* in the *r* region^33^. The 95% credible set for a region is found by going down the list of markers ordered from highest to lowest posterior probability and stopping when the cumulative posterior reaches 0.95.

We assessed the sensitivity of our results to the choice of prior by conducting the same analyses using a much smaller prior (0.02^2^) and much larger prior (20^2^). We found that overall the choice of prior had little effect on the results. Specifically for values we report in the main text, the median credible set sizes were unaffected in all analyses. For the larger prior, the number of single-marker credible sets was unaffected except for analysis B in UK Biobank (from 123 to 122), and the median proportion of markers in the credible set was unaffected in all analyses. For the smaller prior, the number of single-marker credible sets only changed for analysis A, going from 78 to 75 in GIANT, and 85 to 86 in UK Biobank, and the median proportion of markers in the credible set increased slightly in all analyses (maximum increase from 0.047 to 0.051).

### Code availability

Genotype imputation was carried out using IMPUTE4.0. Pre-compiled binaries for the latest version of IMPUTE4 are available at https://jmarchini.org/software/. This software is licensed free for use by researchers at academic institutions. The BGEN library source code is available at https://bitbucket.org/gavinband/bgen. BGENIE is built using this library. Pre-compiled binaries for the latest version of BGENIE are available at https://jmarchini.org/software/. This software is currently licensed free for use by researchers at academic institutions. Commercial organizations wishing to use IMPUTE4 or BGENIE must enquire about a licence from the University of Oxford.

### Reporting summary

Further information on research design is available in the Nature Research Reporting Summary linked to this paper.

## Online content

Any methods, additional references, Nature Research reporting summaries, source data, statements of data availability and associated accession codes are available at 10.1038/s41586-018-0579-z.

## Supplementary information

Supplementary Information This file contains Supplementary Material, including Supplementary Figures S1-S18 and Supplementary Tables S1-S13.

Reporting Summary

## Figures and Tables

**Extended Data Table 1:**
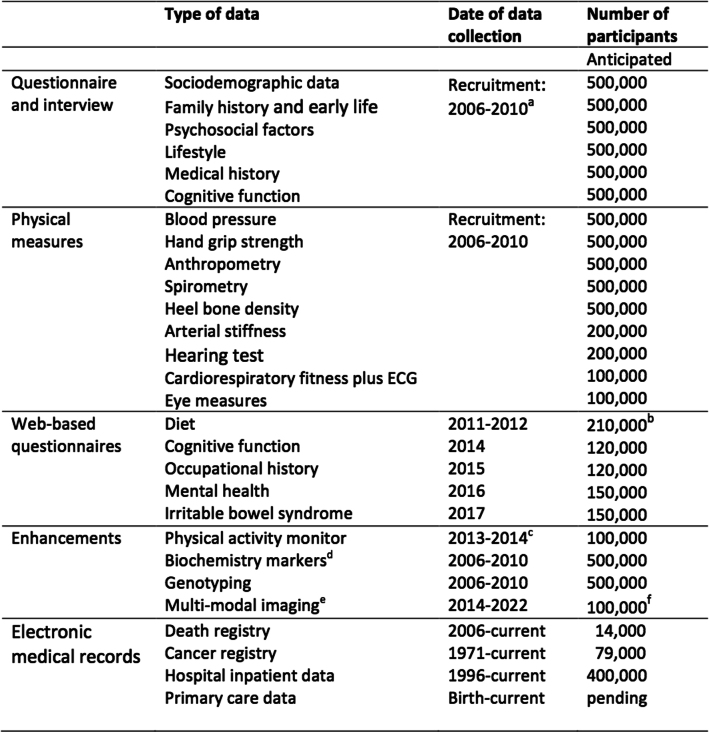
Types and dates of data collection in UK Biobank

^a^The baseline visit (including the touchscreen questionnaire, physical measures and biological sampling) was repeated approximately 5 years later (2012–2013) in a subset of 20,000 participants and in those who attended an imaging assessment centre (2014–2022).

^b^Includes 70,000 participants who completed the diet online questionnaire at the end of the recruitment visit^48^.

^c^A repeat assessment of physical activity on four occasions over a 12-month period is being collected on 2,5000 of these participants (2018–2019).

^d^Biochemistry markers were measured in the baseline sample for 500,000 participants and in the repeat assessment sample for 20,000 participants. The urinary biomarkers were made available in 2016; the serum and red blood cell markers available are pending (at the time of press).

^e^The imaging study includes brain, heart and body MRI, carotid ultrasound and 12-lead ECG scan and a full-body dual energy X-ray absorptiometry scan, plus a repeat of the baseline assessment (including biological sampling). Repeat imaging in a subset of participants is expected to start in 2019.

^f^Data are currently available for 25,000 participants, with the remaining 75,000 participants to attend over the next few years. See Supplementary Table 1 for further information about these data types.

**Extended Data Table 2:**
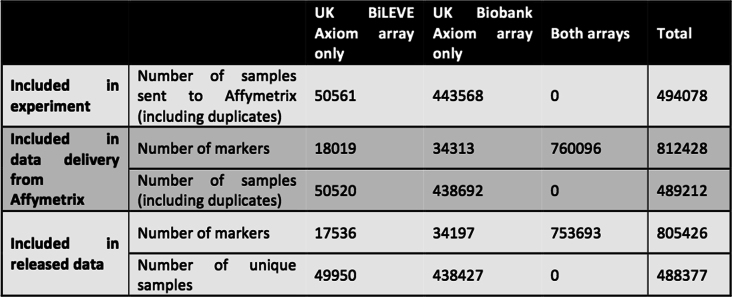
The number of markers and samples by genotyping array at main stages of the UK Biobank genotyping experiment

‘Data delivery from Affymetrix’ refers to the data produced by Affymetrix after applying their filtering (Supplementary Information). ‘Released data’ refers to the released genotype data, after applying quality control measures, as detailed in sections S2 and S3 of the Supplementary Information.

**Extended Data Table 3:**
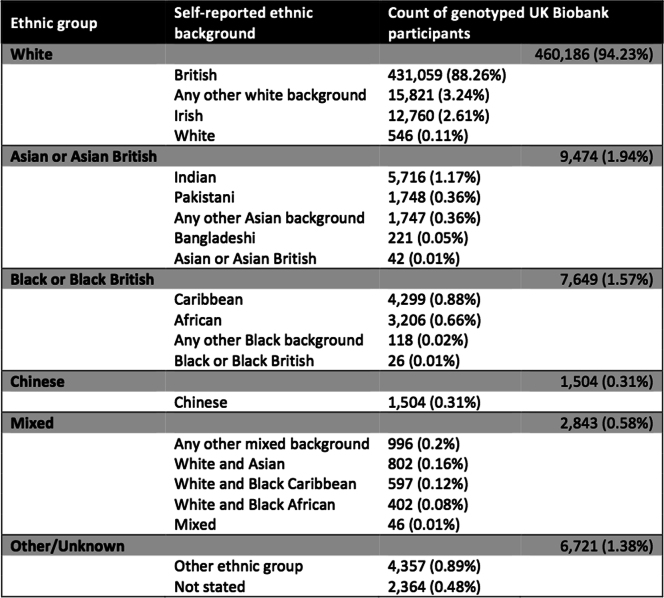
Counts and proportions of self-reported ethnic groups among 488,377 genotyped UK Biobank participants

Categories of self-reported ethnic background (UK Biobank data field 21000) and broader-level ethnic groups are shown here to reflect the two-layer branching structure of the ethnic background section in the UK Biobank touchscreen questionnaire^14^. Participants first picked one of the broader-level ethnic groups (for example, ‘white’), and were then prompted to select one of the categories within that group (for example, ‘Irish’). The broader-level groups are also shown here as an ethnic background category (‘white’ in column two) because a small proportion of participants only responded to the first question. In this table, we also combine the category ‘other ethnic group’ with an aggregated non-response category ‘not stated’, which includes all participants who did not know their ethnic group, or stated that they preferred not to answer, or did not answer the first question.

**Extended Data Table 4:**
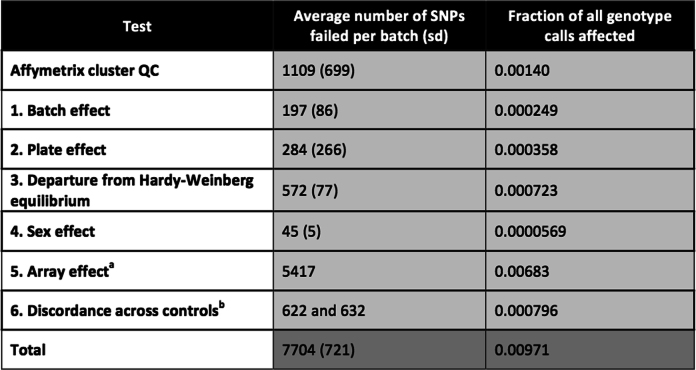
Failure rates for six marker-based quality tests

For all numbered tests, a marker (or marker within a batch) was set to missing if the test yielded *P* < 10^−12^, except in the case of test 6, for which a marker was set to missing if the test yielded <95% concordance. See Supplementary Information for details of each test (*n* = 463,844 samples). The total is not equal to the sum of all tests because it is possible for a marker to fail more than one test. Because the two arrays contain slightly different sets of markers, the total number of genotype calls used to compute the fractions is:

*N*_ukbb_ *L*_ukbb_ + *N*_ukbl_ *L*_ukbl_, in which *N* and *L* refer to the numbers of markers and samples typed on the UK Biobank Axiom array (ukbb) and samples typed on the UK BiLEVE Axiom array (ukbl) within the Affymetrix data delivery (see Supplementary Table 1).

^a^The array effect test was applied across all batches and only for markers present on both arrays, so we simply report the total number of markers that failed this test.

^b^The discordance test was applied across all batches, but not all markers are present on both arrays. The first value is the number of unique markers on the UK BiLEVE Axiom array that failed this test, and the second is for markers on the UK Biobank Axiom array.

**Extended Data Table 5:**
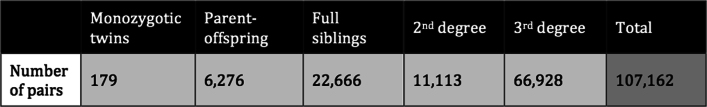
Summary of related pairs (third-degree relatives or closer) for the full UK Biobank cohort

Counts are derived from the kinship coefficients (see Methods). The count of monozygotic twins is after excluding samples identified as duplicates (Supplementary Information).

## Data Availability

The genetic and phenotype datasets generated by UK Biobank analysed during the current study are available via the UK Biobank data access process (see http://www.ukbiobank.ac.uk/register-apply/). Detailed information about the genetic data available from UK Biobank is available at http://www.ukbiobank.ac.uk/scientists-3/genetic-data/ and http://biobank.ctsu.ox.ac.uk/crystal/label.cgi?id=100314. The exact number of samples with genetic data currently available in UK Biobank may differ slightly from those described in this paper.
