# Supplementary material for: The UK Biobank resource with deep phenotyping and genomic data
Source: Nature. 2018 Oct 10;562(7726):203–9. doi: 10.1038/s41586-018-0579-z (PMC6786975; doi:10.1038/s41586-018-0579-z)
Supplement: Supplementary file 1 — This file contains Supplementary Material, including Supplementary Figures S1-S18 and Supplementary Tables S1-S13. [file 41586_2018_579_MOESM1_ESM.pdf]

nature SUPPLEMENTARY INFORMATION
https://doi.org/10.1038/s41586-018-0579-z

In the format provided by the authors and unedited.

# The UK Biobank resource with deep phenotyping and genomic data

Clare Bycroft[1,13], Colin Freeman[1,13], Desislava Petkova[1,12,13], Gavin Band[1], Lloyd T. Elliott[2], Kevin Sharp[2], Allan Motyer[3], Damjan Vukcevic[3,4], Olivier Delaneau[5,6,7], Jared O'Connell[8], Adrian Cortes[1,9], Samantha Welsh[10], Alan Young[11], Mark Effingham[10], Gil McVean[1,11], Stephen Leslie[3,4], Naomi Allen[11], Peter Donnelly[1,2,14] & Jonathan Marchini[1,2,14]*

[1]Wellcome Centre for Human Genetics, University of Oxford, Oxford, UK. [2]Department of Statistics, University of Oxford, Oxford, UK. [3]Melbourne Integrative Genomics and the Schools of Mathematics and Statistics, and BioSciences, The University of Melbourne, Parkville, Victoria, Australia. [4]Murdoch Children's Research Institute, Parkville, Victoria, Australia. [5]Department of Genetic Medicine and Development, University of Geneva, Geneva, Switzerland. [6]Swiss Institute of Bioinformatics, University of Geneva, Geneva, Switzerland. [7]Institute of Genetics and Genomics in Geneva, University of Geneva, Geneva, Switzerland. [8]Illumina Ltd, Chesterford Research Park, Little Chesterford, Essex, UK. [9]Nuffield Department of Clinical Neurosciences, Division of Clinical Neurology, John Radcliffe Hospital, University of Oxford, Oxford, UK. [10]UK Biobank, Adswood, Stockport, Cheshire, UK. [11]Big Data Institute, Li Ka Shing Centre for Health Information and Discovery, University of Oxford, Oxford, UK. [12]Present address: Procter & Gamble, Brussels, Belgium. [13]These authors contributed equally: Clare Bycroft, Colin Freeman, Desislava Petkova. [14]These authors jointly supervised this work: Peter Donnelly, Jonathan Marchini. *e-mail: marchini@stats.ox.ac.uk



© 2018 Springer Nature Limited. All rights reserved.

# The UK Biobank resource with deep phenotyping and genomic data

# Supplementary Material

**Clare Bycroft[1*], Colin Freeman[1*], Desislava Petkova[1,^*], Gavin Band[1], Lloyd T. Elliott[2], Kevin Sharp[2], Allan Motyer[3], Damjan Vukcevic[3,4], Olivier Delaneau[5,6,7], Jared O'Connell[8], Adrian Cortes[1,9], Samantha Welsh[10], Alan Young[11], Mark Effingham[10], Gil McVean[1,11], Stephen Leslie[3,4], Naomi Allen[11], Peter Donnelly[1,2†], Jonathan Marchini[2,1†‡]**

[1] Wellcome Trust Center for Human Genetics, University of Oxford, UK
[2] Department of Statistics, University of Oxford, UK
[3] Melbourne Integrative Genomics and the Schools of Mathematics and Statistics, and BioSciences, The University of Melbourne, Parkville, Victoria, Australia.
[4] Murdoch Children's Research Institute, Parkville, Victoria, Australia.
[5] Department of Genetic Medicine and Development, University of Geneva, 1 Michel Servet, Geneva, CH1211, Switzerland.
[6] Swiss Institute of Bioinformatics, University of Geneva, 1 Michel Servet, Geneva, CH1211, Switzerland.
[7] Institute of Genetics and Genomics in Geneva, University of Geneva, 1 Michel Servet, Geneva, CH1211, Switzerland.
[8] Illumina Ltd, Chesterford Research Park, Little Chesterford, Essex, CB10 1XL, United Kingdom.
[9] Nuffield Department of Clinical Neurosciences, Division of Clinical Neurology, John Radcliffe Hospital, University of Oxford, Oxford OX3 9DU, United Kingdom.
[10] UK Biobank, Units 1-4 Spectrum Way, Adswood, Stockport, Cheshire, SK3 0SA, UK
[11] Big Data Institute, Li Ka Shing Centre for Health Information and Discovery, University of Oxford, Oxford OX3 7LF, United Kingdom.
^ Current address: Procter & Gamble, Brussels, Belgium

* These authors contributed equally to this work.
† These authors jointly directed this work.
‡ To whom correspondence should be addressed: marchini@stats.ox.ac.uk

S 1 Introduction .... 3
S 1.1 The UK Biobank cohort .... 3
S 1.2 The UK Biobank Axiom genotyping array .... 3
S 1.3 Data releases .... 4
S 1.4 Details of DNA extraction and genotyping .... 5
S 2 Details of marker-based QC and analysis .... 8
S 2.1 Overview .... 8
S 2.2 Details of accounting for population structure in marker-based QC .... 8
S 2.3 Details of marker-based QC tests .... 9
S 2.4 Comparison of allele frequencies in UK Biobank and ExAC .... 14
S 3 Details of sample-based QC and analysis .... 16
S 3.1 Overview .... 16
S 3.2 Selection of markers for sample-based QC and analysis .... 18
S 3.3 Population structure (PCA) .... 18
S 3.4 Details of selecting a white British ancestry subset .... 22
S 3.5 Detecting outliers in heterozygosity and missing rates .... 24
S 3.6 Sex chromosome-specific sample QC .... 27
S 3.7 Inference of familial relatedness .... 28
S 4 Assessment of the UK Biobank Array for imputation .... 39
S 5 Imputation of classical HLA alleles .... 41
S 6 Details of genome-wide association tests for QC .... 44
S 6.1 Defining regions of association for the comparison with GIANT .... 44
S 7 Multiple trait GWAS and PheWAS .... 47
S 8 References .... 49
S 9 Appendix .... 52

# S 1 Introduction

## S 1.1 The UK Biobank cohort

UK Biobank is a prospective cohort study of over 500,000 individuals from across the United Kingdom. Participants, aged between 40 and 69, were invited to one of 22 centres across the UK between 2006 and 2010. Blood, urine and saliva samples were collected, physical measurements were taken, and each individual answered an extensive questionnaire focused on questions of health and lifestyle. This baseline information has been extended in a number of ways, including by genotyping the full cohort using a purpose-designed genotyping array. Table S1 provides URLs to further information about the data types described in Extended Data Table 1.

| | |
|---|---|
| Touchscreen questionnaire | http://biobank.ctsu.ox.ac.uk/crystal/label.cgi?id=100025 |
| Verbal interview | http://biobank.ctsu.ox.ac.uk/crystal/label.cgi?id=100071 |
| Physical measures | http://biobank.ctsu.ox.ac.uk/crystal/label.cgi?id=100006. |
| Web-based questionnaires | http://biobank.ctsu.ox.ac.uk/crystal/label.cgi?id=100089 |
| Physical activity monitor | http://biobank.ctsu.ox.ac.uk/crystal/label.cgi?id=1008 |
| Biochemistry markers | http://www.ukbiobank.ac.uk/wp-content/uploads/2013/11/BCM023_ukb_biomarker_panel_website_v1.0-Aug-2015.pdf |
| Urinary biomarkers | http://biobank.ctsu.ox.ac.uk/crystal/label.cgi?id=100083 |
| Imaging study | http://biobank.ctsu.ox.ac.uk/crystal/label.cgi?id=100003 |

**Table S1** : URLs that link to further information about the data types described in Extended Data Table 1.

## S 1.2 The UK Biobank Axiom genotyping array

The UK Biobank Axiom array from Affymetrix* was specifically designed by an expert group for the purpose of genotyping the UK Biobank participants. There are ~825,000 markers on the array, including both single nucleotide polymorphisms (SNPs) and small insertions and deletions (Indels).

The UK Biobank Axiom array was used to genotype ~450,000 of the ~500,000 UK Biobank participants. The other ~50,000 samples were genotyped on the closely related UK BiLEVE Axiom array. The UK BiLEVE project, for which the UK BiLEVE array was designed, aims to study the genetics of lung health and disease, and so those ~50,000 individuals were selected based on lung function and smoking behaviour from participants with self-declared European ancestry[1]. Otherwise, the UK BiLEVE cohort and the rest of UK Biobank differ only in small details of the DNA processing stage (e.g. UK BiLEVE samples were manually transferred from storage to plates for DNA extraction[2]).

The two genotyping arrays are very similar, with over 95% common marker content. The UK Biobank Axiom array is an updated version of the UK BiLEVE Axiom array, and

---

* Now part of Thermo Fisher Scientific.

† This artefact involved a subset of SNPs and a very small number of samples (~300), so it only affects a very small proportion of all the data (details in Section S 2.3). We excluded these SNPs in our sample-based QC and analysis as a precaution only.

it includes additional novel markers (such as cancer-related markers), which replaced a small fraction of the markers used for genome-wide coverage. The array annotation files for both the UK BiLEVE and the UK Biobank Axiom arrays are available as part of the UK Biobank resource, and further details of the array design are available in the UK Biobank Axiom Array content summary[3]. The positions of markers in this release are reported in coordinates of the genome build, Genome Reference Consortium Human Reference 37 (GRCh37).

## S 1.3 Data releases

The full data release contains the cohort of successfully genotyped samples (488,377). An interim release of genotype data in May 2015 comprised genotype calls for 152,736 samples. Subsequent to the interim release, changes were made by Affymetrix to their genotype calling pipeline[4] and there were some changes to the quality control pipeline. These changes were applied to the full cohort, which means that some small number of genotype calls in the final release may have changed since the interim data release (Figure S1).

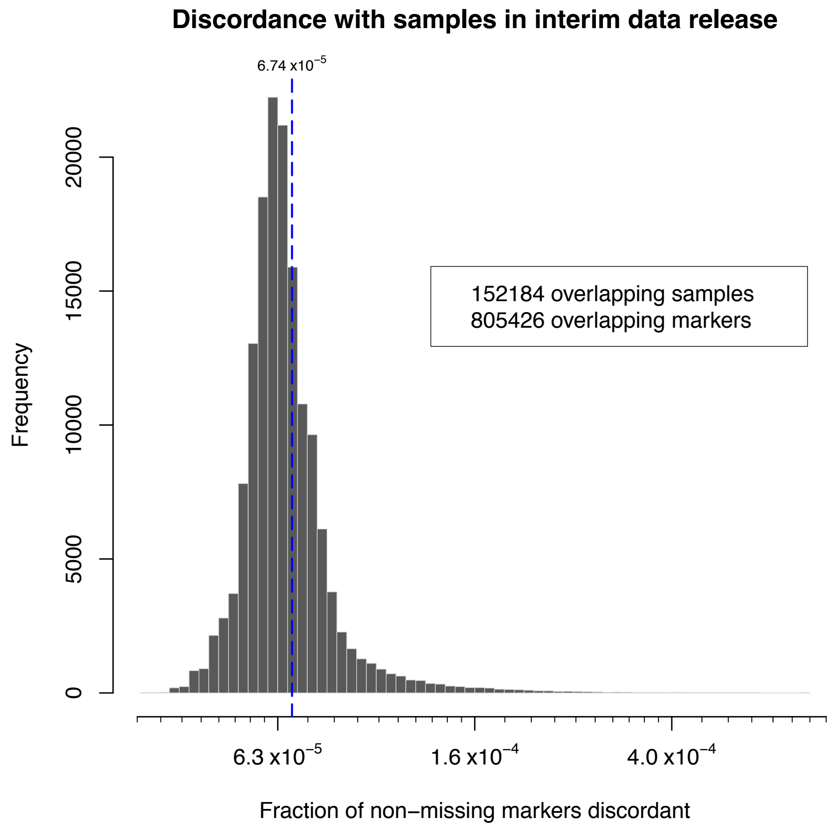


**Figure S1 | Comparison of genotype calls with genotype calls in interim release data.** We measured the discordance between the genotype calls in the interim release dataset and those in the current release. That is, for each sample that was included in both datasets, we measured the fraction of genotype calls that are non-identical, out of all the genotype calls that were not missing in both datasets for that sample. The dashed vertical line shows the mean discordance rate. The histogram is on the $\log_{10}$ scale but annotated with the original values.

# S 1.4 Details of DNA extraction and genotyping

### S 1.4.1 Sample storage and DNA extraction

The blood samples collected from participants are held at the UK Biobank facility in Stockport, UK[5]. Samples for genotyping were picked by robot to a 96-position destination rack (a plate) ready for DNA extraction (94 samples per plate leaving two spaces for the addition of two controls, as noted below). Importantly, this automated sample retrieval process was designed such that experimental units such as plates or timing of extraction do not correlate systematically with baseline phenotypes such as age, sex and ethnic background, or the time and location of sample collection. This was achieved via a sample selection algorithm that ensures each destination rack contains samples with a mixture of baseline characteristics and collection centres. During DNA extraction, the DNA concentration and purity were assessed[2]. Samples failing to meet defined thresholds were not submitted for genotyping; where possible these samples were re-processed at a later date. Full details of the UK Biobank sample retrieval and DNA extraction procedures can be found in[2,6].

A set of blind spike duplicates were also deliberately included in the genotyping experiment as a validation tool[2]. These are samples where an extra aliquot from the same participant was submitted for genotyping. The choice of blind spike duplicates was determined as samples were being extracted. One blind spike duplicate was included in the first 500 plates processed for the samples genotyped on the UK BiLEVE array and one blind spike duplicate was included on one plate in each shipment (approximately 70 plates) for the samples genotyped on the UK Biobank array. All plate positions (except spaces for controls) were used as a position for blind spike duplicates. The position of a blind spike duplicate on any given plate was chosen randomly by a lab operator from the set of positions that were previously unused for blind spike duplicates (repeated as necessary). The sample chosen to be duplicated was the sample in the same position on the plate that was processed either before or after the plate with the blind spike duplicate. We present analysis of the genotype calls for these samples in Section S 3.7.3.

### S 1.4.2 Genotype assay and calling

All samples were genotyped at the Affymetrix Research Services Laboratory in Santa Clara, California, USA. Upon receipt of a 96-well plate containing 94 UK Biobank samples, Affymetrix added two control individuals (from 1000 Genomes) to the same well positions on each plate: HG00097 to well A12 and HG00264 to well E12. Samples were processed in plates on the Affymetrix GeneTitan® Multi-Channel (MC) Instrument. Genotypes were then inferred from the resulting intensities in 106 batches of around 4,700 samples each (~4,800 including the controls). Eleven batches contain individuals typed on the UK BiLEVE Axiom array, and the other 95 batches contain individuals typed on the UK Biobank Axiom array (see Appendix Table S12). After all samples were genotyped, a set of poorly performing samples

was re-genotyped (i.e. a new set of intensities measured), and those that subsequently performed better were combined into 'Batch_b095'[7].

Affymetrix assays genetic markers using "probe sets": a set of probes targeting a particular marker. The fluorescence intensity of two alleles is measured and used to infer an individual's genotype at the marker. Individuals with the same genotype at any given marker will cluster together in a two-dimensional intensity space (one dimension for each targeted allele). Briefly, genotype calling involves inferring properties of these clusters within each batch and assigning each sample a genotype (or leaving the call missing) based on its position in intensity space. Figure S2 shows the intensities and genotype calls for an example marker. Technical details of Affymetrix's laboratory process are available in [7], and details of the genotyping calling routine specific to the UK Biobank project are available in [4].

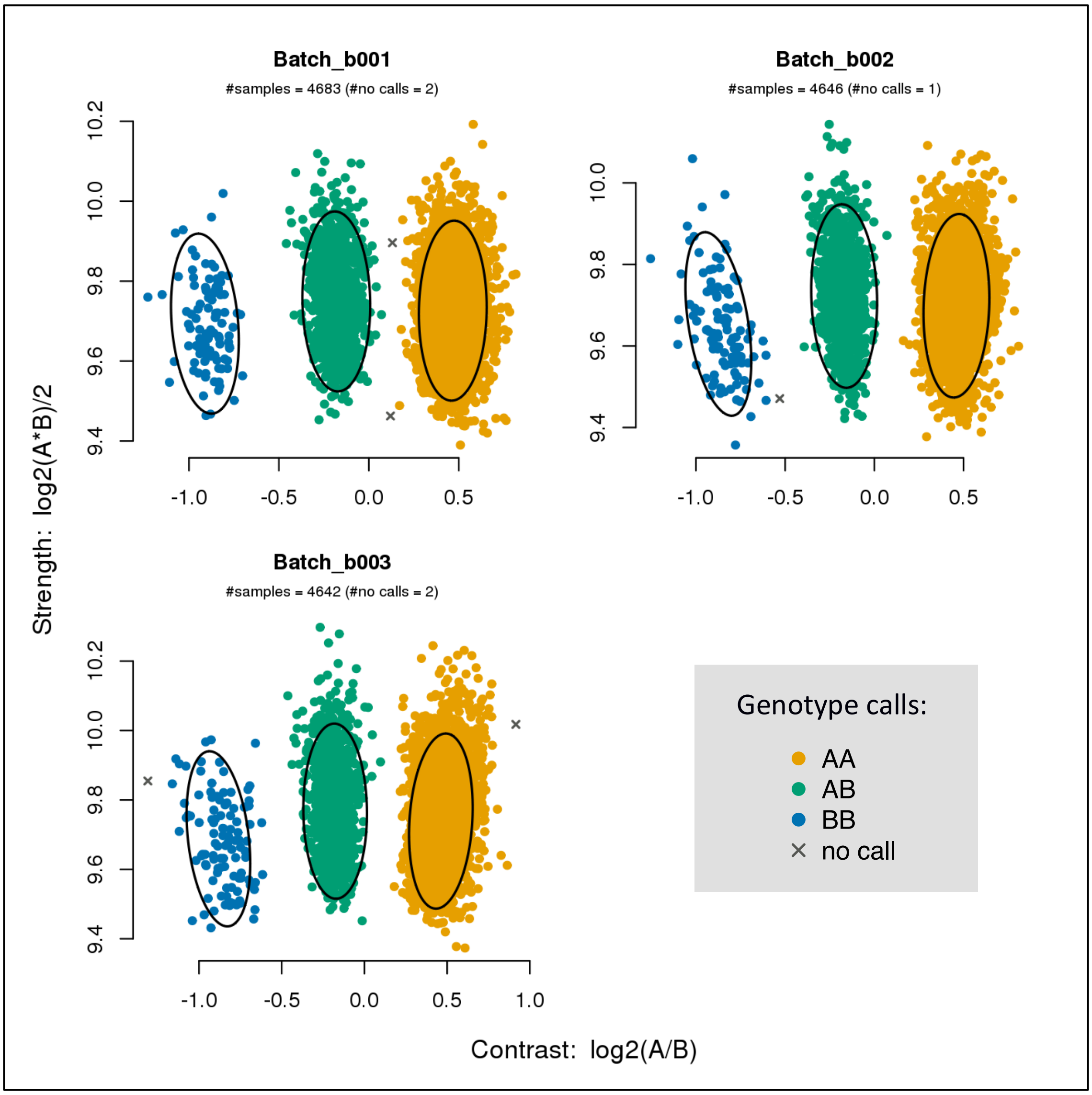


**Figure S2 | Example of intensity data and genotype calls for a marker in three batches.** Each point represents one sample and is coloured according to its inferred genotype at this marker. The x and y axes are transformations of the intensities for probes targeting allele "A" and allele "B". The ellipses indicate the location and shape of the posterior probability distribution (2-dimensional multivariate Normal) for each genotype cluster, such that 85% of the probability density falls inside the ellipse.

### S 1.4.3 Filtering by Affymetrix

The purpose-designed UK Biobank Axiom array attempts to assay a large number of markers (SNPs or Indels) that have not been previously genotyped using Affymetrix technology. In order to maximize the chances of such markers being successfully assayed, some were typed using more than one probe set. For each of these markers Affymetrix recommended a single probe set that had performed best across all batches and these recommendations were adopted throughout. Affymetrix also applied filters to each batch separately to exclude markers with poor cluster properties. If a marker did not meet the Affymetrix success criteria in a given batch, it was set to missing for all samples in that batch.

As expected with a novel array, a small number of markers exhibited sub-optimal and/or complex clustering patterns across all or many batches and were also excluded from the final data release. Some markers assayed on the array were known, or suspected to have more than two segregating alleles. Such multi-allelic markers require special treatment in array design and genotype calling, and these have also been excluded from the current data release.

For any of the above reasons a total of 35,014 unique markers were excluded from the data. This is made up of 31,518 markers on the UK Biobank Axiom array and 29,296 markers on the UK BiLEVE Axiom array, which is less than 5% of all markers present on either array.

Affymetrix also checked sample quality (such as DNA concentration and genotype call missing rates) and genotype calls were provided only for samples with satisfactory metrics. More information about the Affymetrix calling algorithms and filtering protocols is available in [4,7].

# S 2 Details of marker-based QC and analysis

## S 2.1 Overview

Genotype calling by Affymetrix resulted in a dataset of 489,212 individuals typed at 812,428 markers with which to carry out further QC. Our QC pipeline aimed to address issues specific to a large-scale, ancestrally diverse dataset which was genotyped in many batches (106, with ~4700 individuals each), using two arrays, and which will be used by many researchers with a wide variety of research questions. These factors mean that some quality control metrics commonly used, for example, in genome-wide association studies (GWAS), are not directly applicable in this context. We used a variety of approaches in our QC procedures to account for effects such as the large cohort size, population structure, and batch-based genotype calling. The amount of data affected by our QC is summarized in Extended Data Table 4 and Figure 2.

After applying QC we assessed the quality of the released dataset by comparing genotype calls across replicates included in the experiment; as well as comparing allele frequencies in the UK Biobank with those in an external source, the Exome Aggregation Consortium (ExAC) (see Section S 2.4).

## S 2.2 Details of accounting for population structure in marker-based QC

Many QC tests are ineffective in the context of population structure. We therefore applied all marker-based QC tests using a subset of 463,844 individuals drawn from the largest ancestral group in the cohort (European). Here we describe the procedure to identify such samples using principal component analysis (PCA) and two-dimensional clustering.

We first downloaded 1000 Genomes Project Phase 1 data in Variant Call File (VCF) format[8] and extracted 714,168 SNPs (no Indels) that are also on the UK Biobank Axiom array. We selected 355 unrelated samples from the populations CEU, CHB, JPT, YRI, and then chose SNPs for principal component analysis using the following criteria:

- MAF ≥ 5% and HWE p-value > $10^{-6}$, in each of the populations CEU, CHB, JPT and YRI.
- Pairwise $r^2 \leq 0.1$ to exclude SNPs in high LD. (The $r^2$ coefficient was computed using *plink* [9] and its 'indep-pairwise' function with a moving window of size of 1000 kilo-bases (Kb) and a step-size of 80 markers).
- Removed C/G and A/T SNPs to avoid unresolvable strand mismatches.
- Excluded SNPs in several regions with high PCA loadings (after an initial PCA).

With the remaining 40,220 SNPs we computed PCA loadings from the 355 1,000 Genomes samples, then projected all the UK Biobank samples onto the 1st and 2nd principal components. All computations were performed with Shellfish (http://www.stats.ox.ac.uk/~davison/software/shellfish/shellfish.php).

Finally, for each batch separately, we applied an outlier detection algorithm *aberrant* [10] (with the lambda parameter set to 20) to isolate the largest cluster of samples from the rest in the batch, based on the two leading PCs. In UK Biobank, the largest cluster is composed of individuals with European ancestry.

## S 2.3 Details of marker-based QC tests

We performed six tests designed to check, among other things, for consistency across different experimental factors. Specifically, we tested for batch effects, plate effects, departures from Hardy-Weinberg equilibrium (HWE), sex effects, array effects, and discordance across control replicates. All tests (except for discordance across controls) were applied using the genotype calls for a set of 463,844 ancestrally homogeneous individuals (see Section S 2.2). The details of each test are described in Section S 2.3.3, and Figure S3 shows examples of affected markers.

Four of the tests (batch effect, plate effect, departures from HWE, sex effect) were applied to each marker in each batch separately. For markers that failed at least one test in a given batch, we set the genotype calls in that batch to missing. Markers that failed any one of these tests in every batch were excluded from the dataset altogether. The other two tests (array effect and discordance across controls) were applied to each marker across all batches. Any marker that failed at least one of these two tests was also excluded from the dataset altogether. We applied each of these tests independently, so some markers, or marker/batch combinations may have failed more than one test. In all except one case (array effect) tests were applied to the union of markers on the two arrays.

In addition to the six tests, some of the genotype calls for a small number of samples (387) were likely compromised due to a rare artefact involving the digital misalignment of features on the array during image-capture. The intensities for these samples at a subset of SNPs were systematically shifted in a way that imitated a real genotype cluster, so were not highlighted by other QC tests but were identified in a principal components analysis. We set the genotype calls to missing only for these samples at a set of 34,921 markers that Affymetrix identified as likely affected (0.0037% of all genotype calls).

### S 2.3.1 Choice of p-value for hypothesis-based tests

The batch effect, plate effect, HWE, sex effect and array effect tests are hypothesis tests with an associated p-value. Any marker, or marker/batch combination with a p-value smaller than a fixed threshold we considered as failing the test. We used a p-value threshold of $10^{-12}$. This threshold was chosen so that we only set a marker/batch to missing if there is very strong evidence for deviation from the null hypothesis of any of these tests.

There are 5 kinds of hypotheses, 106 batches, ~50 plates per batch and ~800,000 markers, making a total of around $4.6 \times 10^9$ tests (accounting for plate-level and batch-level tests). A p-value of $10^{-12}$ can therefore be thought of as equivalent to a

family-wise error rate of at most 0.005. Many tests will be positively correlated, especially across batches for the same marker, so this is likely to be an upper bound on the probability that we observe an extreme test statistic just by chance.

### S 2.3.2 Treatment of haploid markers

For both the Y chromosome and Mitochondrial markers Affymetrix assessed the performance of the assay by visually inspecting cluster plots[4]. In addition, we applied all of the tests to haploid as well as diploid markers, except HWE, which only makes sense in the diploid case. For haploid markers (e.g. Mitochondria) we counted only two categories of genotypes instead of three. For the Y chromosome we ran each test only using males (as inferred by Affymetrix). For the sex-specific region of the X chromosome we ran each test separately using males only (haploid), females only (diploid), and both combined, but then used the smallest of the three p-values. The pseudo-autosomal regions (PAR) of the X chromosome were treated exactly as autosomal markers.

### S 2.3.3 Details of each test

***Batch effect***

In samples drawn from the same population we would not expect differences in genotype frequencies between batches at the same marker. Such differences might indicate that the marker was not genotyped as accurately in the batch that exhibits unusual genotype frequencies. We refer to these cases as batch effects. Batch effects can occur, for instance, when the sample intensities for a marker in one batch shift relative to the intensities in other batches. In rare cases, such a shift can cause the Affymetrix calling algorithm to miscall a genotype cluster that is not detected by the routine Affymetrix QC[4]. To detect such effects we tested whether we can reject the null hypothesis that a given batch has the same genotype frequencies as for all other batches combined. We used a Fisher's exact test on the 2×3 table of genotype counts (or 2x2 table for haploid markers). For markers that are only on one of the two genotyping arrays, only batches with samples typed on that array were included in the test. For all other markers, all 106 batches across both arrays were included.

***Plate effect***

Similar to batch effects, we would not expect differences in genotype frequencies between plates at the same marker. We refer to these cases as plate effects. Plate effects can occur when the intensities in one plate shift relative to the intensities of other plates within the same batch. To look for effects in a particular plate we tested whether we can reject the null hypothesis that the given plate has the same genotype frequencies as all other plates within the same batch combined, and then used the smallest p-value for that batch. As with the batch effect test, we used Fisher's exact test on the 2×3 table of genotype counts (or 2x2 table for haploid markers). We performed the test only using plates that are at least half-full within a batch, i.e. with 48 samples or more.

### *Departures from Hardy-Weinberg equilibrium*

We performed an exact test for departures from HWE within each batch using the exact test described in [11] and implemented by the authors of *plink*[12]. Since this is a test for departures from the expected counts of genotypes, given a set of allele frequencies, we only tested diploid regions of the genome, and females only on the sex-specific region of the X chromosome.

### *Sex effect*

We don't expect differences in genotype frequencies between males and females for all markers other than the Y chromosome. For autosomal markers, differences may be due to sequence homology on the X or Y chromosome, leading to different base-line intensities for males and females, and potentially incorrect genotype calls. For markers on the sex-specific region of the X chromosome, the genotype calling algorithm was applied separately for males and females because of the difference in chromosome copy number[13]. This introduces the possibility of different allele frequencies in males and females due to the automated calling algorithm performing differently for each of the sexes, rather than genuine frequency differences. We tested for this within each batch for autosomal and PAR X markers using a Fisher's exact test on the 2x3 table of genotype counts for males and females; and the 2x2 table of allele counts (not genotypes) for males and females for markers on the sex-specific region of the X chromosome.

### *Array effect*

The two arrays used for the UK Biobank cohort – the UK Biobank Axiom array and the UK BiLEVE Axiom array – have a large number of markers in common, but some aspects of the design of the physical genotyping array differed. These differences can have subtle effects on the distribution of observed intensities for a marker, and may result in differences in the behaviour of the genotype calling algorithm across the two arrays, for the same marker. We refer to this as an array effect. To identify markers affected by this we tested whether we can reject the null hypothesis that the set of individuals typed on the UK Biobank Axiom array has the same genotype frequencies as those typed on the UK BiLEVE Axiom array. We used Fisher's exact test on the 2×3 table of genotype counts across the two arrays (or 2x2 for haploid markers).

The participants who were typed on the UK BiLEVE Axiom array were selected from the whole cohort based on phenotypes involved in lung function and smoking behavior, and those with self-declared European ancestry[1]. Consequently, it is possible for array effects to occur as a result of genuine genotypic differences between the two sets of participants; for example, at loci associated with smoking behavior or lung function. While most of the markers with evidence of an array effect are scattered across the genome, we found one set of markers with low p-values clustered within and around the gene CHRNA3, a locus known to be associated with smoking behavior[1]. Since this signal is likely to reflect genuine phenotype-genotype associations and not an experimental artifact, we did not exclude any marker in this region on the basis of the array effect test. Specifically,

chromosome 15, positions 78.6 – 79.0 Mega-bases (Mb).

***Discordance across control replicates***

The DNA of two individuals from the CEU group of the 1000 Genomes project (HG00097 and HG00264) were used as controls for the UK Biobank experiment. Specifically, each plate processed by Affymetrix contained two wells assigned to the controls, and resulted in a total of 5,817 and 5,424 successfully genotyped replicates for the two individuals, respectively. This provides a further opportunity to assess the quality of the genotype calling for a specific marker because we know that the true underlying genotype for the replicates should be the same. For each individual of the control individuals, and each marker, we computed a discordance metric, $d$,

$$d = 1 - \frac{\max(n_{AA}, n_{AB}, n_{BB})}{n_{AA} + n_{AB} + n_{BB}}$$

where $n_{AA}, n_{AB}, n_{BB}$ is the number of times the genotypes AA, AB, and BB are called for the individual at that marker. For haploid markers $n_{AB}$ is always 0 and $n_{AA}$ and $n_{\boldsymbol{BB}}$ correspond to the calls A and B. Any marker with $d \geq 0.05$ (or equivalently $<$ 0.95 concordance) for at least one of the two control individuals was excluded from the released dataset.

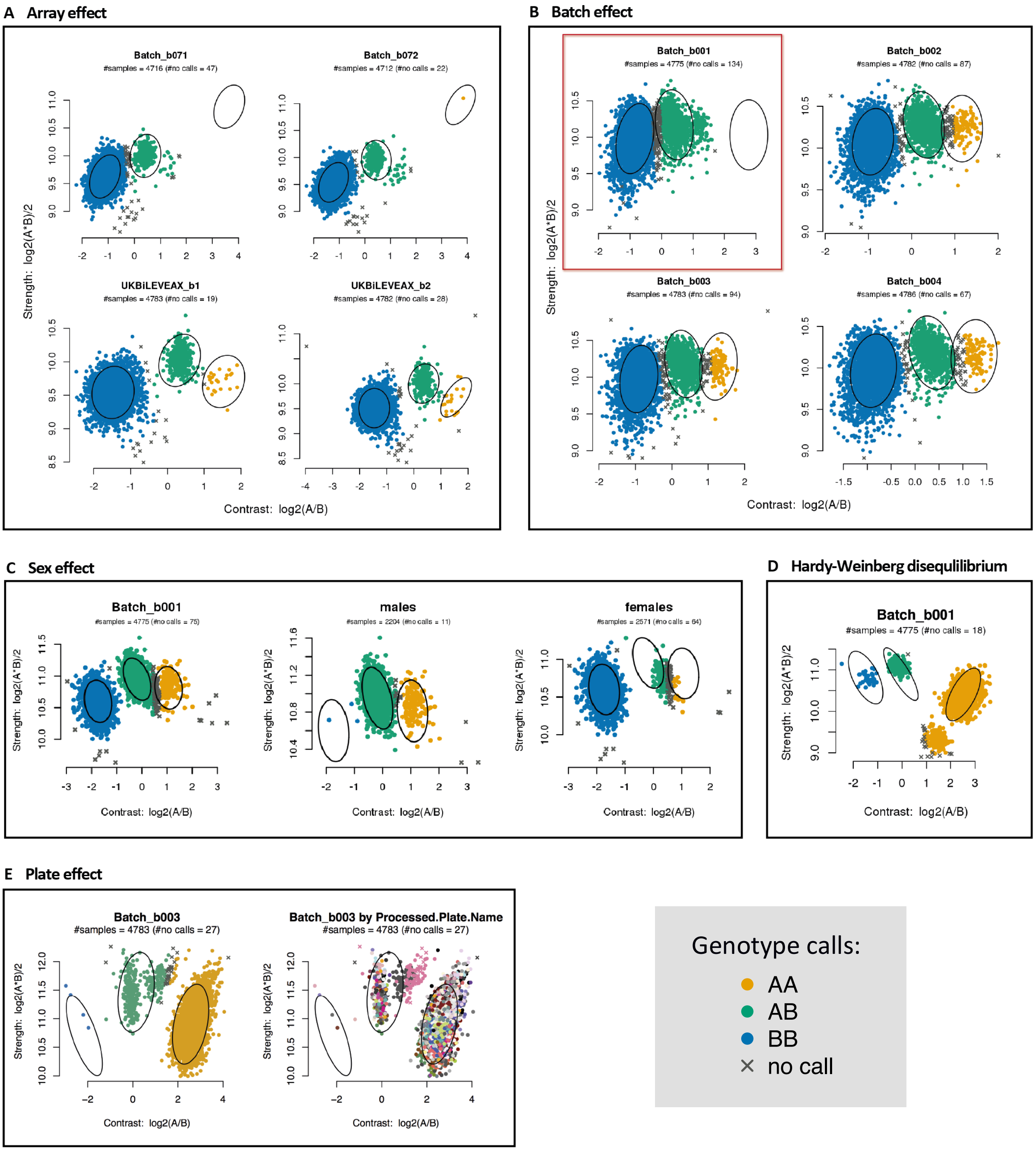


**Figure S3 | Examples of markers failing quality control tests.** Each sub-figure shows an example of a marker exhibiting the properties that our QC tests were designed to identify (p-value < $10^{-12}$). See Section S 2.3.3 for details of each test. Each plot shows the samples within the stated batch coloured according to their inferred genotype, as in Figure S2. The limits of the axes vary depending on the range of intensities observed in each batch. **A)** The top two plots show batches typed on the UK Biobank Axiom array, and the bottom two show batches typed on the UK BiLEVE Axiom array. The third cluster (orange; minor homozygotes) has been called as homozygote (green) in the UK Biobank Axiom array batches, likely due to the presence of the outlier in Batch_b072. **B)** Genotype calls in the highlighted batch (Batch_b001) contain no minor homozygotes (orange), unlike the other three batches shown. **C)** One batch is shown here, but also with males and females plotted separately. There are only two clusters for each of males and females, but they are shifted relative to each other so form what appears to be three clusters when combined. This is an autosomal marker, so males and females are genotyped together. **D)** The presence of a fourth cluster suggest that this marker involves variation more complex than a bi-allelic marker. The samples in the fourth cluster that were called as major homozygotes causes the genotype counts to violate HWE. **E)** This batch for this marker contains two plates (shown as dark brown and pink dots in the right-hand plot) that are systematically shifted in intensity space.

## S 2.4 Comparison of allele frequencies in UK Biobank and ExAC

We compared allele frequencies between UK Biobank and ExAC within sets of samples of European ancestry. Results are shown in Figure 2C and Figure S4.

We downloaded ExAC data (VCF files) from:
ftp://ftp.broadinstitute.org/pub/ExAC_release/release1/ExAC.r1.sites.vep.vcf*

For the ExAC data we used allele counts for the non-Finnish European population group (33,370 samples). For the UK Biobank we used the set of 463,844 European-ancestry samples (see Section S 2.2 for how these were identified), and who did not report Finland as their birthplace (there are ∼160 Finland-born participants in the UK Biobank cohort). We compared a set of 91,298 markers, which are in the released genotype dataset, are on both genotyping arrays, and have more than 90% call rate in both UK Biobank and ExAC. We merged data for the two studies by requiring markers to match on chromosome, position, and the reference and alternative alleles (all markers in the UK Biobank released data are bi-allelic). We report the frequency of the allele that is minor in the UK Biobank, so some markers could have allele frequency > 0.5 in ExAC.

We expect some discrepancies due to subtle differences in population structure within the two studies, as well as differences in the sensitivity and specificity of the two technologies (exome sequencing and genotyping arrays). There are also a small number of ∼300 markers that have very different allele frequencies. They comprise ∼0.3% of all markers in the comparison, or ∼0.5% of all markers with MAF > 0.001 in at least one study. Namely, 179 markers for which the frequency is > 0.001 in ExAC (usually corresponding to more than 60 copies of the minor allele) but zero in the UK Biobank; and 35 markers where the reverse is the case. A further 73 markers have frequency > 0.75 in ExAC, indicating a mis-annotation of the alternative allele in either the UK Biobank arrays or ExAC. From visual inspection of intensity plots (such as those shown in Figure S2) for a subset of these markers we concluded the following. In the cases where MAF is zero in UK Biobank there is no evidence of a heterozygous cluster, possibly because the probes for one or both of the alleles are not working. In the cases where the frequency is zero (or close to 1) in ExAC, most appear to be genotyped well in UK Biobank. However, many of these markers are multi-allelic (in ExAC) or indels, which would be consistent with either annotation error on the UK Biobank arrays or in ExAC, or mapping errors in the sequence data in regions of more complex variation.

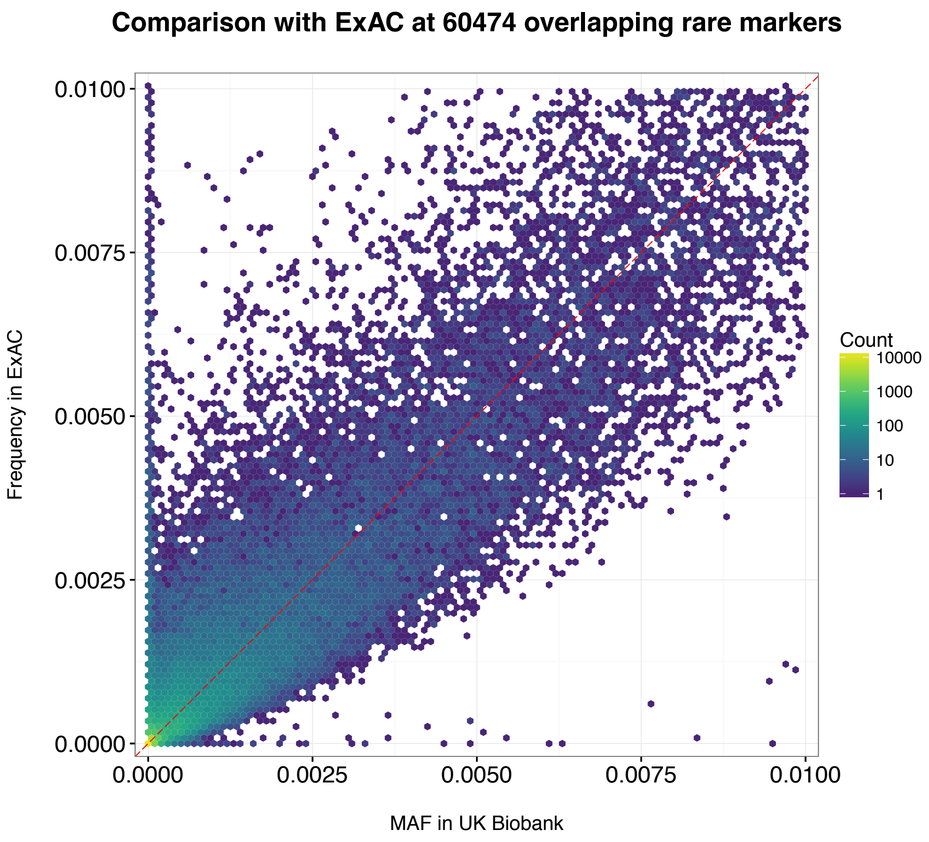


**Figure S4 | Allele frequency comparison with ExAC at rare markers.** Each hexagonal bin is coloured according to the number of markers falling in that bin, as indicated by the key (note the $\log_{10}$ scale). The dashed red line shows x=y. The comparison for variants at all frequencies is shown in Figure 2C.

# S 3 Details of sample-based QC and analysis

## S 3.1 Overview

Our pipeline for sample-based QC and analysis was designed to identify samples with poor quality genotype calls, find related individuals, and provide a quantitative description of ancestral diversity of the cohort based on information in the genetic data. We used a set of 621,642 high quality SNPs that were typed on both arrays to ensure that metrics computed across many markers reflect properties associated with each sample. Several of the analyses we conducted were dependent on each other. For example, adjusting the heterozygosity metric to account for population structure first requires computation of principal components. Figure S5 shows all the key interdependences within the pipeline, and the relevant sections in this document.

A small number of samples were excluded from the data release subsequent to running this pipeline. The counts of samples reported in this section are based on the dataset prior excluding those samples, so some values may differ slightly from that observed in the released data.

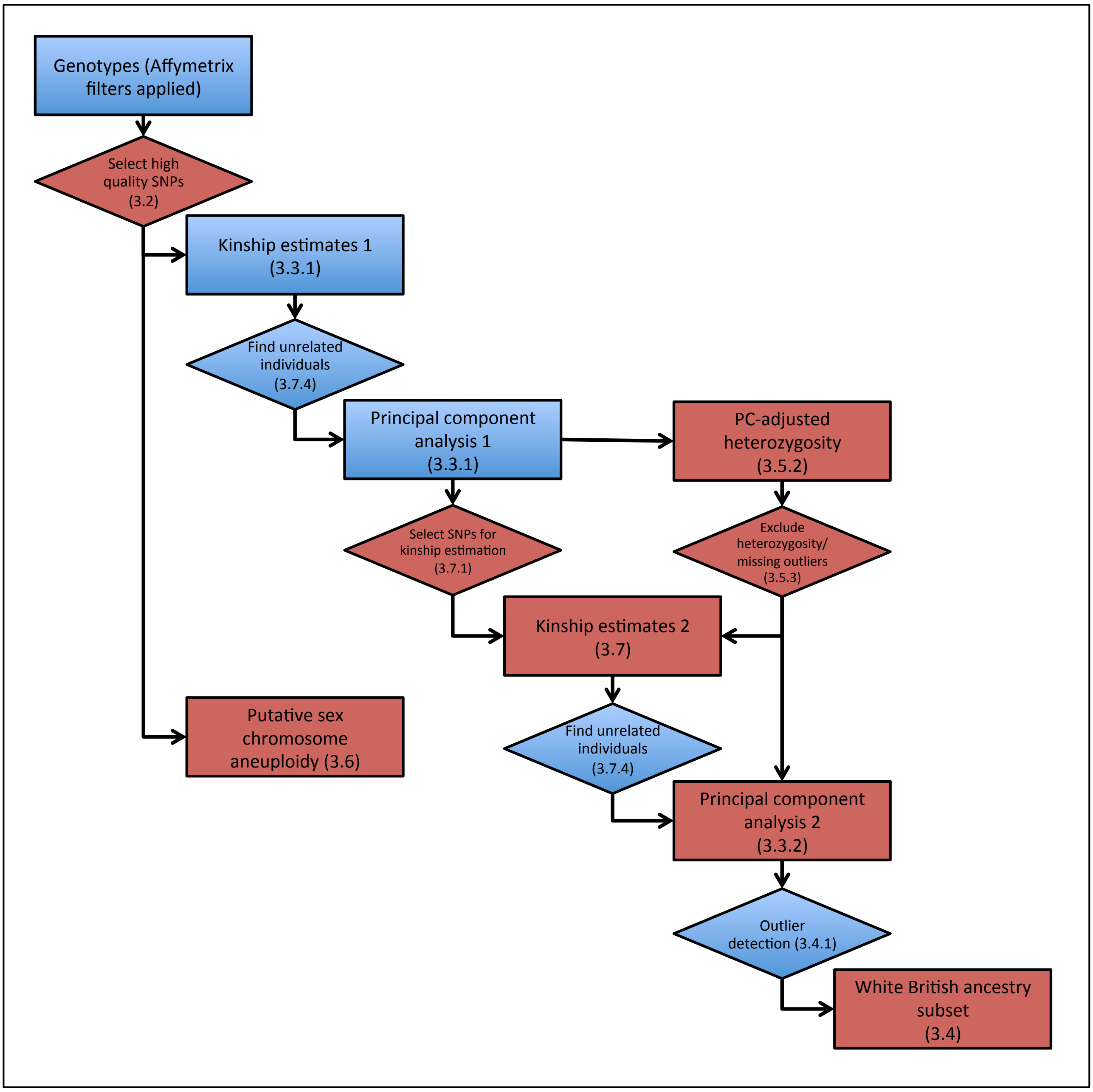


**Figure S5 | Overview of pipeline for sample-based analyses and quality control.** Rectangles represent data or computed variables; diamonds indicate key processes that link the different analyses. For example, the computation of “Principal component analysis 1” requires “Kinship estimates 1” in order to exclude related individuals from the PC computation. Numbers denote the relevant section in Supplementary. Data that is associated with the elements shown in red are made available to researchers.

## S 3.2 Selection of markers for sample-based QC and analysis

We selected 621,642 markers (605,876 autosomal; 15,766 on X and Y chromosomes), such that they fulfil the following criteria:

- In both of the two arrays.
- Is a SNP (not an Indel).
- Passed QC in all 106 batches (see Section S 2.3).
- MAF among all UK Biobank samples > 0.0001.
- Is not in the list of SNPs affected by the 'image' artefact[†].

All analyses described in Section S 3 used these SNPs only, or a subset of these where stated. The X and Y chromosome markers were only used for the sex chromosome-specific sample QC and analysis (Section S 3.6). Wherever possible, the list of SNPs used in a specific analysis (e.g. PCA) is provided to researchers.

## S 3.3 Population structure (PCA)

We used principal component analysis (PCA) to capture population structure within the UK Biobank cohort. The PCA we conducted serves two purposes: to account for population structure in other sample-based QC metrics (such as heterozygosity); and to assist the research community by computing a metric that is widely used as an indicator of genetic ancestry (complementary to self-reported ethnicity), and is widely used as a method for assessing and potentially controlling for population structure in GWAS [14,15].

Principal components should ideally be computed using a subset of high quality, unrelated samples. However, the metrics used to find related samples, as well as poorer quality samples themselves require information about population structure (see Sections S 3.5 and S 3.7). We therefore conducted an initial round of PCA, computing just the top 8 PCs, using a set of unrelated samples based on an initial round of kinship estimation. We used the results of this analysis to compute PC-adjusted heterozygosity as well as refine the relatedness inference. Having then identified a set of high quality, unrelated samples, we conducted a second round of PCA, computing the first 40 PCs. Results of the second round are made available to researchers and visualised in Figure 3A, Figure S6-S7, Extended Data Figure 3, and discussed in the main text.

### S 3.3.1 Details of PCA round 1

We first estimated kinship coefficients between all samples using the software *KING*[16], with the command "—related –degree 3", and used these results to find a set of unrelated individuals. Note this is separate from the final relatedness inference described in Section S 3.7. Next we excluded samples with the following properties:

† This artefact involved a subset of SNPs and a very small number of samples (~300), so it only affects a very small proportion of all the data (details in Section S 2.3). We excluded these SNPs in our sample-based QC and analysis as a precaution only.

- Missing rate on autosomes > 0.02.
- Not in a set of unrelated individuals (see Section S 3.7.4).
- Mismatch between inferred sex and self-reported sex.

We also excluded SNPs with the following properties:

- Missing rate > 0.015.
- MAF < 0.01.
- In regions of long-range linkage disequilibrium (LD) e.g. inversions. The boundaries we used are in Appendix Table S13).

We then pruned the SNPs to a set of independent markers such that pairwise $r^2$ < 0.1, using windows of 1000 markers and a step-size of 80 markers.

These filters were applied to the genotype data using the appropriate *plink* commands in the order described, and resulted in a set of 147,551 SNPs and 406,247 samples with which to compute PCs. We computed the top 8 PCs using *fastPCA*[17] with options 'numoutevec' = 8 (otherwise program defaults). We computed SNP-loads for each PC by carrying out the appropriate matrix multiplications based on mean-centred and variance-scaled genotypes, and the PC scores computed by *fastPCA*. We then projected all samples onto the PCs using the SNP-loads.

### S 3.3.2 Details of PCA for release

We filtered the genotype data using the same criteria as above, but with additional sample exclusion criteria based on a second round of familial relatedness inference using a specially filtered set of SNPs (see Section S 3.7), and having identified a small number of lower quality samples (see Section S 3.5). Specifically, we excluded samples with the following properties:

- Missing rate on autosomes > 0.02.
- Not in a set of unrelated individuals (see Sections S 3.7.1 and S 3.7.4).
- In the list of outliers based on heterozygosity and missing rates (see Section S 3.5).
- Mismatch between inferred sex and self-reported sex.

These filters were applied using *plink*, and resulted in a set of 147,606 SNPs and 407,599 samples with which to compute PCs. We computed the top 40 PCs using *fastPCA*[17] with options 'numoutevec' = 40; 'fastdim' = 50; 'fastiter' = 40. We computed SNP-loads for each PC by carrying out the appropriate matrix multiplications based on mean-centred and variance-scaled genotypes, and the PC scores computed by *fastPCA*. We then projected all samples onto the PCs using the SNP-loads.

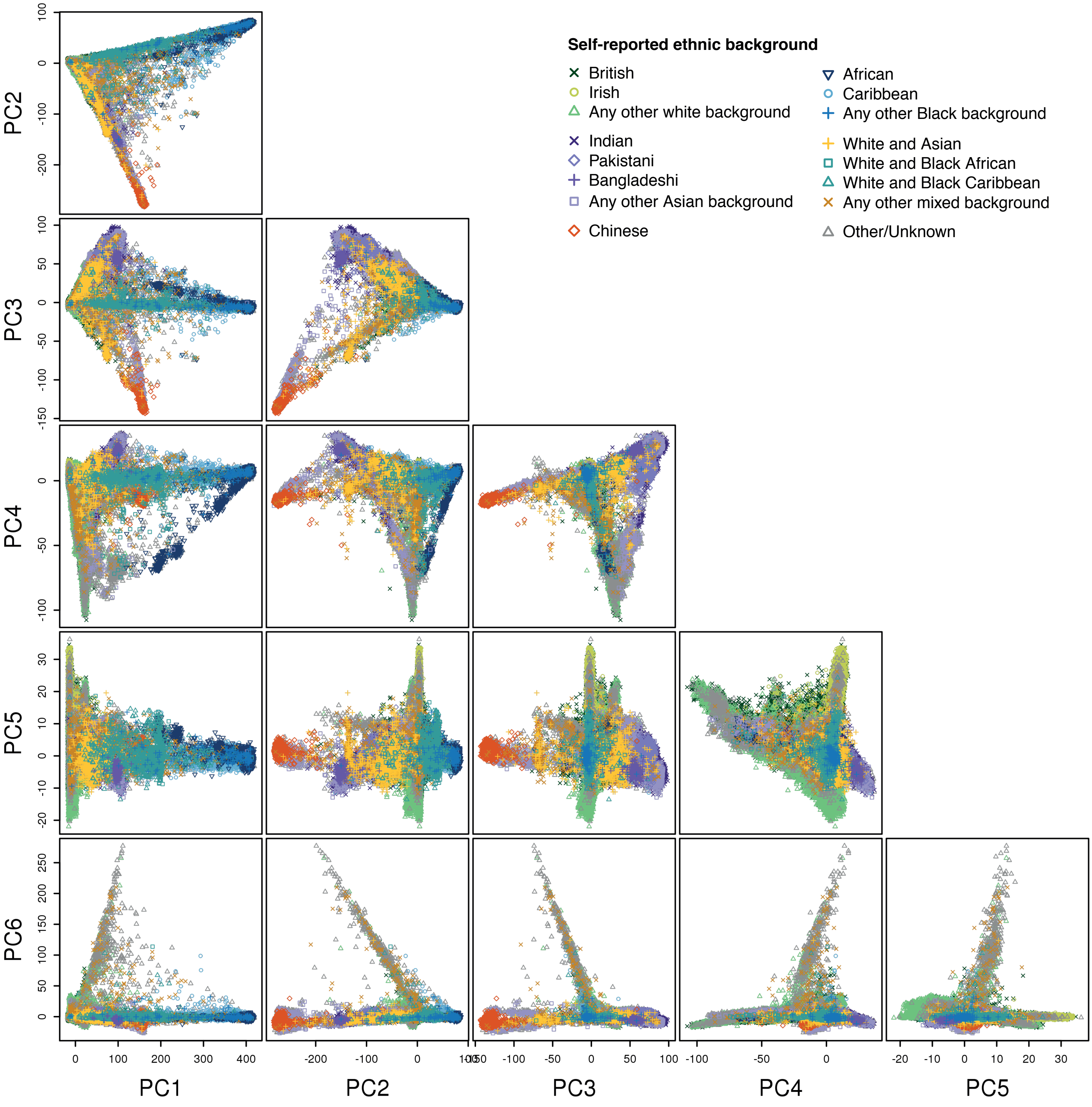


**Figure S6 | All pairs of the first 6 principal components in PCA on UK Biobank genotype data.** Each plot shows PC scores for UK Biobank samples for pairs of successive principal components. Each point represents a UK Biobank participant (n=488,377 samples) and is coloured according to their self-report ethnic background as defined in the key.

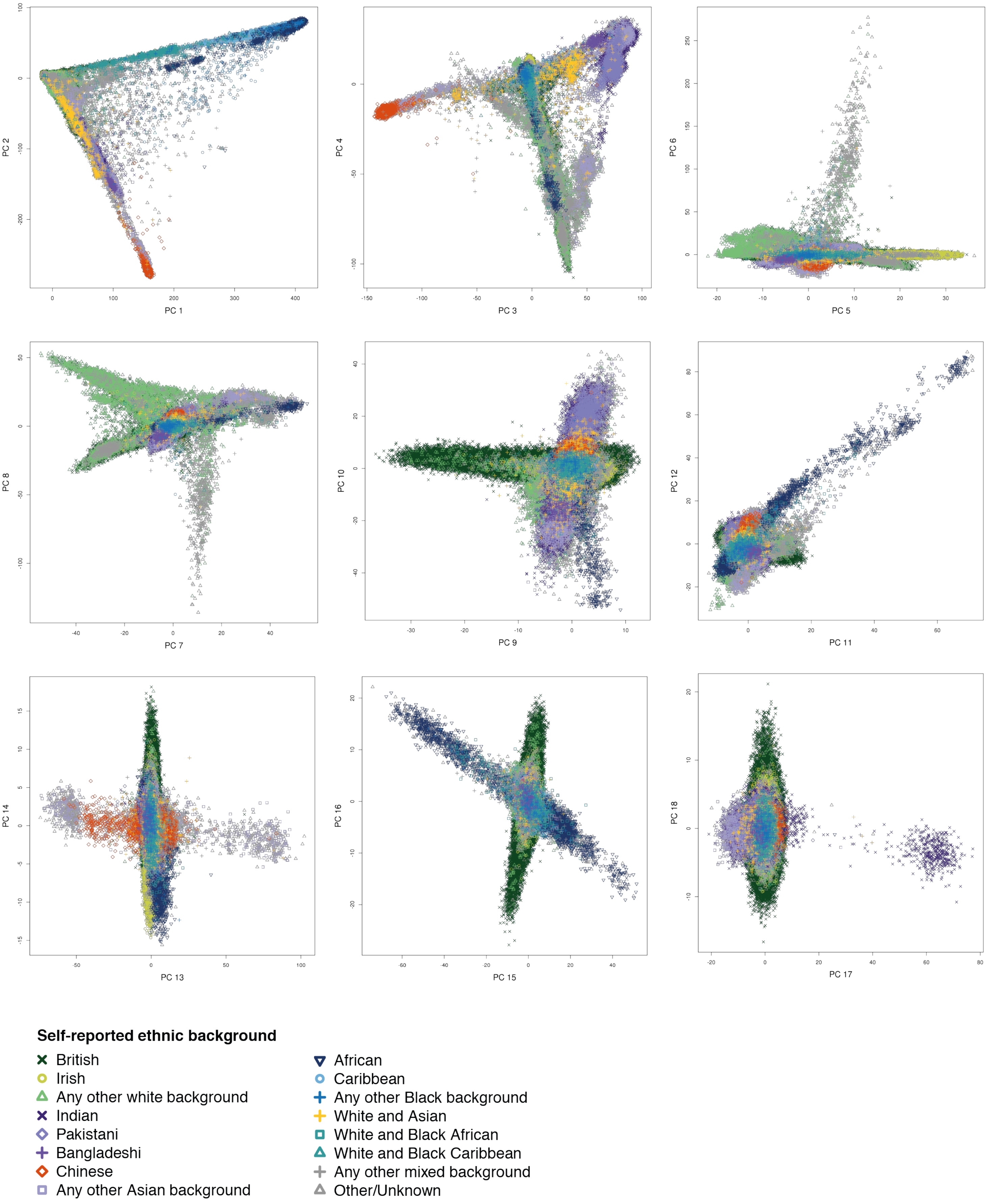


**Figure S7 | The first 18 principal components in PCA on UK Biobank genotype data.** Each plot shows PC scores for UK Biobank samples for pairs of successive principal components. Each point represents a UK Biobank participant (n=488,377 samples) and is coloured according to their self-report ethnic background as defined in the key. This figure shows results of the PCA for release (see Section S 3.3.2). Results for all 40 PCs are visualised in Extended Data Figure 3.

## S 3.4 Details of selecting a white British ancestry subset

Researchers wanting to reduce the effects of strong population structure on their analysis may want to use a set of individuals with relatively homogenous ancestry. A majority of participants in the UK Biobank cohort report their ethnic background as "British", within the broader-level group "White" (88.26%) (Extended Data Table 3). We use this information, as well as the genetic data, to provide a list of 409,728 individuals (84%) who self-report as "British" and who have very similar ancestral backgrounds according to the PCA. We refer to this set of individuals as the "white British ancestry subset".

We first selected 431,059 (88.26%) individuals who report their ethnic background as "British", within the broader-level group "White" (Extended Data Table 3). We used a Bayesian outlier detection algorithm implemented in the R package *aberrant*[10], to isolate the largest cluster of samples from the rest, using PCs 1 - 6. *aberrant* takes a parameter (Lambda), which effectively sets how tight the 'inlier' cluster is. We set it at 40, which we chose to balance the number of samples excluded with their closeness in PC-space. *aberrant* works only in two dimensions, so we applied it separately to pairs of PCs: 1&2; 3&4; 5&6 to find three sets of tightly clustered samples. We then took the intersection of all three sets, and defined this set of individuals as the "white British ancestry subset" (see Figure S8).

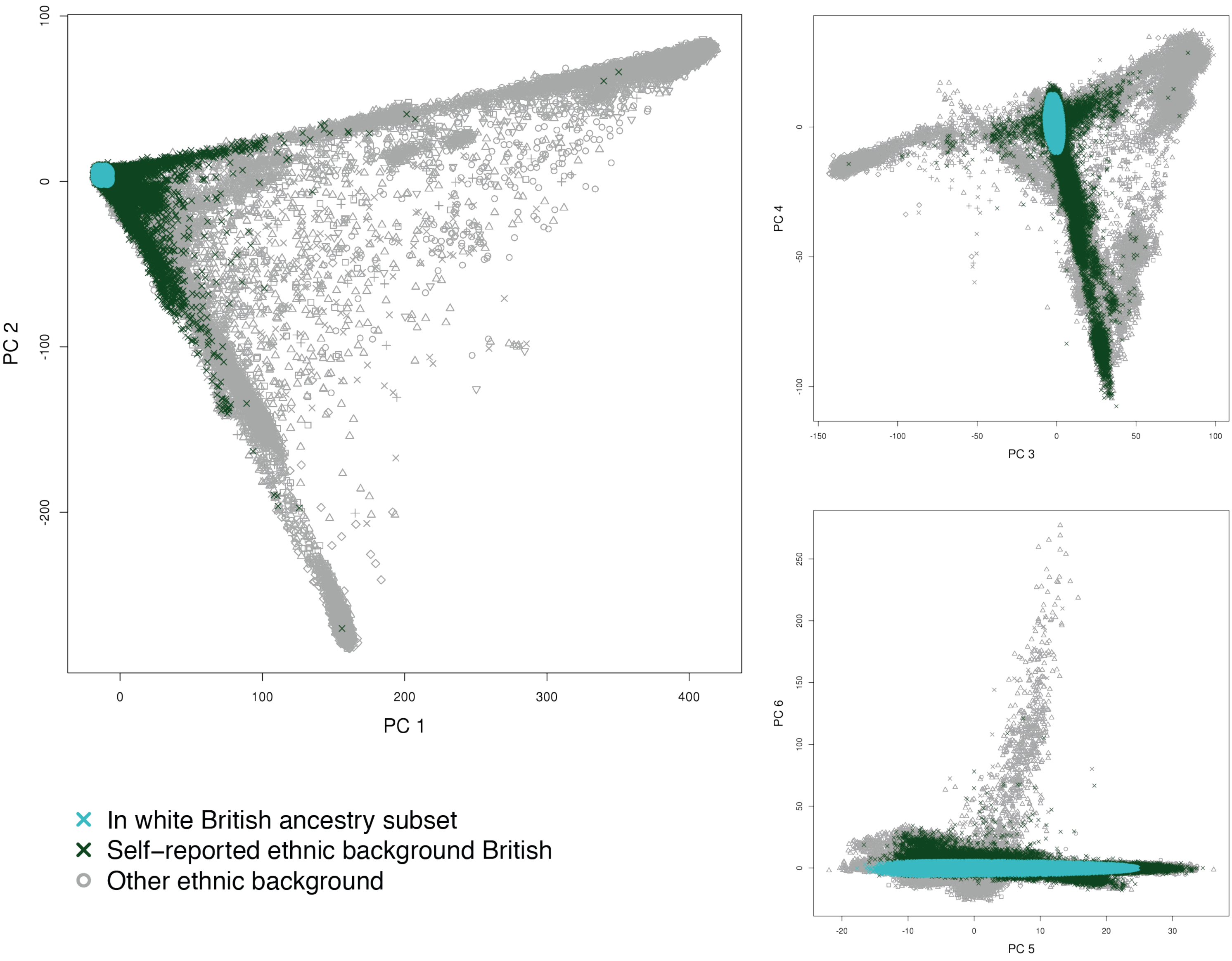


**Figure S8| Selection of white British ancestry subset using PCA.** Each plot shows the principal component scores for all UK Biobank samples (n=488,377 samples), which we used to select the white British ancestry subset (see Section S 3.4). Non-grey points indicate participants who have self-reported ethnic background "British" (within the broader-level group "White", see Extended Data Table 3), and participants with other ethnic backgrounds are coloured grey, but with the same set of symbols as shown in Figure S6. Blue crosses show participants within the white British ancestry subset.

## S 3.5 Detecting outliers in heterozygosity and missing rates

Extreme heterozygosity and/or high missing rates can be indicators of poor sample quality due to, for example, DNA contamination[18]. However, heterozygosity can also be sensitive to natural phenomena, including population structure, recent admixture, and parental consanguinity. We identified poor quality samples using these metrics, but took extra measures to avoid misclassifying good quality samples because of these effects.

### S 3.5.1 Details of computing raw heterozygosity and missing rates

Using a set of 605,876 high quality autosomal SNPs (see Section S 3.2) we computed raw heterozygosity ($h$) for each sample. That is, the proportion of non-missing genotypes that are heterozygous:

$$h = \frac{N_{nm} - N_{hom}}{N_{nm}}$$

where $N_{nm}$ is the number of non-missing genotypes, and $N_{hom}$ the number of homozygous genotypes, both computed using the "--het" command in *plink*. We computed missing rates using the "--miss" command in *plink*.

### S 3.5.2 Details of adjusting heterozygosity for population structure

The proportion of a sample's non-missing genotypes that are heterozygous (heterozygosity rate) is sensitive to population structure because allele frequency distributions (and thus expected heterozygosity) can differ between populations, especially in array-based genotype data. Extended Data Figure 1a shows the effect of SNP ascertainment on heterozygosity. We control for this by fitting the following linear regression model.

Let $h$ denote the heterozygosity and let $x$ be a set of features correlated with ancestry. We used the projections onto the six major UK Biobank principal components to characterise ancestry, writing $x = (x_1, x_2, x_3, x_4, x_5, x_6)$ for these six principal component values. Consider the following model for heterozygosity under population structure:

$$h(x) = h_0 + \beta(x)$$

where $h(x)$ is the raw heterozygosity, which depends on the features $x$, $h_0$ is the ancestry-adjusted heterozygosity, and $\beta(x)$ is a bias term due to population structure. We chose a quadratic form for $\beta(x)$, which includes all linear and quadratic terms $x_i$ and $x_i^2$ as well as all cross terms $x_ix_j$, and we estimated $h_0$ with ordinary least squares. More specifically, the bias was assumed to have the following functional form:

$$\beta(x) = \beta_1x_1 + \beta_2x_2 + \beta_3x_3 + \beta_4x_4 + \beta_5x_5 + \beta_6x_6 + \beta_{11}x_1^2 + \beta_{22}x_2^2 + \beta_{33}x_3^2 + \beta_{44}x_4^2 + \beta_{55}x_5^2 + \beta_{66}x_6^2 + \beta_{12}x_1x_2 + \beta_{13}x_1x_3 + \beta_{14}x_1x_4 + \beta_{15}x_1x_5 + \beta_{16}x_1x_6 + \beta_{23}x_2x_3 + \beta_{24}x_2x_4 + \beta_{25}x_2x_5 + \beta_{26}x_2x_6 + \beta_{34}x_3x_4 + \beta_{35}x_3x_5 + \beta_{36}x_3x_6 + \beta_{45}x_4x_5 + \beta_{46}x_4x_6 + \beta_{56}x_5x_6$$

The fitted value $\hat{h}_0$ is the ancestry-corrected heterozygosity. We plot this on the *y*-axis in Extended Data Figure 1b with all ethnic background categories combined, and in Figure S9 with each ethnic background category separately. Both the PC-corrected and raw heterozygosity are provided to researchers.

**S 3.5.3 Details of detecting outliers in heterozygosity and missing rates**

Some samples can have naturally extreme heterozygosity, even after accounting for population structure. Specifically, individuals with mixed ethnicity tend to have higher heterozygosity (which is not captured by the principal components), and individuals whose parents are closely related tend to have lower heterozygosity. We therefore aimed to flag as outliers samples whose extreme heterozygosity is not explained by mixed ancestry or increased levels of consanguinity. We proceeded as follows, with missing rates and heterozygosity computed as described above.

We first considered individuals within the four largest ethnic background categories ("British", "Any other white background", "Irish", "Indian"). To this combined set we applied *aberrant*[10] to the two-dimensions of logit-transformed missing rate and PC-adjusted heterozygosity (*lambda* = 120). We used the logit transformation of missing rate because *aberrant* uses a model of a mixture of 2-dimensional Normal distributions, and the missing rate distribution is approximately normal under this transformation. In this way we identified 744 outliers and with PC-adjusted heterozygosity above the mean (0.1903). For all other ethnic background categories we inspected plots of missing rate and PC-adjusted heterozygosity separately for each category, looking for individuals with unusually high heterozygosity within their category (Figure S9). This resulted in zero further outliers. We computed missing rates using plink "--miss" command, and also flagged any sample with a missing rate > 0.05. In total we identified 968 samples with unusually high heterozygosity and/or missing rates. These samples are shown in red in Extended Data Figure 1c.

Low heterozygosity is expected as a consequence of an individual's parents sharing recent ancestors. This would also result in long runs of unbroken homozygous genotypes within the individual's genome. We used this observation to confirm that individuals with unusually low heterozygosity were not subject to poor quality genotyping by checking the expected (negative proportional) relationship between heterozygosity and long runs of homozygosity (LROH). We used *plink*[9] to detect LROH, using the "--homozyg-kb" command with a homozygous run required to span at least 1000 kb. The negative relationship between the heterozygosity and the total length of all runs of homozygosity is clear in Supplementary Figure S10.

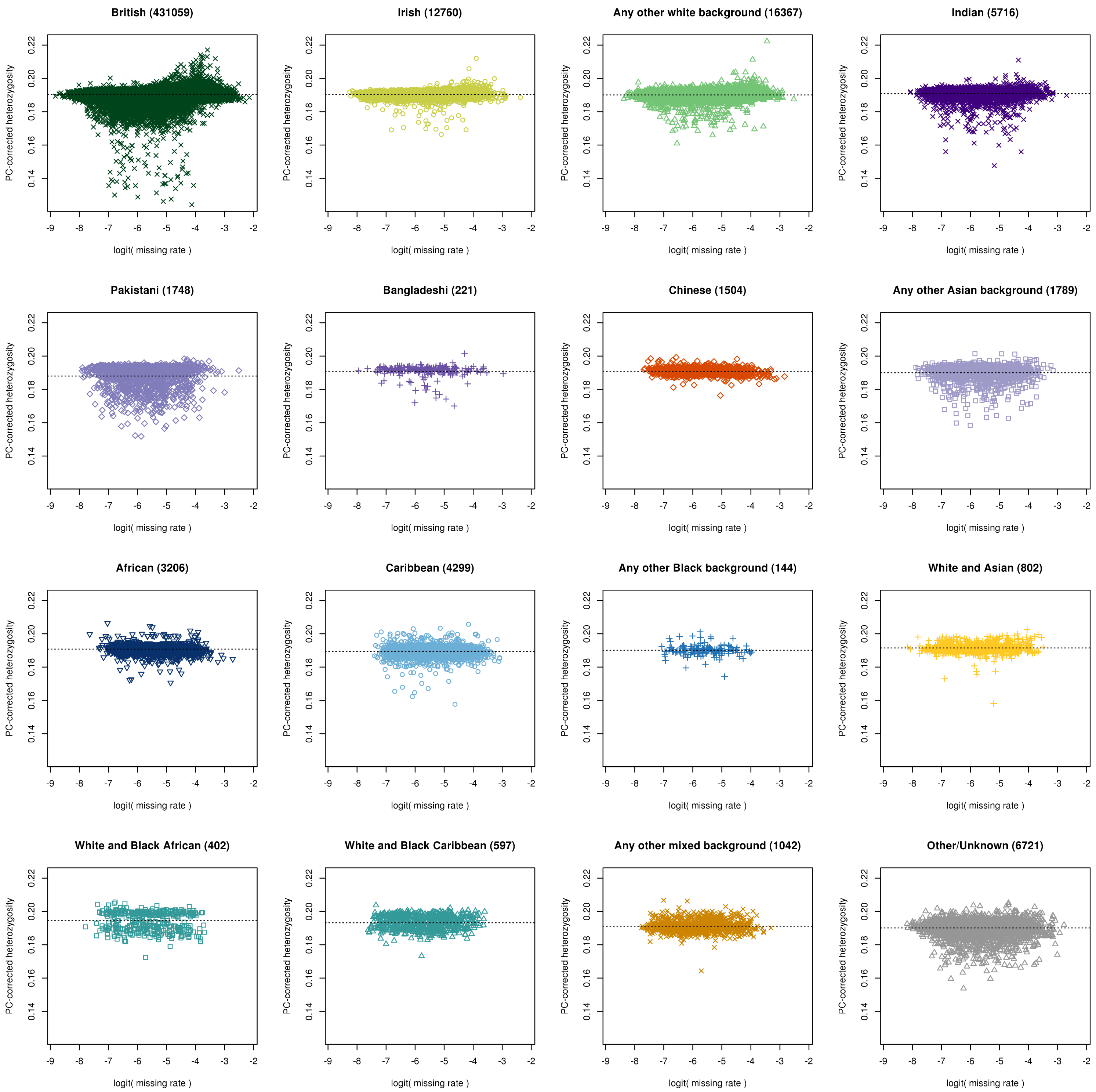


**Figure S8 | PC-corrected heterozygosity and missing rates for different ethnic background categories.** Horizontal lines show the mean value within each group. The number of samples is shown in brackets. Groups with mixed ancestry (e.g. White and Black African) tend to have higher heterozygosity even after correcting for PCs. We therefore only included the largest ethnic background categories (shown in the top four plots) in the automated outlier detection process and for the other ethnic background categories we visually inspected these plots (Section S 3.5.3).

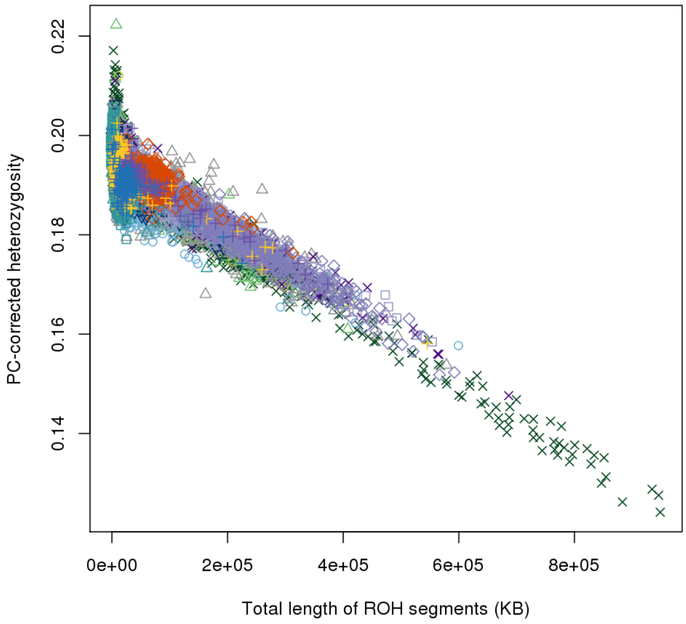


**Figure S9 | Observed relationship between long runs of homozygosity and heterozygosity.** The colours and symbols show self-reported ethnic background as in Figure S9 (n=488,377 samples).

# S 3.6 Sex chromosome-specific sample QC

Affymetrix infers the sex of each individual based on the relative intensity of markers on the Y and X chromosomes[13]. Sex is also reported by participants, and we refer to differences between these sources as a 'sex mismatch'. This could occur because of sample mishandling, but also due to due to transgender individuals, or instances of real (but rare) genetic variation, such as sex-chromosome aneuploidies.

Using information in the measured intensities of chromosomes X and Y, we identified a set of 652 individuals with sex chromosome karyotypes putatively different from XY or XX. The list is made available to researchers. These have not been independently validated, but rather the list is useful as a QC tool. For example, differentiating between sex-mismatches likely due to unusual chromosomal configurations, as opposed to other reasons such as clerical error. We also excluded this set of individuals from phasing and imputation on the X chromosome only, as assumptions about diploidy/haploidy in this chromosome may not hold for these individuals.

## S 3.6.1 Details of putative sex-chromosome aneuploidy detection

We first extracted Log2 ratios (L2R) at a set of high quality SNPs on the X and Y chromosomes (see Section S 3.2). L2R are computed (by Affymetrix) for each sample at each marker, and is the sum of the A and B allele intensities for the marker, normalized by the median intensity of that marker in individuals assumed to represent the normal copy number state at that site. For normalization, only individuals in the same genotyping batch were used; and for markers on the X and Y

chromosomes only (inferred) females and males, respectively, were used[19]. For each individual we computed the mean L2R across each of the sex-specific region of the X chromosome (*L2Rx*) and the Y chromosome (*L2Ry*). After visual examination of a scatter plot of these metrics (Figure 2d) 652 individuals were flagged based on the criteria in Table S2. The 225 samples with a possible XXY karyotype are heavily enriched for sex mismatches (79%). All but one of the XXY karyotype cases the submitted sex was male, which would be consistent with the occurrence of a Y chromosome, which contains the sex-determining region. Table S2 shows the rate in UK Biobank compared with data derived from a prospective study of 39,410 newborn children at a single hospital in Aarhus, Denmark[20]. UK Biobank is a much larger study, and may also differ in a number of respects through its ascertainment, including survival to age 40-69, and willingness or ability to participate in the study.

| Criteria | Putative sex chromosome karyotype | Sex match | Sex mismatch | Total | Rate per 10,000 | Sex-specific rates per 10,000 | |
|---|---|---|---|---|---|---|---|
| | | | | | | In UK Biobank (adults) | In Nielsen and Wohlert (new-borns) |
| **Inferred female and *L2Rx* < -0.17** | X0 (complete, or mosaic) | 148 | 2 | 150 | 3.07 | 5.67 (0.46) | 5.28 (1.76) |
| **Inferred female and *L2Rx* > 0.145** | XXX | 123 | 0 | 123 | 2.52 | 4.65 (0.42) | 10.56 (2.49) |
| **-1 >= *L2Ry* < 0.23 and *L2Rx* > -0.2** | XXY | 47 | 178 | 225 | 4.61 | 10.06 (0.67) | 11.75 (2.56) |
| ***L2Ry* >= 0.23** | XYY or XXYY | 153 | 1 | 154 | 3.15 | 6.89 (0.56) | 11.19 (2.50) |
| **Not any of the above** | XX or XY | 487528 | 197 | 487725 | 9986.65 | | |
| | Total | 487999 | 378 | 488377 | 10000 | | |

**Table S2 | Criteria used for identifying putative sex-chromosome aneuploidy in UK Biobank and comparison of rates.** Rates for Nielsen and Wohlert are derived from Table 1 of their publication[20], which was a prospective study of new-born children (17,872 boys and 17,038 girls). To match the reporting of that study we estimated sex-specific rates in the UK Biobank data using the total numbers of self-reported males (223,605) for XXY, XYY or XXYY and self-reported females (264,772) for X0, XXX. Numbers in brackets after rates are the standard errors of the estimate.

## S 3.7 Inference of familial relatedness

Close relationships (e.g. siblings) among UK Biobank participants were not recorded during the collection of other phenotypic information. Indeed, many participants may not be aware that a relation (such as an aunt, or sibling) is also part of the cohort. However, this information is important for epidemiological analyses, as well

as in GWAS, and the genetic data provide a unique opportunity to discover and characterise familial relatedness within the cohort. This analysis is also useful for identifying samples that are experimental duplicates rather than genuine twins.

We identified related individuals by estimating kinship coefficients for all pairs of samples, and recorded coefficients for pairs of relatives who were inferred to be 3$^{rd}$ degree or closer. The kinship coefficient is the probability that two alleles sampled randomly from two individuals are identical by descent. Expected kinship coefficients decrease by a multiple of 1/2 for each degree of relatedness. For example, parent-offspring pairs (1$^{st}$ degree relatives) have an expected kinship coefficient of 1/4, and grandparent-grandchild pairs (2$^{nd}$ degree relatives) have an expected kinship coefficient of 1/8. Variation around the expected values is a result of the stochastic nature of genetic inheritance or other effects such as parental consanguinity.

Kinship coefficient estimation in a large and diverse cohort presents unique challenges. Specifically: diverse ancestral backgrounds, recent admixture, and computational scalability. As such, we used an estimator implemented in the software, *KING*[16], as it is robust to population structure (i.e. does not rely on accurate estimates of population allele frequencies) and it is implemented in an algorithm efficient enough to consider all $\sim 1.20 \times 10^{11}$ pairs in a practicable amount of time.

### S 3.7.1 Details of relatedness inference

We estimated kinship coefficients for pairs of individuals using the following procedure. We first selected a set of SNPs that are only weakly informative of ancestry to minimise inflation of the kinship estimates due to recent admixture. Using results of the PCA round 1 (see Section S 3.3) we selected SNPs that only contribute very small 'loads' to PCs 1-3. That is, where $t_k$ is the value of SNP-load for PC $k$, we only used SNPs with $t_k < 0.003$ for all $k$ in 1,2,3. The threshold was chosen to balance the number of SNPs included (too few would lead to noisy kinship estimates), and how informative of ancestry the SNPs are (too large a threshold would lead to inflation in the presence of recent admixture). This resulted in a set of 93,511 SNPs to use for the final kinship inference. The affect this had on the kinship estimates is shown in Figure S11 and Figure S12. We also excluded individuals in the list of outliers in heterozygosity and missing rates (see Section S 3.5).

With the genotypes filtered as described above, we computed kinship coefficients for all pairs of individuals using *KING* and recorded the pairs of degree 3 or closer (kinship coefficient $\geq 1/2^{(9/2)}$ )[16]. In practice, we parallelised this computation by combining data into pairs of batches ("--merge" command in *plink*) and running *KING* with the options "--related --degree 3" on all pairs of batches. We then merged the results into one pairwise kinship table.

A small number of individuals (9) appeared to be related (3$^{rd}$ degree) to a very large number (> 200) of individuals. In some cases this was in the order of 1000s, and their 'relatives' were usually not themselves related to one another. By considering

family trees, it is only possible for an individual to have a maximum of four 3rd degree relatives who are not themselves related. These individuals also had slightly elevated heterozygosity and missing rates, but not extreme enough to flag as poor quality (see Section S 3.5). We therefore concluded that the excess related pairs are likely to be false positives, and being driven by a small number of individuals, so we excluded them from the kinship table. These 9 individuals, along with the pre-filtered samples, comprise a set of 977 samples that are effectively excluded from the kinship inference. For this small fraction (0.2%) of the cohort we therefore cannot confirm the presence or absence of any of their relatives in the cohort. A list of these individuals is provided to researchers.

The output of *KING* (after the filtering described above) is provided to researchers in a table, which contains 107,162 pairs of individuals, involving 147,731 unique individuals. For each pair we report both the estimated kinship coefficient and the fraction of markers for which they share no alleles (IBS0). For the purposes of this paper we also called the relationship class of each pair (Extended Data Table 5 and Figure 3b-c). That is, we assigned each related pair to one of twins, parent-offspring, siblings, 2nd degree or 3rd degree relatives using the kinship coefficient boundaries recommended by the authors of *KING* (see Table 1 in their publication[16]). We used IBS0 only to distinguish parent-child from sibling pairs, who have the same expected kinship coefficient. Specifically, we called any pair with IBS0 < 0.0012 as parent-offspring.

We found large networks of related individuals, such as those shown in Figure 3b, using the "cluster" function in the *igraph* (v1.0.1) package[21] in *R*. It should be noted that the size of these networks may change if participants withdraw from the resource.

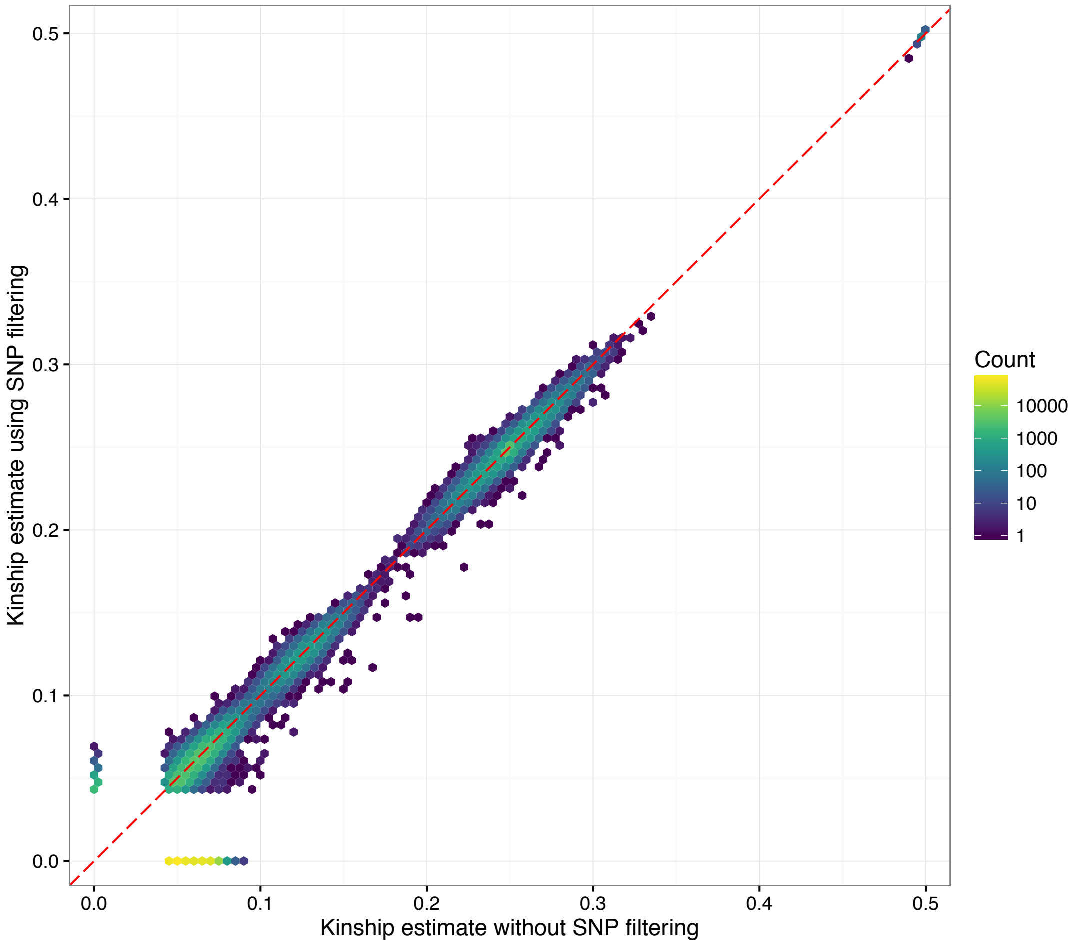


**Figure S10 I Kinship coefficient estimates before and after filtering SNPs.** On each axis are the kinship coefficients of pairs of samples inferred to be 3rd degree relatives or closer in either of the two analyses using KING. Colours indicate the number of pairs that fall within the range of each hexagonal bin (436,359 pairs in total). Most of the pairs that changed relationship class as a result of the SNP filtering were those that shifted from "3rd degree" to unrelated (yellow/green hexagons in the bottom left).

**A Effect of recent admixture on kinship coefficient estimation before applying SNP filters**

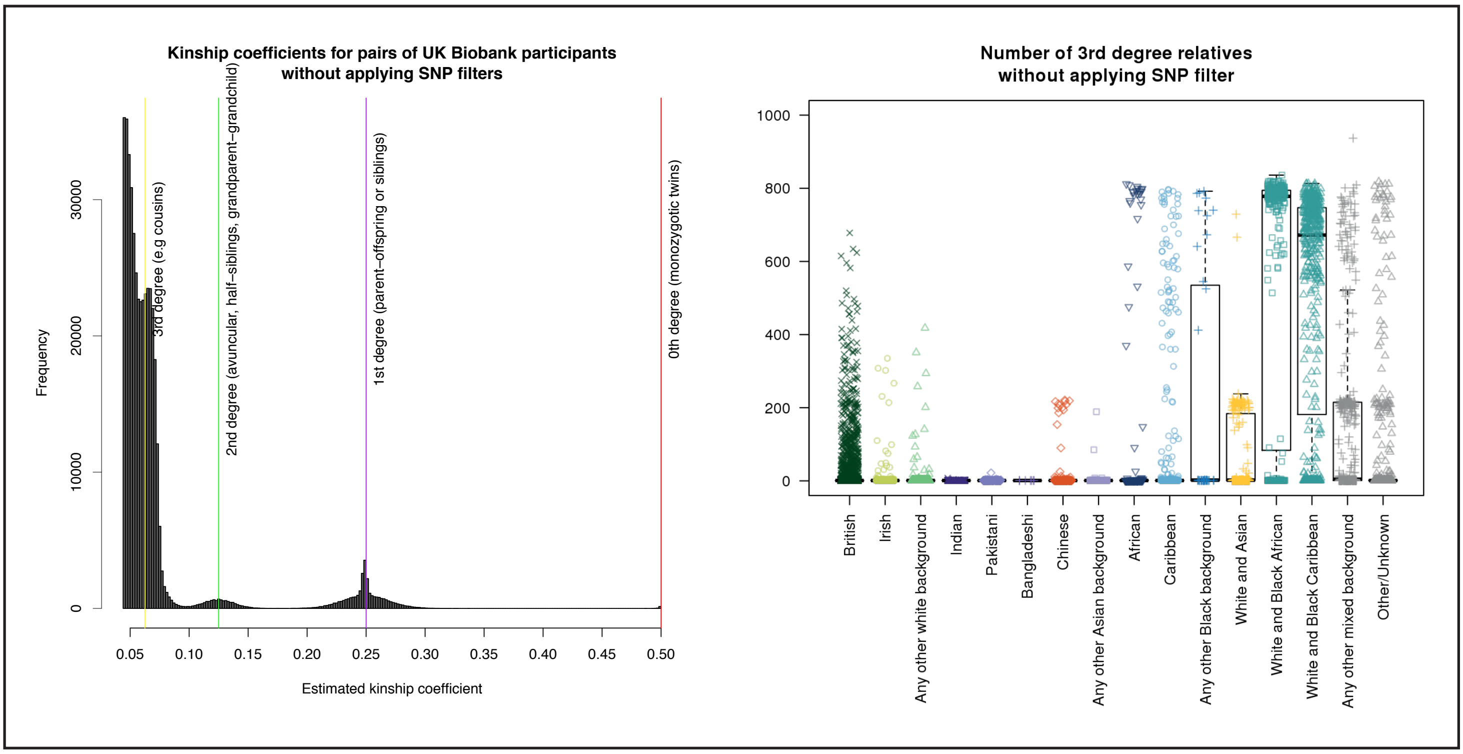


**B Kinship coefficient estimation after applying SNP filters**

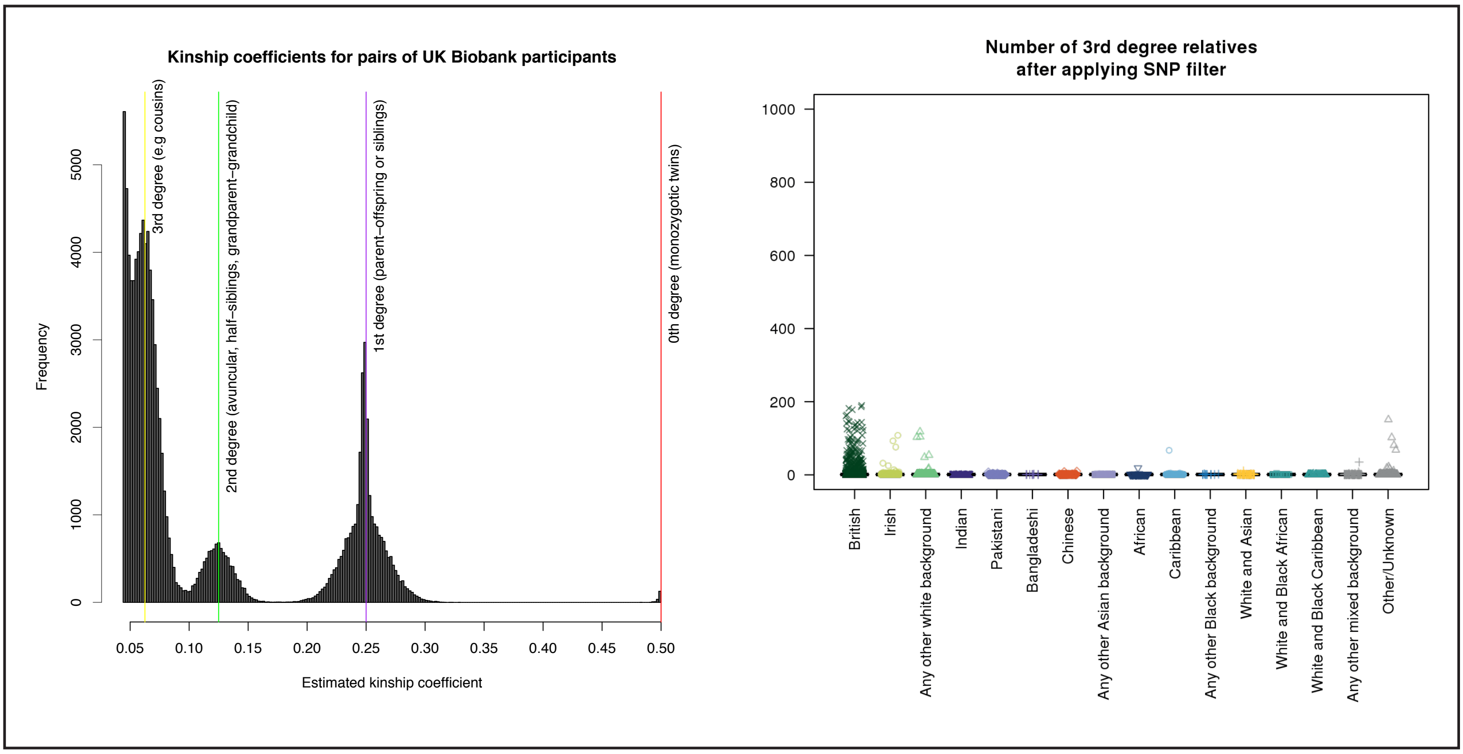


**Figure S11 | The effect of PC-based SNP filtering on kinship coefficient estimation.** Both sub-figures show a histogram (on the left) of kinship coefficients estimated by KING using a 3$^{rd}$ degree cut-off, with vertical lines placed at the expected coefficients for different degrees of relatedness. The box plots on the right show the distribution of the number of 3$^{rd}$ degree relatives inferred for each sample (excluding zero) within each ethnic group. **A)** Plots based on kinship coefficient estimates using a set of autosomal SNPs selected for genotyping quality (see Section S 3.2). In the histogram (432,379 pairs) the excess of 3$^{rd}$ degree pairs is evident, and the boxplot shows how the ethnic groups involving mixed ancestry are disproportionately affected (n=151,130 individuals). The accumulation of points around ~800 and ~200 relatives occurs because there are two sets of samples in which almost all pairs are 'related' to each other. Namely, those with ethnic backgrounds originating in Africa+Europe and Asia+Europe. **B)** Plots based on kinship coefficient estimates after excluding SNPs informative of ancestry based on PCA (see Section S 3.7.1). The reduction in an excess of 3$^{rd}$ degree relatives among mixed ancestry ethnic groups is clear from the box plot. The histogram shows 107,162 pairs, and the boxplot shows 147731 individuals.

### S 3.7.2 Details of kinship validation

We validated our kinship estimates by applying a different kinship coefficient estimator based on allele frequencies, implemented in *plink*'s "--genome" command [9]. We used the same set of LD-pruned SNPs as with the *KING* analysis, and the option "--min" to apply the same cut-off for 3rd degree ( $2 \times 1/2^{(9/2)} = 0.08838835$ ). The multiple of 2 accounts for the fact that *plink* actually estimates the IBD-sharing fraction which is, by definition, twice the kinship coefficient[16]. In order to avoid population structure effects we restricted the analysis to pairs of related individuals (according to the *KING* analysis detailed above) where both are in the white British ancestry subset (see Section S 3.4). These account for 85% of all the inferred pairs.

Of all the pairs of relatives in the subset 99.9% were confirmed as 3rd degree or closer using *plink*. The small fraction of unconfirmed pairs all had a kinship coefficient (according to *KING*) smaller than 0.0486, which is close to the cut-off between 3rd and 4th degree. Furthermore, all twins, parent-offspring and sibling pairs were confirmed as having the same degree of relatedness in the *plink* analysis. There was some discrepancy between the assignment of 2nd and 3rd degree relatives. A number (5%) of 3rd degree pairs from KING were called as 2nd degree pairs in the *plink* analysis, although *plink* also inferred a much larger number ($10^7$) of 3rd degree pairs which is unrealistic for this dataset.

### S 3.7.3 Details of distinguishing identical twins from duplicated samples

Without considering phenotype information, a pair of duplicated samples in the genotype data will be indistinguishable from genuine identical twins because they will all have kinship coefficient 0.5. To resolve this, UK Biobank staff reviewed phenotype details of a list of 894 candidate pairs of samples (all those with kinship coefficient close to 0.5). Where evidence was found that the participants may be twins, triplets, or part of a multiple birth, the pair was marked as twins (188 pairs). Any remaining pairs were marked as either "Blind Spike Duplicates" (588 pairs) or "unintended" duplicates (118 pairs). See S1.4.1 for details of "Blind Spike Duplicates". Unintended duplicates were pairs of samples that had not been included as Blind Spike Duplicates, and were associated with phenotype information from different participants who were not identical twins. Genotype call concordance rates for the 588 Blind Spike Duplicate pairs are shown in Figure S13.

A total of 1,364 samples were identified as duplicates, some duplicated more than once. We excluded 793 of these from the released genotype data. That is, we excluded all samples within the unintended duplicate pairs because for these samples the correct link between the genotype data and phenotype information cannot be guaranteed; and we kept a single sample from each of the Blind Spike Duplicates (the one with the highest call rate).

The total number of twins reported in the released kinship table is 179, not 188 (Extended Data Table 5). This is due to a pair of confirmed twins, whose samples were also duplicated three times each, making 9 pairs of 'twins'. We kept data for just one instance of this pair. One further pair of confirmed twins involved a sample that was part of the 977 that were excluded from the kinship table due to quality issues (see Section S 3.7.1).

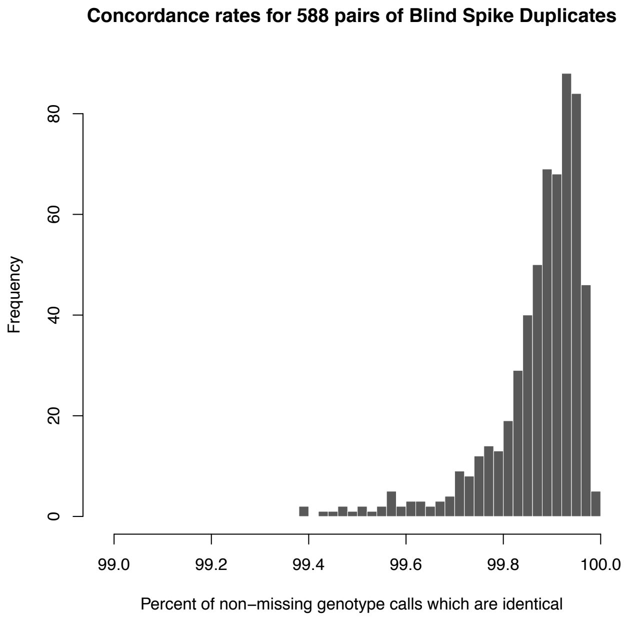


**Figure S12 | Concordance rates for Blind Spike Duplicates.** For each Blind Spike Duplicate pair (two genotyped samples from the same individual) we calculated the fraction of markers with the same genotype call in both samples, excluding any markers missing in one or both samples.

## S 3.7.4 Details of finding a maximal set of unrelated individuals

We used the following procedure for finding a maximal set of unrelated individuals among a set of quality-filtered samples (for example, for PCA, Section S 3.3). That is, the largest possible subset of unrelated individuals, and for which there are many possible solutions. An unrelated individual is one with no relative 3$^{rd}$ degree or closer in our analysis. We first pruned the full pairwise kinship table so that it only contained individuals in the set of interest. Using the *igraph* (v1.0.1) package[21] in *R* we then converted the table into a graph object, where each vertex is an individual, and edges exist between pairs of related individuals. Then, for each 'family' (i.e. a network of nodes joined by edges), we found the largest subset of individuals (vertices) such that there is no relatedness (edges) between them. In the case of trios, for example, the child would be excluded, leaving the two unrelated parents. We used an algorithm implemented in the "largest_ivs" function in *igraph.* When there was a choice of solutions (e.g. within a set of 3 siblings), one solution was chosen at random.

## S 3.7.5 Resolving relationships in networks of 2$^{nd}$-degree relatives

Here we show that any set of individuals that are all 2$^{nd}$-degree relatives of each other contains at most one individual who is not a half-sibling of all the others. Thus,

the network of 11 2nd-degree relatives in the UK Biobank (Figure 3c) contain at least 10 half-siblings with a shared parent.

We first assume that parents of individuals are unrelated, and that pairs of 2nd-degree relatives are either half-siblings, aunt(uncle)/niece(nephew), or grandparent/grandchild. Under these assumptions, there are only three possible configurations of a 3-person network where all three are 2nd-degree relatives of each other, as illustrated in Figure S14, $k$=3. Any other configuration requires that at least one pair is not a 2nd-degree relative. For example, if one pair is avuncular (uncle and nephew, say), and the third person is an aunt of the nephew, then she is either unrelated to the uncle, or she is his full sibling. If we add a fourth person to the network and apply the same 3-person rules as before, then we are left with only three possibilities (Figure S14, $k$=4). That is, there is at most one individual who is not a half-sibling of all the others. Note that all three can be formed starting from 3A, but only 4B can be formed from 3B, and only 4C can be formed from 3C.

Call the set of individuals that are all half-siblings of each other $H$ (blue dots in Figure S14*,* and all the other individuals $J$ (black dots in Figure S14). For $k$=3, it is true that there is at most one individual who is either an uncle/aunt or grandparent of all the others, and the rest are half-siblings. That is, size($J$) <= 1. We next show by induction, that this must be true for any $k$>2.

Consider a network of $k$ individuals all related 2nd-degree to each other, where size($J_k$) = 1 or size($J_k$) = 0. If we add an additional individual, $m$, who is related 2nd-degree to all $k$ individuals, we can show that size($J_{k+1}$) is still at most 1.

For the case where size($J_k$) = 1:

Consider a 4-person set formed by any half-sibling pair in $H_k$, the single individual in $J_k$, and the additional individual $m$. The only allowable configurations are shown in 4B and 4C. That is, those where individual $m$ is also a half-sibling of the existing pair in $H_k$. This is true for all the 4-person sets as defined above, so $m$ must be in the set $H_{k+1}$, and thus size($J_{k+1}$)=1.

For the case where size($J_k$) = 0:

There are three possible configurations for the 3-person set formed by any half-sibling pair in $H_k$, and the individual $m$ (shown in Figure S14).

- If the set has configuration (3A), then any 4-person set involving this plus one of the other individuals in $H_k$ must be in configuration (4A). Thus, the individual $m$ must be half-siblings with everyone in $H_k$, so size($J_{k+1}$)=0.
- If the set has configuration (3B), individual $m$ must be the aunt/uncle (since the other two are in $H_k$). Then, any 4-person set involving this plus one of the other individuals in $H_k$ must be in configuration (4B). Thus, individual $m$ is an aunt/uncle of everyone in $H_k$, so size($J_{k+1}$)=1.

- If the set has configuration is (3C), by the same argument as above (replace 3B and 4B with 3C and 4C) individual *m* must be a grandparent of everyone in $H_k$, so size($J_{k+1}$)=1.

Thus, size($J_{k+1}$)<=1 in all cases.

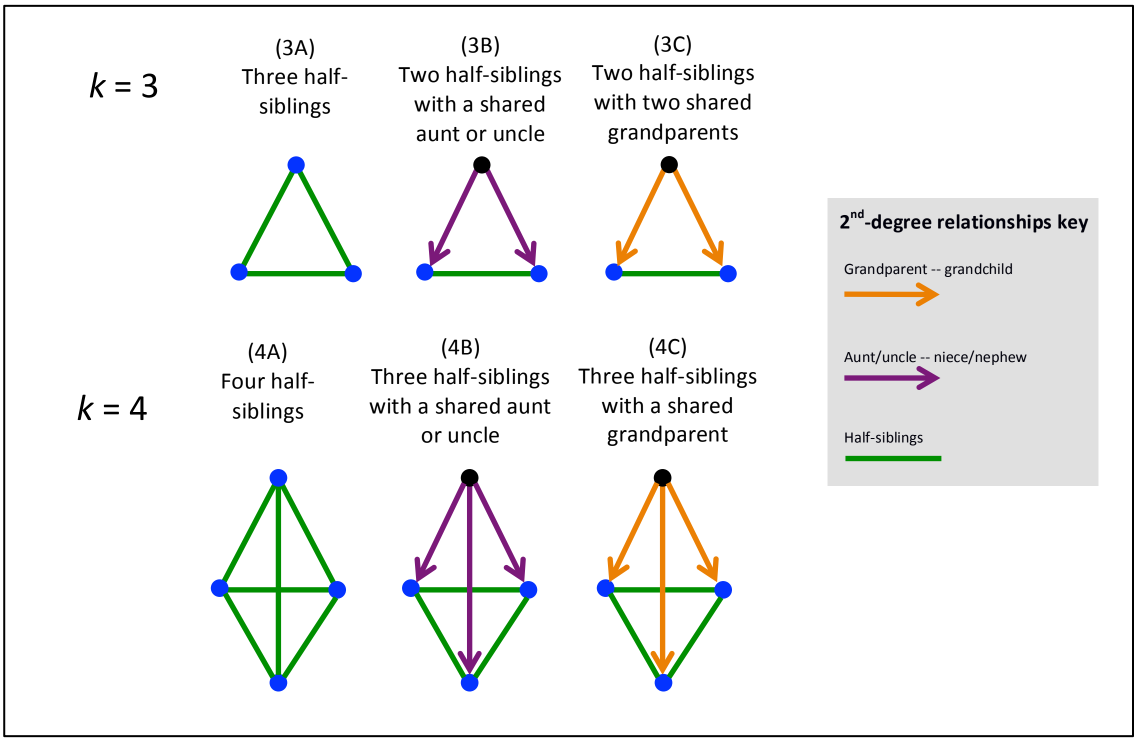


**Figure S14 | All possible configurations of sets of relatives that are all related 2nd-degree, for set sizes 3 and 4.** Coloured lines distinguish the different relationships between individuals (dots). Arrows indicate the direction of inheritance for grand-parent/grandchild and avuncular relationships as shown in the key.

**S 3.7.6 Estimating the theoretical expectation of the number of related pairs in the cohort**

We estimated the theoretical expected number of sibling or cousin pairs in a simple random sample of the population eligible for UK Biobank. To do this we derived the following equations with parameter definitions shown in Table S3.

Expected number of pairs within a sample:

= $Pr$(Sampling a cousin pair)x(Number of pairs in sample)

$$= \frac{2\mu_1(\hat{\mu}_0 - 1)}{N-1}\frac{n(n-1)}{2}$$
$$= \frac{\mu_1(\hat{\mu}_0 - 1)n(n-1)}{N-1}$$

Expected number of sibling pairs in sample:

= $Pr$(Sampling a sibling pair)x(Number of pairs in sample)

$$= \frac{(\hat{\mu}_1 - 1)}{N-1}\frac{n(n-1)}{2}$$

We derived these equations based on the following assumptions:

- Most eligible individuals are descendants of UK citizens so we can apply historical UK fertility rates.
- The vast majority of 3$^{rd}$ degree pairs in the cohort are cousins (as opposed to great-uncle/aunts; or connections involving half-sibs). This is likely to be true given that the age-range only spans about one human generation.
- The sample size (*n*) is small compared to the population size (*N*) so that sampling with replacement can be assumed.

Several factors make estimating these values challenging. Fertility rates of mothers that were having children during the time of the birth years of this cohort (1938 - 1968) changed dramatically (Office of National Statistics, UK). Therefore, the mean family size is likely to depend on the birth-year of the mothers of individuals in the cohort. Secondly, the age-distribution of women bearing children also changed over this time, affecting the likely birth-year of the mothers. Instead of modeling these factors directly, we simply computed a maximum and minimum expected value, based on the maximum and minimum observed fertility rates for mothers in the time of the birth years of this cohort. The estimates also depend on the sampling fraction (*n/N*), which is different for different age-groups in the cohort (people aged 60-70 are over-represented, and people aged 40-44 are under-represented[22]). To account for this we computed the estimate separately for 5-year age-groups and summed the results. Table S4 shows the expected and observed numbers of pairs in the UK Biobank, using the parameters shown in Table S3. There are about 2 times as many sibling pairs, and between 1.2 and 2 times as many cousin pairs as theoretically predicted under simple random sampling.

| Parameter | Description | Range(s) | Source |
|---|---|---|---|
| $N$ | Total population size eligible for the UK Biobank sample. | Total: 21,734,300 | 2006 mid-year population estimate for people aged 40-69 (Office of National Statistics, UK) |
| $n$ | Size of UK Biobank sample (and successfully genotyped). | Total: 488,410 | UK Biobank |
| $\mu_1$ | Average completed family size in sampled generation (includes childless mothers). Counts expected number of children per aunt/uncle. | [1.91, 2.42] | Completed cohort fertility for women born between 1920 and 1953 (Office of National Statistics, UK). |
| $\hat{\mu}_1$ | Average completed family size in sampled generation (excludes childless mothers). Counts expected number of siblings. | [2.301, 2.75] | Completed cohort fertility for women born between 1920 and 1953 and have given birth to at least one child (Office of National Statistics, UK). |
| $\hat{\mu}_0$ | Average completed family size in previous generation (excludes childless mothers). Counts expected number of aunts/uncles. | [2.301, 2.75] | No direct data is available on fertility rates of women born before 1920 so assume the range is similar to $\hat{\mu}_1$ . |

**Table S3 | Parameters for estimating number of expected sibling or cousin pairs in UK Biobank cohort.**

| | Expected number of pairs (range*) | Observed number of pairs |
|---|---|---|
| **1st degree sibling pairs** | 9,530 – 11,110 | 22,667 |
| **3rd degree pairs (cousins)** | 36,390 – 53,790 | 66,935 |

**Table S4 | Expected and observed numbers of pairs of related individuals in the UK Biobank cohort.** Expected ranges are based on using the minimum (and maximum) parameters for family sizes given in Table S3.

# S 4 Assessment of the UK Biobank Array for imputation

The UK Biobank Axiom array from Affymetrix was specifically designed to optimize imputation performance in GWAS studies[3]. An experiment was carried out to assess the imputation performance of the array, stratified by allele frequency, and to compare performance to a range of old and new commercially available arrays. In such an experiment it is desirable to use validation data on an independent set of samples. Therefore performance was assessed using high-coverage, whole-genome sequence data made publicly available by Complete Genomics (CG) (http://ftp.1000genomes.ebi.ac.uk/vol1/ftp/technical/working/20130524_cgi_combined_calls/). Data from 10 samples from the European ancestry (CEU) population were used. All markers with a call rate below 90% were filtered out in order to only consider very reliable sites in the analysis. Only data from chromosome 20 were used.

To mimic a typical imputation analysis, a pseudo-GWAS dataset was constructed by extracting the CG marker genotypes at all the sites included on a given array. All sites not on the array were then imputed using either the HRC reference panel [23] or the UK10K panel[24]. We used both these panels because both were used to impute the UK Biobank dataset. This experiment was repeated for 10 different genome-wide SNP arrays (i) Applied Biosystems UK Biobank Axiom, (ii) Illumina 1M-Duo3_C, (iii) Illumina HumanOmni5-4v1 (iv) Illumina HumanCoreExome-12v1 (v) Illumina Global Screening Array (vi) Illumina HumanHap300_v2 (vii) Illumina HumanHap550_v3 (viii) Illumina HumanOmni2.5-8v1 (ix) Illumina Multi-Ethnic Global Array (x) Affymetrix GenomeWideSNP_6.

Markers were stratified into allele frequency bins and the squared correlation ($R^2$) was calculated between the allele dosages at variants in each bin with the masked CG genotypes. Since different arrays contain different numbers of variants it is important to make sure that imputation performance is measured at the same set of variants when comparing chips. To achieve this, both imputed and array variants were included in the $R^2$ analysis, so that the comparison measures the *overall performance* of each array. As a consequence, an array with more variants will gain an advantage, as it is reasonable to expect that directly genotyping a variant will yield more accurate genotypes than imputation. Figure S15 shows the results of this analysis for the (A) HRC, and (B) UK10K reference panels. The x-axis is non-reference allele frequency (%) on a log scale, which focuses in on rarer variants. The y-axis is imputation performance ($R^2$).

The UK Biobank Axiom array (pink line) compares very well to other arrays. Specifically, this array shows very similar performance to the Illumina HumanOmni2.5 array (bright green line) which has ~3 times the number of SNPs. It is worth noting that the UK Biobank Axiom array is slightly better that the Illumina Omni 2.5M chip in the 1-5% range. This is a likely consequence of the array design process focusing in part on this frequency range. Another notable point is that the

Illumina Global Screening array (orange line) seems to have non-optimal performance on common SNPs. Above 5% frequency this array seems to perform as well as the Illumina HumanHap300 array, which is one of the oldest arrays in this comparison.

The overall conclusion of this analysis is that the UK Biobank Axiom array is a very good array from which to carry out genotype imputation. The caveat is that this analysis is focused on samples with European ancestry.

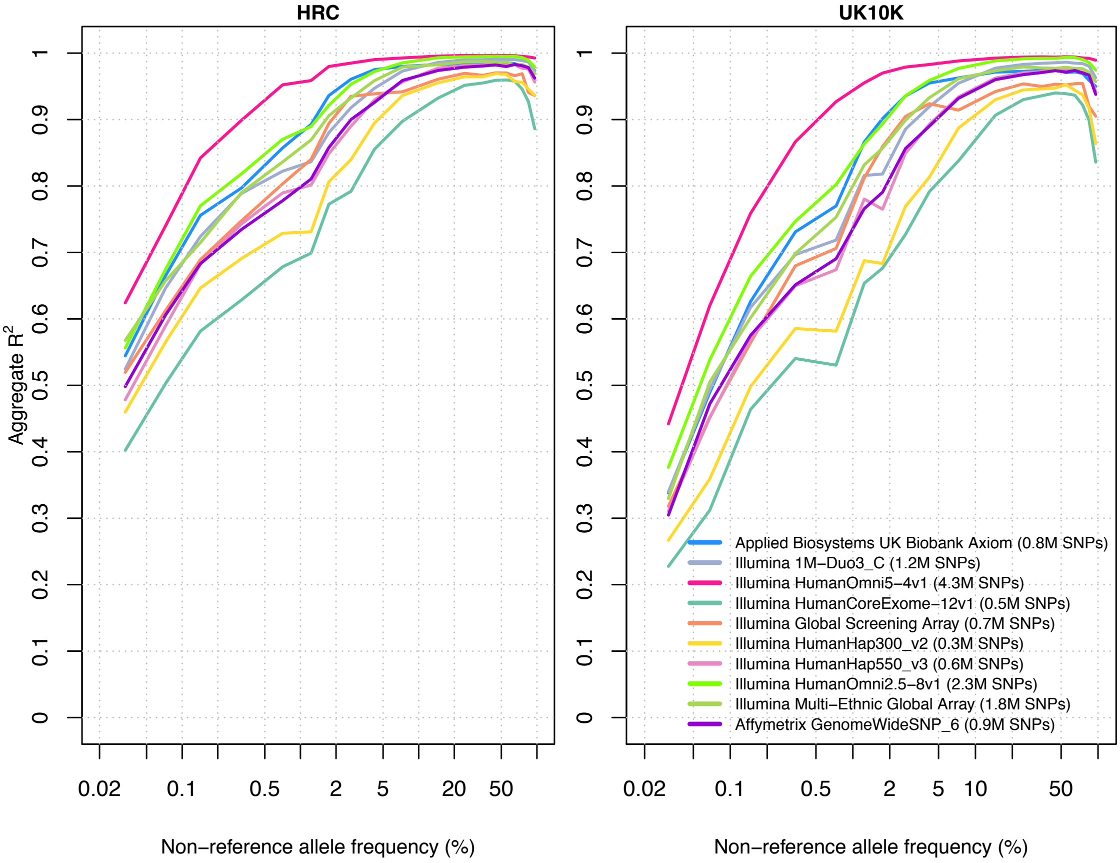


**Figure S15 | Comparison of imputation performance of the UK Biobank Axiom array and several other commercially available genotyping arrays.** The x-axis of each plot shows non-reference allele frequency on a log-scale, which accentuates low allele frequencies. The y-axis shows imputation performance in terms of $R^2$.

# S 5 Imputation of classical HLA alleles

We imputed 11 loci in the MHC corresponding to the genes *HLA-A, -B, -C, -DRB5, -DRB4, -DRB3, -DRB1, -DQB1, -DQA1, -DPB1* and *-DPA1*. Imputation models were constructed using markers genotyped in both the reference and UK Biobank datasets and within 1 Mb either side of the HLA locus. Because the extent of laboratory-based typing differed for each of the reference cohorts, we created separate reference panels for each locus in order to retain the maximal number of markers. Specifically, for each locus we included only the individuals which had laboratory-derived HLA types for that locus, and only the markers that were polymorphic and were typed in at least 98% of that set of individuals (Table S6). For each locus and chromosome we reported the allele with the highest posterior probability.

Association analysis was performed with a set of disease terms derived from the self-reported diagnosis dataset from the UK Biobank, ascertained through the completion of questionnaires and interviews with study participants (data field 20002 Non-cancer illness code, self-reported). We used 11 disease codes, which relate to immune-mediated diseases with known HLA associations (Table S9). For each disease, locus and allele we assumed an additive model in logistic regression with covariates including 25 principal components (Section S 3.3.2) to correct for population structure, self-reported sex, and genotyping array. Association tests were performed in the R programming language[25]. The results are shown in Table S9. For the fine-mapping replication analysis (Table 1) we included the same covariates.

| Dataset | Description |
|---|---|
| CEU+58 | Dataset composed of the British 1958 Birth Cohort, HapMap CEU individuals, and CEPH CEU+ additional individuals[26] |
| GSK | Dataset provided by GlaxoSmithKline (also known as 'HLA_RES')[26] |
| YRI | HapMap YRI individuals[26] |
| 1000G | 1000 Genomes Project dataset[8,27] |
| T1DGC | Type 1 Diabetes Genetics Consortium dataset[28] |
| KC | African-American individuals provided by King's College, London, UK (unpublished). |
| SW | Swedish individuals provided by Karolinska Institutet, Sweden (unpublished). |
| PA | Pan-Asian dataset made available by Pillai et al (2014)[29]. |

**Table S5 | Reference datasets utilised for HLA imputation in the UK Biobank cohort.**

| HLA locus | Reference datasets merged | Number of SNPs used | Number of reference individuals | Number of reference Europeans | Number of reference Africans/African Americans | Number of reference Asians | Number of reference Latinos |
|---|---|---|---|---|---|---|---|
| *HLA-A* | CEU+58,GSK,YRI,1000G,T1DGC | 661 | 8,085 | 7,347 | 208 | 307 | 223 |
| *HLA-B* | CEU+58,GSK,YRI,1000G,T1DGC | 927 | 9,120 | 8,112 | 236 | 417 | 355 |
| *HLA-C* | CEU+58,GSK,YRI,1000G,T1DGC | 908 | 7,732 | 6,984 | 212 | 313 | 223 |
| *HLA-DRB1* | CEU+58,GSK,YRI,1000G,T1DGC | 626 | 8,869 | 7,896 | 226 | 403 | 344 |
| *HLA-DRB3* | GSK,KC,SW | 849 | 880 | 484 | 345 | 17 | 34 |
| *HLA-DRB4* | GSK,KC,SW | 849 | 865 | 467 | 346 | 20 | 32 |
| *HLA-DRB5* | GSK,KC,SW | 801 | 808 | 408 | 346 | 18 | 36 |
| *HLA-DQA1* | CEU+58,GSK,YRI,T1DGC,PA | 747 | 6,242 | 5,640 | 27 | 503 | 72 |
| *HLA-DQB1* | CEU+58,GSK,YRI,1000G,T1DGC | 623 | 8,491 | 7,676 | 217 | 335 | 263 |
| *HLA-DPA1* | T1DGC,PA,SW | 794 | 6,067 | 5,615 | 0 | 452 | 0 |
| *HLA-DPB1* | GSK,T1DGC,PA,SW | 691 | 6,176 | 5,687 | 0 | 463 | 26 |

**Table S6 | The number of SNPs and samples used for each HLA locus in the imputation analysis.**

| HLA locus | Europeans | Africans/African American | Asians | Latinos |
|---|---|---|---|---|
| *HLA-A* | 97.2 | 94.4 | 89.2 | 90.5 |
| *HLA-B* | 94 | 81.7 | 86.3 | 74.5 |
| *HLA-C* | 97.8 | 92.8 | 94.4 | 94.1 |
| *HLA-DRB1* | 93.9 | 87.9 | 87.6 | 82.4 |
| *HLA-DRB3* | 97.8 | 96.5 | 93.1 | 94.7 |
| *HLA-DRB4* | 97.7 | 98.4 | 100 | 100 |
| *HLA-DRB5* | 99.2 | 99.3 | 100 | 100 |
| *HLA-DQA1* | 98.4 | 94 | 94.8 | 81.6 |
| *HLA-DQB1* | 97.8 | 87.6 | 95.1 | 92.5 |
| *HLA-DPA1* | 99.5 | - | 98.8 | - |
| *HLA-DPB1* | 94.5 | - | 86.2 | 88.5 |

**Table S7 | Estimate of four-digit (two-field) accuracy (%) of HLA imputation with a posterior probability call threshold of 0.**

| HLA locus | Europeans | Africans/African American | Asians | Latinos |
|---|---|---|---|---|
| *HLA-A* | 97.8/98.7 | 95.8/97.6 | 91.7/94.3 | 91.4/97.3 |
| *HLA-B* | 96.5/95.5 | 89.3/87.5 | 92.4/88.7 | 85.8/80.7 |
| *HLA-C* | 98.2/98.9 | 94.7/95.4 | 95.0/98.6 | 95.9/98.0 |
| *HLA-DRB1* | 96.3/95.3 | 90.8/92.8 | 92.6/91.0 | 89.9/85.9 |
| *HLA-DRB3* | 98.0/99.5 | 97.3/97.7 | 93.1/100.0 | 96.4/96.5 |
| *HLA-DRB4* | 98.2/98.6 | 98.8/98.4 | 100.0/96.8 | 100.0/100.0 |
| *HLA-DRB5* | 99.5/99.0 | 99.6/99.6 | 100.0/96.9 | 100.0/100.0 |
| *HLA-DQA1* | 98.8/99.1 | 97.9/94.0 | 96.2/98.0 | 82.9/97.4 |
| *HLA-DQB1* | 98.4/98.6 | 90.6/94.3 | 95.9/97.4 | 93.1/97.0 |
| *HLA-DPA1* | 99.6/99.7 | - | 99.0/99.6 | - |
| *HLA-DPB1* | 96.1/95.1 | - | 89.5/92.8 | 88.2/98.1 |

**Table S8 | Estimate of four-digit (two-field) accuracy (%) of HLA imputation/Call rate (%) with a posterior probability call threshold of 0.7.** The relevant sample sizes are given in Table S6. The accuracy and call rate estimates were obtained via five-fold cross validation

| Disease | Top Allele in UKBB | UKBB Allele frequency | OR (95% C.I.) | *p*-value | Reported most significant allele or haplotype | Note | Number of cases |
|---|---|---|---|---|---|---|---|
| Psoriasis | *HLA-C*06:02* | 0.090 | 3.61 (3.45 - 3.79) | $<1 \times 10^{-600}$ | *HLA-C*06:02* [30] | Top allele identified | 4802 |
| Malabsorption/coeliac disease | *HLA-DQB1*02:01* | 0.150 | 5.83 (5.45 - 6.23) | $<1 \times 10^{-600}$ | DR3-DQ2 haplotype [31] | *HLA-DQB1*02:01* is part of the DR3-DQ2 haplotype | 1799 |
| Ankylosing spondylitis | *HLA-B*27:05* | 0.040 | 10.13 (9.20 - 11.14) | $<1 \times 10^{-600}$ | *HLA-B*27:05* [32] | Top allele identified | 1183 |
| Rheumatoid arthritis | *HLA-DQA1*03:01* | 0.204 | 1.77 (1.70 - 1.85) | $1.50 \times 10^{-131}$ | *HLA-DRB1*04:01* [33] | *HLA-DQA1*03:01* and *HLA-DRB1*04:01* are in moderate LD ($r^2$ = 0.50). *HLA-DRB1*04:01* was the second most significant allele observed in UK Biobank | 4727 |
| Multiple sclerosis | *HLA-DRB5*01:01* | 0.145 | 2.56 (2.36 - 2.77) | $4.00 \times 10^{-105}$ | *HLA-DRB1*15:01* [34] | *HLA-DRB5*01:01* is in LD with *HLA-DRB1*15:01* ($r^2$ = 0.95) | 1503 |
| Asthma | *HLA-DQA1*03:01* | 0.204 | 1.17 (1.15 - 1.19) | $2.00 \times 10^{-77}$ | *HLA-DQ* [35] | Individual alleles in HLA-DQ not fine-mapped in reviewed literature | 47860 |
| Ulcerative colitis | *HLA-DRB1*01:03* | 0.017 | 3.13 (2.73 - 3.58) | $2.70 \times 10^{-45}$ | *HLA-DRB1*01:03* [36] | Top allele identified | 2219 |
| Type 1 diabetes | *HLA-DQB1*03:02* | 0.104 | 3.26 (2.76 - 3.84) | $3.20 \times 10^{-37}$ | *HLA-DQB1*03:02* [37] | Top allele identified | 363 |
| Crohn's disease | *HLA-DRB1*01:03* | 0.017 | 2.81 (2.32 - 3.40) | $4.80 \times 10^{-20}$ | *HLA-DRB1*01:03* [36] | Top allele identified | 1255 |
| Sjögren's syndrome/Sicca syndrome | *HLA-B*08:01* | 0.145 | 1.98 (1.68 - 2.33) | $1.10 \times 10^{-14}$ | *HLA-DQB1*02:01* [38] | *HLA-DQB1*02:01* and *HLA-B*08:01* are in moderate LD ($r^2$ = 0.50). *HLA-B*08:01* was the third most significant allele in [38] | 387 |
| Systemic lupus erythematosis | *HLA-DRB1*03:01* | 0.149 | 1.84 (1.58 - 2.14) | $8.80 \times 10^{-14}$ | *HLA-DRB1*03:01* [39] | Top allele identified | 452 |

**Table S9 | Primary HLA associations with disease in the self-reported diagnosis data field (20002) of the UK Biobank cohort.** (n=409,724). Association analysis was performed with logistic regression assuming an additive effect of the HLA allele. P-values were calculated with the likelihood ratio test compared to the null model of no HLA allele effect.

# S 6 Details of genome-wide association tests for QC

To demonstrate the quality of the directly genotyped and imputed data, we conducted a genome-wide association scan for a well-studied human trait: standing height. Large cohorts and large-scale meta-analyses already exist for this trait, thus providing an independent comparison set for our scan.

We conducted the scan using the directly genotyped and imputed data in the form that they are made available to researchers, but with a subset of samples. Specifically, we only included samples with all of the following properties:

- Imputation was carried out on them.
- In the white British ancestry subset (see Section S 3.4).
- Inferred sex matches self-reported sex.

From this group we selected a set of 344,397 unrelated individuals (see Section S 3.7.4). For standing height, a further 1,076 individuals were excluded due to missing values for the phenotype, leaving a total of 343,321 for association testing.

We used the software *BOLT-LMM* (v2.2)[40] to look for evidence of statistical association between each marker and standing height. We report association statistics based on a linear mixed model with the following covariates.

- Array (UK BiLEVE Axiom Array or UK Biobank Axiom Array).
- Sex (inferred).
- Age when attended UK Biobank assessment centre.
- Principal components 1-20.

The principal components scores were computed using only individuals within the white British ancestry subset, but otherwise with the same method as described in Section S 3.3.2. We conducted tests using the genotype and imputed data files separately.

Results of this analysis, and a comparison to an independent study of 253,288 individuals of European ancestry carried out by the Genetic Investigation of Anthropometric Traits Consortium (GIANT)[41], are discussed in the main text (see also Figure 4, Figure S16).

We also analysed a second phenotype, "Intra-ocular pressure", which has fewer previously-reported regions of association than standing height[42]. Results of this analysis are shown in Figure S16.

## S 6.1 Defining regions of association for the comparison with GIANT

We first define a region 0.125 centimorgans (cM) plus 25 Kb each side of the marker with the smallest *p*-value in GIANT, using the HapMap recombination map. We keep

the region only if it also contains a marker with a *p*-value < $5x10^{-8}$ in the UK Biobank imputed data. We consider the marker with the next smallest *p*-value in GIANT that is not in that region, define a new region around it using the same criteria, and keep the new region unless it overlaps with any of the previously accepted regions in the sequence. We consider the marker with next smallest *p*-value outside any previously considered region, and so on until there are no more genome-wide significant markers in GIANT. This procedure resulted in 575 non-overlapping associated regions. There were 725 regions if we allowed overlaps and ignored the UK Biobank data. Of these, 57 were not genome-wide significant in UK Biobank and a further 93 were excluded due to overlaps.

We used these regions for the credible set analysis detailed in Methods "Comparison of GIANT and UK Biobank GWAS results" (see also Extended Data Figure 6). To compute marker-specific Bayes factors in favour of association with standing height we used the effect sizes and standard errors. That is, the reciprocal of equation (4) in [43].

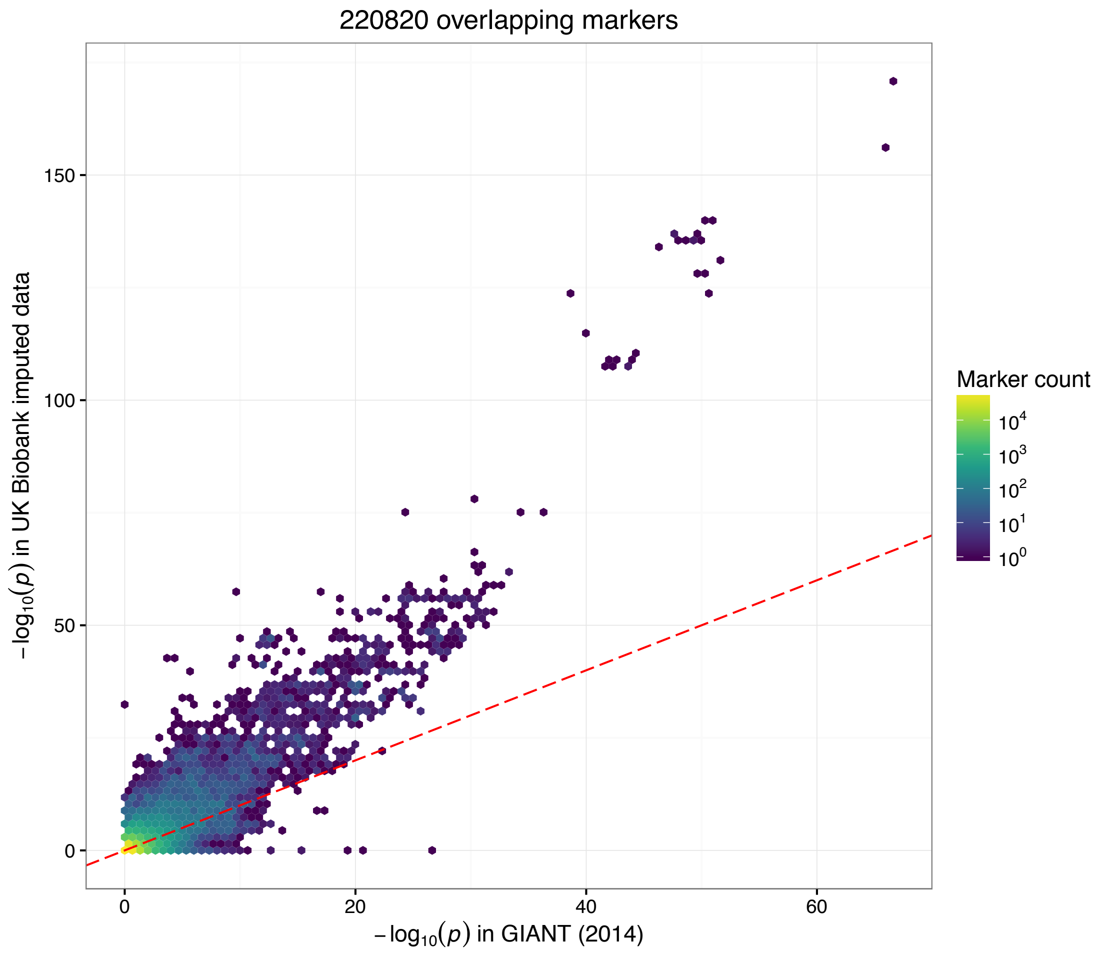


**Figure S16 | Comparison of p-values for UK Biobank and GIANT in standing height GWAS.** Each point is a marker on chromosome 2 that was included in both UK Biobank imputed data (n=343,321 samples) and GIANT (2014) (n=253,288). UK Biobank p-values we calculated using a linear mixed model. We identified markers common to both studies by matching on chromosome, position, and the two alleles. The red line shows x=y.

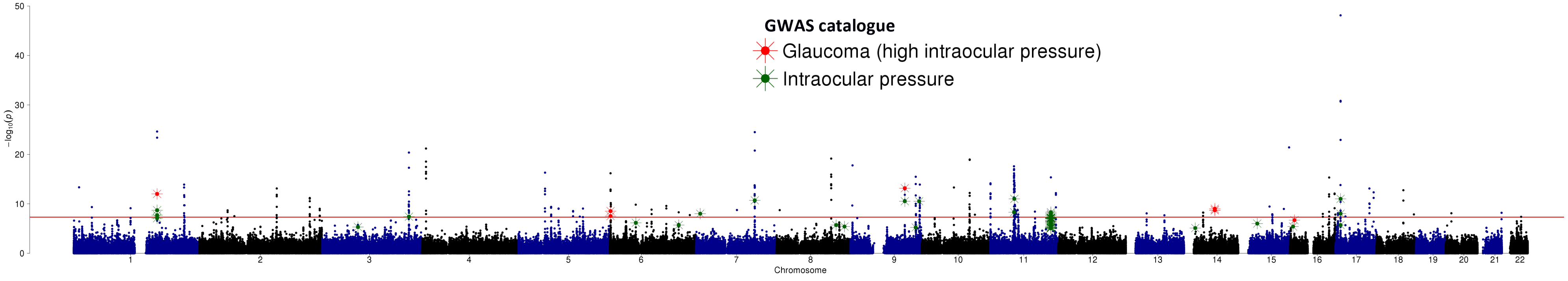


**Figure S17 | Results of a GWAS for mean intra-ocular pressure (Goldmann correlated).** This plot shows association statistics (p-values from a linear mixed model) for a GWAS using genotype data for 70,880 UK Biobank participants who had intra-ocular pressure measured for both eyes. We used the mean value as the phenotype. We used the same analysis procedure as for standing height (see Section S 6), except we first quantile normalised the phenotype data before running the association scan to reduce the influence of outlying values.  Associations reported in the NCBI GWAS catalogue (as of April 2017) for two closely related phenotypes are shown in red and green, at the p-value reported in the catalogue.

# S 7 Multiple trait GWAS and PheWAS

To facilitate the running of GWAS for multiple continuous traits and fast phenome wide association studies (PheWAS) we have provided a new tool called BGENIE https://jmarchini.org/bgenie/ that is built upon the BGEN library http://www.well.ox.ac.uk/~gav/bgen_format/

BGENIE is designed to work well when analysing multiple phenotypes. The program takes bgen files as input and avoids repeated decompression and conversion of these files when analysing multiple phenotypes. In contrast, although PLINK1.9 and PLINK2.0 can carryout GWAS of multiple traits, when presented with bgen files they will first convert the bgen files to temporary files in the bed or pgen format before running the GWAS separately on those temporary files for each phenotype, which can use a lot of disk space. Plink 2.0 reads bgen files directly.

We assessed the performance of BGENIE, PLINK1.9 and PLINK2.0 on bgen files on both the interim release (152,249 subjects) and the full release (487,409 subjects) and by analysing 25,000 SNPs from a single chromosome. We created simulated phenotype files with P=1, 50, 500 phenotypes to explore the performance as the number of phenotypes increases. All of the analysis was run on a 16 core machine with Xeon E5-2690 2.90GHz CPUs. All programs were run using 8 threads. The results are presented in Table S10 and shows that that BGENIE can be considerably faster that PLINK 1.9 in all settings and faster than PLINK 2.0 when the number of phenotypes is large.

| | Interim release 152,249 subjects | | | Full UKB release 487,409 subjects | | |
|---|---|---|---|---|---|---|
| P | PLINK v1.9b4.4 | PLINK v2.0a | BGENIE v1.1 | PLINK v1.9b4.4 | PLINK v2.0a | BGENIE v1.1 |
| 1 | 173s | 25s | 85s | 603s | 65s | 439s |
| 50 | 8,600s | 317s | 165s | 24,206s | 1,397s | 752s |
| 500 | 78,064s | 3,023s | 835s | 227,622s | 13,507s | 5,861s |

**Table S10 | Run time comparison of PLINK and BGENIE.** Results are run time in seconds for processing 25,000 SNPs for different numbers of phenotypes (P).

Not all of the computations in BGENIE are currently carried out using multiple threads so researchers should be aware of how scaling works. Table S11 shows the results from analyzing a single phenotype using the interim release dataset with 152,249 subjects using different numbers of threads.

| Threads | 1 | 2 | 4 | 8 | 10 | 12 | 14 |
|---|---|---|---|---|---|---|---|
| SNPs/s | 93 | 116 | 215 | 283 | 322 | 355 | 373 |

**Table S11 | Run time analysis of BGENIE when varying the number of threads used.**

BGEN files can be indexed using the BGENIX tool

(https://bitbucket.org/gavinband/bgen/wiki/bgenix)

This facilitates fast access of individual SNPs and regions using BGENIE and this is useful when researchers wish to investigate a single SNP or region for association with a single phenotype or for carrying out PheWAS. We found that using PLINK 2.0 took ~2,500 seconds to read and analyze a single SNP from the interim release (152,249 subjects) for analysis of 500 phenotypes. For the same analysis BGENIE took ~83 seconds.

To illustrate this type of analysis we carried out a GWAS of brain imaging derived phenotypes (IDPs) using the interim release dataset. We identified SNP rs35430475 as potentially associated with an IDP derived from the diffusion weighted imaging which measures different aspects of the brain white matter microarchitecture. The specific phenotype was the intra- cellular volume fraction in the right Cingulum hippocampus and was measured using tract based spatial statistics (TBSS). The PheWAS for this SNP with all 951 IDPs is shown in Figure S18, and suggests that this SNP maybe associated with various phenotypes related to white matter microarchitecture.

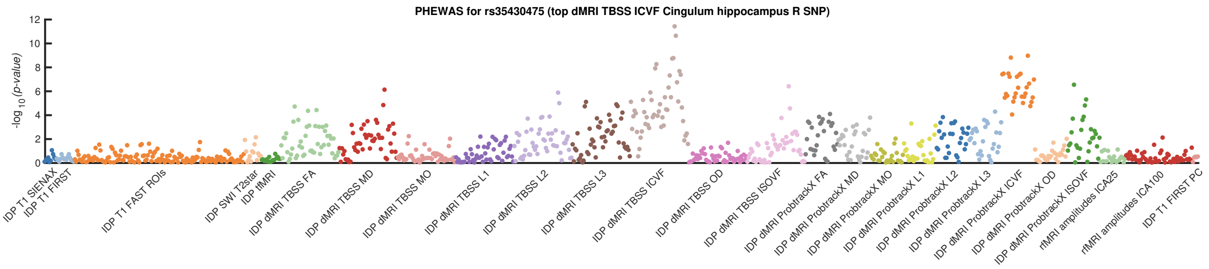


**Figure S18 | Results of a PheWAS analysis for SNP rs35430475 identified as interesting from a GWAS of a brain imaging derived phenotypes (IDP) [44].** The PheWAS was carried out using 951 IDPs measured on 2,807 subjects. IDPs have been colour coded by type in the plot.

# S 8 References


1 Wain, L. V. *et al.* Novel insights into the genetics of smoking behaviour, lung function, and chronic obstructive pulmonary disease (UK BiLEVE): a genetic association study in UK Biobank. *The Lancet Respiratory Medicine* **3**, 769--781, doi:10.1016/S2213-2600(15)00283-0.

2 Welsh, S. Genotyping of 500,000 UK Biobank participants: Description of sample processing workflow and preparation of DNA for genotyping. (http://biobank.ndph.ox.ac.uk/showcase/refer.cgi?id=807).

3 UK Biobank Axiom Array Content Summary. (http://www.ukbiobank.ac.uk/wp-content/uploads/2014/04/UK-Biobank-Axiom-Array-Content-Summary-2014.pdf).

4 Affymetrix. UKB_WCSGAX: UK Biobank 500K Samples Genotyping Data Generation by the Affymetrix Research Services Laboratory. (http://biobank.ndph.ox.ac.uk/showcase/refer.cgi?id=368).

5 Elliott, P. & Peakman, T. C. The UK Biobank sample handling and storage protocol for the collection, processing and archiving of human blood and urine. *Int J Epidemiol* **37**, doi:10.1093/ije/dym276 (2008).

6 Welsh, S., Peakman, T., Sheard, S. & Almond, R. Comparison of DNA quantification methodology used in the DNA extraction protocol for the UK Biobank cohort. *BMC Genomics* **18**, 26, doi:10.1186/s12864-016-3391-x (2017).

7 Affymetrix. UKB_WCSGAX: UK Biobank 500K Samples Processing by the Affymetrix Research Services Laboratory. (http://biobank.ndph.ox.ac.uk/showcase/refer.cgi?id=590).

8 The 1000 Genomes Project Consortium. An integrated map of genetic variation from 1,092 human genomes. *Nature* **491**, 56--65 (2012).

9 Shaun Purcell, C. C. PLINK v1.9. <https://www.cog-genomics.org/plink2>.

10 Bellenguez, C. *et al.* A robust clustering algorithm for identifying problematic samples in genome-wide association studies. *Bioinformatics* **28**, 134-135, doi:10.1093/bioinformatics/btr599 (2012).

11 Wigginton, J. E., Cutler, D. J. & Abecasis, G. R. A Note on Exact Tests of Hardy-Weinberg Equilibrium. *American Journal of Human Genetics* **76**, 887--893 (2005).

12 Chang, C. C. *et al.* Second-generation PLINK: rising to the challenge of larger and richer datasets. *GigaScience* **4**, 7, doi:10.1186/s13742-015-0047-8 (2015).

13 Affymetrix. Axiom® Genotyping Solution Data Analysis Guide. (http://tools.thermofisher.com/content/sfs/manuals/axiom_genotyping_solution_analysis_guide.pdf).

14 Balding, D. J. A tutorial on statistical methods for population association studies. *Nat Rev Genet* **7**, 781-791, doi:10.1038/nrg1916 (2006).

15 Price, A. L. *et al.* Principal components analysis corrects for stratification in genome-wide association studies. *Nat Genet* **38**, 904-909, doi:10.1038/ng1847 (2006).

16 Manichaikul, A. *et al.* Robust relationship inference in genome-wide association studies. *Bioinformatics* **26**, 2867-2873, doi:10.1093/bioinformatics/btq559 (2010).

17 Galinsky, K. J. *et al.* Fast Principal-Component Analysis Reveals Convergent Evolution of ADH1B in Europe and East Asia. *The American Journal of Human Genetics* **98**, 456--472, doi:10.1016/j.ajhg.2015.12.022 (2016).
18 The International Multiple Sclerosis Genetics Consortium & The Wellcome Trust Case Control Consortium 2 *et al.* Genetic risk and a primary role for cell-mediated immune mechanisms in multiple sclerosis. *Nature* **476**, 214-219, doi:10.1038/nature10251 (2011).
19 Affymetrix® Axiom CNV Summary Tool (Affymetrix, 2013).
20 Nielsen, J. & Wohlert, M. Chromosome abnormalities found among 34910 newborn children: results from a 13-year incidence study in Århus, Denmark. *Human Genetics* **87**, 81-83, doi:10.1007/BF01213097 (1991).
21 Csardi, G. & Nepusz, T. The igraph software package for complex network research. *InterJournal* **Complex Systems**, 1695 (2006).
22 Fry, A. *et al.* Comparison of Sociodemographic and Health-Related Characteristics of UK Biobank Participants with the General Population. *Am J Epidemiol*, doi:10.1093/aje/kwx246 (2017).
23 McCarthy, S. *et al.* A reference panel of 64,976 haplotypes for genotype imputation. *Nat Genet* **48**, 1279-1283, doi:10.1038/ng.3643 (2016).
24 The UK10K Consortium. The UK10K project identifies rare variants in health and disease. *Nature* **526**, 82-90, doi:10.1038/nature14962 (2015).
25 R: A Language and Environment for Statistical Computing (R Foundation for Statistical Computing, Vienna, Austria, 2014).
26 Dilthey, A. *et al.* Multi-Population Classical HLA Type Imputation. *PLoS Computational Biology* **9**, e1002877, doi:10.1371/journal.pcbi.1002877 (2013).
27 Gourraud, P.-A. *et al.* HLA Diversity in the 1000 Genomes Dataset. *PLOS ONE* **9**, e97282, doi:10.1371/journal.pone.0097282 (2014).
28 Jia, X. *et al.* Imputing Amino Acid Polymorphisms in Human Leukocyte Antigens. *PLOS ONE* **8**, e64683, doi:10.1371/journal.pone.0064683 (2013).
29 Pillai, N. E. *et al.* Predicting HLA alleles from high-resolution SNP data in three Southeast Asian populations. *Hum Mol Genet* **23**, 4443--4451, doi:10.1093/hmg/ddu149 (2014).
30 Okada, Y. *et al.* Fine mapping major histocompatibility complex associations in psoriasis and its clinical subtypes. *Am J Hum Genet* **95**, 162-172, doi:10.1016/j.ajhg.2014.07.002 (2014).
31 Gutierrez-Achury, J. *et al.* Fine mapping in the MHC region accounts for 18% additional genetic risk for celiac disease. *Nat Genet* **47**, 577-578, doi:10.1038/ng.3268 (2015).
32 Cortes, A. *et al.* Major histocompatibility complex associations of ankylosing spondylitis are complex and involve further epistasis with ERAP1. *Nat Commun* **6**, 7146, doi:10.1038/ncomms8146 (2015).
33 Raychaudhuri, S. *et al.* Five amino acids in three HLA proteins explain most of the association between MHC and seropositive rheumatoid arthritis. *Nat Genet* **44**, 291-296, doi:10.1038/ng.1076 (2012).
34 The International Multiple Sclerosis Genetics Consortium. Class II HLA interactions modulate genetic risk for multiple sclerosis. *Nat Genet* **47**, 1107-1113, doi:10.1038/ng.3395 (2015).

35 Moffatt, M. F. *et al.* A large-scale, consortium-based genomewide association study of asthma. *The New England journal of medicine* **363**, 1211-1221, doi:10.1056/NEJMoa0906312 (2010).
36 Goyette, P. *et al.* High-density mapping of the MHC identifies a shared role for HLA-DRB1*01:03 in inflammatory bowel diseases and heterozygous advantage in ulcerative colitis. *Nat Genet* **47**, 172-179, doi:10.1038/ng.3176 (2015).
37 Hu, X. *et al.* Additive and interaction effects at three amino acid positions in HLA-DQ and HLA-DR molecules drive type 1 diabetes risk. *Nat Genet* **47**, 898-905, doi:10.1038/ng.3353 (2015).
38 Lessard, C. J. *et al.* Variants at multiple loci implicated in both innate and adaptive immune responses are associated with Sjogren's syndrome. *Nat Genet* **45**, 1284-1292, doi:10.1038/ng.2792 (2013).
39 Morris, D. L. *et al.* Unraveling multiple MHC gene associations with systemic lupus erythematosus: model choice indicates a role for HLA alleles and non-HLA genes in Europeans. *Am J Hum Genet* **91**, 778-793, doi:10.1016/j.ajhg.2012.08.026 (2012).
40 Loh, P.-R. *et al.* Efficient Bayesian mixed-model analysis increases association power in large cohorts. *Nat Genet* **47**, 284--290 (2015).
41 Wood, A. R. *et al.* Defining the role of common variation in the genomic and biological architecture of adult human height. *Nat Genet* **46**, 1173--1186 (2014).
42 Welter, D. *et al.* The NHGRI GWAS Catalog, a curated resource of SNP-trait associations. *Nucleic Acids Research* **42**, D1001--D1006 (2014).
43 Wakefield, J. Commentary: Genome-wide significance thresholds via Bayes factors. *International Journal of Epidemiology* **41**, 286-291, doi:10.1093/ije/dyr241 (2012).
44 Miller, K. L. *et al.* Multimodal population brain imaging in the UK Biobank prospective epidemiological study. *Nat Neurosci* **19**, 1523--1536 (2016).
45 Price, A. L. *et al.* Long-range LD can confound genome scans in admixed populations. *Am J Hum Genet* **83**, 132-135; author reply 135-139, doi:10.1016/j.ajhg.2008.06.005 (2008).

# S 9 Appendix

| Genotyping array | Batch | Batch size | Number of processed plates | Median plate size within a batch |
|---|---|---|---|---|
| **Applied Biosystems™ UK BiLEVE Axiom™ Array** | UKBiLEVEAX_b1 | 4536 | 52 | 90 |
| | UKBiLEVEAX_b2 | 4545 | 53 | 90 |
| | UKBiLEVEAX_b3 | 4520 | 56 | 89 |
| | UKBiLEVEAX_b4 | 4542 | 52 | 89 |
| | UKBiLEVEAX_b5 | 4524 | 58 | 88 |
| | UKBiLEVEAX_b6 | 4524 | 60 | 89 |
| | UKBiLEVEAX_b7 | 4524 | 60 | 88.5 |
| | UKBiLEVEAX_b8 | 4551 | 53 | 90 |
| | UKBiLEVEAX_b9 | 4530 | 54 | 89 |
| | UKBiLEVEAX_b10 | 4559 | 58 | 88 |
| | UKBiLEVEAX_b11 | 4595 | 71 | 86 |
| **Applied Biosystems™ UK Biobank Axiom™ Array** | Batch_b001 | 4683 | 52 | 92 |
| | Batch_b002 | 4646 | 74 | 68.5 |
| | Batch_b003 | 4642 | 83 | 59 |
| | Batch_b004 | 4642 | 91 | 53 |
| | Batch_b005 | 4655 | 84 | 59.5 |
| | Batch_b006 | 4675 | 64 | 78.5 |
| | Batch_b007 | 4670 | 74 | 67 |
| | Batch_b008 | 4743 | 186 | 19 |
| | Batch_b009 | 4679 | 73 | 82 |
| | Batch_b010 | 4691 | 85 | 58 |
| | Batch_b011 | 4692 | 96 | 63.5 |
| | Batch_b012 | 4686 | 83 | 86 |
| | Batch_b013 | 4679 | 56 | 84 |
| | Batch_b014 | 4689 | 201 | 10 |
| | Batch_b015 | 4677 | 465 | 4 |
| | Batch_b016 | 4552 | 171 | 7 |
| | Batch_b017 | 4526 | 132 | 8 |
| | Batch_b018 | 4578 | 108 | 32.5 |
| | Batch_b019 | 4573 | 129 | 11 |
| | Batch_b020 | 4611 | 86 | 68.5 |
| | Batch_b021 | 4548 | 134 | 9 |
| | Batch_b022 | 4695 | 79 | 84 |
| | Batch_b023 | 4660 | 97 | 53 |
| | Batch_b024 | 4650 | 112 | 19.5 |
| | Batch_b025 | 4664 | 88 | 72.5 |
| | Batch_b026 | 4662 | 91 | 64 |
| | Batch_b027 | 4652 | 50 | 93.5 |
| | Batch_b028 | 4659 | 74 | 89 |

| Genotyping array | Batch | Batch size | Number of processed plates | Median plate size within a batch |
|---|---|---|---|---|
| | Batch_b029 | 4658 | 51 | 93 |
| | Batch_b030 | 4650 | 58 | 92.5 |
| | Batch_b031 | 4659 | 54 | 92 |
| | Batch_b032 | 4690 | 72 | 88 |
| | Batch_b033 | 4667 | 50 | 93.5 |
| | Batch_b034 | 4628 | 50 | 93 |
| | Batch_b035 | 4631 | 50 | 93 |
| | Batch_b036 | 4658 | 50 | 93.5 |
| | Batch_b037 | 4651 | 50 | 94 |
| | Batch_b038 | 4638 | 79 | 91 |
| | Batch_b039 | 4602 | 74 | 91.5 |
| | Batch_b040 | 4665 | 50 | 94 |
| | Batch_b041 | 4622 | 61 | 80 |
| | Batch_b042 | 4643 | 50 | 93 |
| | Batch_b043 | 4648 | 62 | 89.5 |
| | Batch_b044 | 4677 | 51 | 93 |
| | Batch_b045 | 4661 | 53 | 93 |
| | Batch_b046 | 4656 | 70 | 90 |
| | Batch_b047 | 4642 | 84 | 74 |
| | Batch_b048 | 4643 | 91 | 70 |
| | Batch_b049 | 4635 | 64 | 90 |
| | Batch_b050 | 4633 | 59 | 91 |
| | Batch_b051 | 4631 | 64 | 92 |
| | Batch_b052 | 4586 | 136 | 7.5 |
| | Batch_b053 | 4613 | 100 | 40.5 |
| | Batch_b054 | 4608 | 80 | 87.5 |
| | Batch_b055 | 4626 | 70 | 92 |
| | Batch_b056 | 4615 | 69 | 92 |
| | Batch_b057 | 4617 | 54 | 93 |
| | Batch_b058 | 4652 | 58 | 93 |
| | Batch_b059 | 4652 | 52 | 93 |
| | Batch_b060 | 4610 | 55 | 92 |
| | Batch_b061 | 4616 | 57 | 92 |
| | Batch_b062 | 4619 | 67 | 91 |
| | Batch_b063 | 4625 | 63 | 91 |
| | Batch_b064 | 4627 | 84 | 86 |
| | Batch_b065 | 4646 | 55 | 93 |
| | Batch_b066 | 4630 | 55 | 92 |
| | Batch_b067 | 4622 | 59 | 92 |
| | Batch_b068 | 4641 | 60 | 90 |
| | Batch_b069 | 4634 | 64 | 91 |

| Genotyping array | Batch | Batch size | Number of processed plates | Median plate size within a batch |
|---|---|---|---|---|
| | Batch_b070 | 4657 | 56 | 92.5 |
| | Batch_b071 | 4610 | 52 | 92 |
| | Batch_b072 | 4618 | 56 | 92 |
| | Batch_b073 | 4640 | 64 | 92 |
| | Batch_b074 | 4641 | 54 | 91 |
| | Batch_b075 | 4644 | 59 | 92 |
| | Batch_b076 | 4632 | 59 | 90 |
| | Batch_b077 | 4643 | 53 | 91 |
| | Batch_b078 | 4638 | 58 | 90 |
| | Batch_b079 | 4647 | 61 | 91 |
| | Batch_b080 | 4660 | 60 | 86 |
| | Batch_b081 | 4636 | 64 | 85 |
| | Batch_b082 | 4647 | 64 | 84 |
| | Batch_b083 | 4629 | 68 | 84 |
| | Batch_b084 | 4664 | 65 | 82 |
| | Batch_b085 | 4649 | 66 | 84.5 |
| | Batch_b086 | 4651 | 69 | 87 |
| | Batch_b087 | 4660 | 61 | 88 |
| | Batch_b088 | 4664 | 69 | 88 |
| | Batch_b089 | 4647 | 71 | 88 |
| | Batch_b090 | 4658 | 60 | 90 |
| | Batch_b091 | 4626 | 66 | 86.5 |
| | Batch_b092 | 4663 | 58 | 92 |
| | Batch_b093 | 4626 | 70 | 87.5 |
| | Batch_b094 | 2203 | 59 | 10 |
| | Batch_b095 | 4468 | 258 | 1 |
| | All batches | **488377** | **5625** | **84** |

**Table S12 | Number of participants genotyped within each batch.** Intensities for each marker were measured on 96-well plates in groups of 94 UK Biobank samples and two control samples. The intensity data for multiple plates were combined to form batches of ~4,700 UK Biobank samples, and genotypes were called *in silico* within each batch. In some cases samples from the same plate were genotyped in different batches, so the total number of unique plates is smaller than the sum of column 3, and the median plate size within each batch is often less than 94. Batches labelled with the prefix "UKBiLEVEAX" contain only samples typed using the UK BiLEVE Axiom array, and those with the prefix "Batch" contain only samples typed using the UK Biobank Axiom array.

| Chromosome | Start position (bp) | End position (bp) |
|---|---|---|
| **1** | 48000000 | 52000000 |
| **2** | 86000000 | 100500000 |
| **2** | 134500000 | 138000000 |
| **2** | 183000000 | 190000000 |
| **3** | 47500000 | 50000000 |
| **3** | 83500000 | 87000000 |
| **3** | 89000000 | 97500000 |
| **5** | 44000000 | 51500000 |
| **5** | 98000000 | 100500000 |
| **5** | 129000000 | 132000000 |
| **5** | 135500000 | 138500000 |
| **6** | 25000000 | 33500000 |
| **6** | 57000000 | 64000000 |
| **6** | 140000000 | 142500000 |
| **7** | 55000000 | 66000000 |
| **8** | 8000000 | 12000000 |
| **8** | 43000000 | 50000000 |
| **10** | 37000000 | 43000000 |
| **11** | 45000000 | 57000000 |
| **11** | 87500000 | 90500000 |
| **12** | 33000000 | 40000000 |
| **12** | 109500000 | 112000000 |
| **20** | 32000000 | 34500000 |

**Table S13 | Regions of long-range LD excluded from PCA.** These regions are based on those reported in [45]. Positions are in coordinates of human genome build GRCh37.
