# Supplementary material for: The UK Biobank resource with deep phenotyping and genomic data
Source: Nature. 2018 Oct 10;562(7726):203–9. doi: 10.1038/s41586-018-0579-z (PMC6786975; doi:10.1038/s41586-018-0579-z)
Supplement: Supplementary file 2 — Reporting Summary [file 41586_2018_579_MOESM2_ESM.pdf]

nature research

Corresponding author(s): Jonathan Marchini

Reporting Summary

Nature Research wishes to improve the reproducibility of the work that we publish. This form provides structure for consistency and transparency in reporting. For further information on Nature Research policies, see Authors & Referees and the Editorial Policy Checklist.

Statistical parameters

When statistical analyses are reported, confirm that the following items are present in the relevant location (e.g. figure legend, table legend, main text, or Methods section).

n/a	Confirmed	
☐	☒	The exact sample size (*n*) for each experimental group/condition, given as a discrete number and unit of measurement
☐	☒	An indication of whether measurements were taken from distinct samples or whether the same sample was measured repeatedly
☐	☒	The statistical test(s) used AND whether they are one- or two-sided<br>*Only common tests should be described solely by name; describe more complex techniques in the Methods section.*
☐	☒	A description of all covariates tested
☐	☒	A description of any assumptions or corrections, such as tests of normality and adjustment for multiple comparisons
☐	☒	A full description of the statistics including central tendency (e.g. means) or other basic estimates (e.g. regression coefficient) AND variation (e.g. standard deviation) or associated estimates of uncertainty (e.g. confidence intervals)
☒	☐	For null hypothesis testing, the test statistic (e.g. *F*, *t*, *r*) with confidence intervals, effect sizes, degrees of freedom and *P* value noted<br>*Give P values as exact values whenever suitable.*
☐	☒	For Bayesian analysis, information on the choice of priors and Markov chain Monte Carlo settings
☒	☐	For hierarchical and complex designs, identification of the appropriate level for tests and full reporting of outcomes
☐	☒	Estimates of effect sizes (e.g. Cohen's *d*, Pearson's *r*), indicating how they were calculated
☒	☐	Clearly defined error bars<br>*State explicitly what error bars represent (e.g. SD, SE, CI)*

Our web collection on statistics for biologists may be useful.

Software and code

Policy information about availability of computer code

Data collection	Genotype data was collected by Affymetrix using a highly customised version of the Affymetrix software suite Affymetrix Genotyping Console Software (GTC), Affymetrix Power Tools (APT) and SNPolisher R package
Data analysis	For quality control, ancestry and relatedness analyses, we mostly used off-the-shelf software combined into a pipeline of bash scripts and R scripts. Figures were created using R. Software or algorithms used in these analyses are described in the Methods and Supplementary Material. We include a list of links to key software packages below and in the URL section. Other software packages are referenced where appropriate. For custom code, we have endeavoured to describe the methodology in sufficient detail such that it could be reproduced accurately. All code used to perform the analyses in this study is either available from the corresponding author upon reasonable request or executables and documentation are available by following the URLs in the paper.<br><br>SHAPEIT3, IMPUTE4, BGENIE https://jmarchini.org/software/<br>Hapfuse https://bitbucket.org/wkretzsch/hapfuse<br>BGENIX, BGEN library https://bitbucket.org/gavinband/bgen<br>Evoker https://github.com/wtsi-medical-genomics/evoker<br>BGEN file format http://www.well.ox.ac.uk/~gav/bgen_format/bgen_format.html<br>SNPTEST https://mathgen.stats.ox.ac.uk/genetics_software/snptest/snptest.html<br>QCTOOL v2 - http://www.well.ox.ac.uk/~gav/qctool_v2<br>shellfish http://www.stats.ox.ac.uk/~davison/software/shellfish/shellfish.php<br>aberrant v.10 http://www.well.ox.ac.uk/software

PLINK v1.9 https://www.cog-genomics.org/plink/1.9/
KING v1.4 http://people.virginia.edu/~wc9c/KING/
fastPCA part of EIGENSOFT package v6.0.1 https://www.hsph.harvard.edu/alkes-price/software/ https://github.com/DReichLab/EIG/
BOLT-LMM v2.2 https://www.hsph.harvard.edu/alkes-price/software/
HLA*IMP02 https://oxfordhla.well.ox.ac.uk/hla/
igraph v1.0.1 http://igraph.org/r/

For manuscripts utilizing custom algorithms or software that are central to the research but not yet described in published literature, software must be made available to editors/reviewers upon request. We strongly encourage code deposition in a community repository (e.g. GitHub). See the Nature Research guidelines for submitting code & software for further information.

Data

Policy information about availability of data

All manuscripts must include a data availability statement. This statement should provide the following information, where applicable:

- Accession codes, unique identifiers, or web links for publicly available datasets
- A list of figures that have associated raw data
- A description of any restrictions on data availability

UK Biobank's Data Showcase (http://biobank.ctsu.ox.ac.uk/crystal/index.cgi) presents the univariate distributions, numbers of participants and methods used to collect each data item. Access to the resource is via submission of a short application form outlining the reason for the research and selection of the data-fields (http://www.ukbiobank.ac.uk/register-apply/). UK Biobank is a registered charity and data access charges are for cost-recovery purposes only (currently £2,500 for access to all genetic and phenotypic data per research project). Detailed information about the genetic data available from UK Biobank is available at http://www.ukbiobank.ac.uk/scientists-3/genetic-data/ and http://biobank.ctsu.ox.ac.uk/crystal/label.cgi?id=100314. The exact number of samples with genetic data currently available in UK Biobank may differ slightly from those described in this paper.

Field-specific reporting

Please select the best fit for your research. If you are not sure, read the appropriate sections before making your selection.

☒ Life sciences ☐ Behavioural & social sciences ☐ Ecological, evolutionary & environmental sciences

For a reference copy of the document with all sections, see nature.com/authors/policies/ReportingSummary-flat.pdf

Life sciences study design

All studies must disclose on these points even when the disclosure is negative.

Sample size

The UK Biobank genotype data analysed in this article comprises 488,377 samples. This is one of the largest human genetic datasets with extensive phenotyping available for research. The majority of existing datasets collected for genome-wide association studies have a few thousand samples. The large size clearly implies that it will be very well powered to detect genetic associations.

Those researchers who successfully apply for access to the UK Biobank genetic data may receive fewer samples than 488,377 due to participants withdrawing from the study since the analysis was carried out. Precise numbers of samples and genetic markers for different stages of the UK Biobank genotyping experiment are available in Extended Data Table 1.

Data exclusions

We summarise the numbers of SNPs and samples excluded in different stages of the UK Biobank genotyping experiment in Extended Table 2. Extensive details, including rationale, of SNP and sample QC are given in the Methods and Supplementary Material. Of the samples in the data delivery from Affymetrix, samples were excluded from the data release only if they were duplicates or because the participants had withdrawn from the study. Details of the exclusions (SNPs or samples) in each analysis (e.g. the standing height GWAS) are given in the methods section dedicated to each analysis.

Replication

This is a resource paper and there are no main findings. Rather we have described how the dataset was created. However we did seek to validate the quality of the data at several points in our analysis.

(a) we compared allele ferquencies of UK Biobank SNPs to those found in the ExAC dataset, showing very good agreement.

(b) For the imputation of ~96 million more variants we compared the performance of the UK Biobank Axiom array and several other commercially available genotyping arrays using separate samples sequenced at high-coverage, showing that the Axiom array performed very well in terms of imputation performance.

(c) For the example GWAS of standing height we compared the results to GIANT (see main text section "GWAS for standing height"), and other previously-reported association signals in the NHGRI-EBI GWAS catalogue. We were able to show a strong correlation between associated regions in both studies.

(d) For the HLA imputation we performed association tests for diseases known to have HLA associations, focusing on 11 self-reported immune-mediated diseases. For each disease in our analysis we identified the HLA allele with the strongest evidence of association, and in all cases these were consistent with previous reports (see Methods and Supplementary).

Randomization	Special attention was paid in the automated sample retrieval process at UK Biobank to ensure that experimental units such as plates or timing of extraction did not correlate systematically with baseline phenotypes such as age, sex, and ethnic background, or the time and location of sample collection. Further details are available in references 46 and 47.
Blinding	The UK Biobank study has a prospective design with many hundreds of phenotypes collected. Thus, there is no designated 'treatment' and 'control' groups, and many types of statistical analyses are possible. The quality control analysis, imputation, and association analyses reported in this article was carried out by researchers with only limited access to phenotype data (where required), and who had no influence over experimental processes in the laboratory, for example the assignment of samples to batches, or the participant recruitment process.

Reporting for specific materials, systems and methods

Materials & experimental systems

n/a	Involved in the study	
☒	☐	Unique biological materials
☒	☐	Antibodies
☒	☐	Eukaryotic cell lines
☒	☐	Palaeontology
☒	☐	Animals and other organisms
☐	☒	Human research participants

Methods

n/a	Involved in the study	
☒	☐	ChIP-seq
☒	☐	Flow cytometry
☒	☐	MRI-based neuroimaging

Human research participants

Policy information about studies involving human research participants

Population characteristics	The UK Biobank study population is residents of the UK aged 40-69 years at recruitment and living within a reasonable travelling distance of an assessment centre.
Recruitment	Participants were selected using the NHS register, and invited to volunteer for the study. Recruitment was carried out between 2007 and 2010. Full details of the recruitment process are available in reference 1 (UK Biobank: Protocol for a large-scale prospective epidemiological resource, 2007).
